# Supplementary material for: Distinctive features of lipoprotein profiles in stroke patients
Source: PLoS One. 2023 Apr 5;18(4):e0283855. doi: 10.1371/journal.pone.0283855 (PMC10075468; doi:10.1371/journal.pone.0283855)
Supplement: S1 File — (ZIP) [file pone.0283855.s001.zip › supplement/ind/box.htm]

Support


## Supporting information

for **Human lipoproteins as risk factors**  
  
Jump to S5 and S6.

## S5 Fig: Classes

### CM1

| TG | Cholesterol | TG/CH |
| --- | --- | --- |
|  |  |  |
| TukeyHSD   |  |  |  |  |  | | --- | --- | --- | --- | --- | | groups | diff | lwr | upr | p-adj | | 1-0 | -0.6810614 | -1.2125628 | -0.1495601 | 0.0059705 | | 2-0 | -0.3072650 | -0.8558540 | 0.2413241 | 0.4673244 | | 3-0 | -0.2138531 | -1.1003327 | 0.6726266 | 0.9233624 | | 2-1 | 0.3737965 | -0.1696187 | 0.9172117 | 0.2836347 | | 3-1 | 0.4672084 | -0.4160788 | 1.3504956 | 0.5175617 | | 3-2 | 0.0934119 | -0.8002617 | 0.9870855 | 0.9929748 | | TukeyHSD   |  |  |  |  |  | | --- | --- | --- | --- | --- | | groups | diff | lwr | upr | p-adj | | 1-0 | -0.44313859 | -0.8038432 | -0.08243395 | 0.0092339 | | 2-0 | -0.45547417 | -0.8277754 | -0.08317292 | 0.0096249 | | 3-0 | -0.79762781 | -1.3992393 | -0.19601635 | 0.0040872 | | 2-1 | -0.01233558 | -0.3811256 | 0.35645441 | 0.9997639 | | 3-1 | -0.35448922 | -0.9539341 | 0.24495568 | 0.4185372 | | 3-2 | -0.34215364 | -0.9486473 | 0.26434003 | 0.4608995 | | TukeyHSD   |  |  |  |  |  | | --- | --- | --- | --- | --- | | groups | diff | lwr | upr | p-adj | | 1-0 | -0.2379229 | -0.65362892 | 0.1777832 | 0.4480053 | | 2-0 | 0.1482092 | -0.28086174 | 0.5772802 | 0.8062547 | | 3-0 | 0.5837748 | -0.10957239 | 1.2771219 | 0.1314504 | | 2-1 | 0.3861321 | -0.03889222 | 0.8111564 | 0.0893808 | | 3-1 | 0.8216976 | 0.13084739 | 1.5125478 | 0.0126180 | | 3-2 | 0.4355655 | -0.26340826 | 1.1345394 | 0.3711799 | |
| 0: stroke, 1: statin, 2: control, 3: old. |

### CM2

| TG | Cholesterol | TG/CH |
| --- | --- | --- |
|  |  |  |
| TukeyHSD   |  |  |  |  |  | | --- | --- | --- | --- | --- | | groups | diff | lwr | upr | p-adj | | 1-0 | -0.35126118 | -0.6395021 | -0.06302027 | 0.0100013 | | 2-0 | -0.16347471 | -0.4609825 | 0.13403311 | 0.4842984 | | 3-0 | -0.21176669 | -0.6925175 | 0.26898408 | 0.6627548 | | 2-1 | 0.18778647 | -0.1069155 | 0.48248843 | 0.3510879 | | 3-1 | 0.13949449 | -0.3395250 | 0.61851395 | 0.8737718 | | 3-2 | -0.04829198 | -0.5329442 | 0.43636019 | 0.9938985 | | TukeyHSD   |  |  |  |  |  | | --- | --- | --- | --- | --- | | groups | diff | lwr | upr | p-adj | | 1-0 | -0.64318078 | -1.0221548 | -0.26420673 | 0.0001144 | | 2-0 | -0.42568289 | -0.8168409 | -0.03452487 | 0.0270787 | | 3-0 | -0.65641967 | -1.2885023 | -0.02433704 | 0.0385035 | | 2-1 | 0.21749790 | -0.1699710 | 0.60496682 | 0.4653710 | | 3-1 | -0.01323888 | -0.6430452 | 0.61656745 | 0.9999413 | | 3-2 | -0.23073678 | -0.8679489 | 0.40647534 | 0.7830123 | | TukeyHSD   |  |  |  |  |  | | --- | --- | --- | --- | --- | | groups | diff | lwr | upr | p-adj | | 1-0 | 0.29191961 | 0.032617404 | 0.5512218 | 0.0205494 | | 2-0 | 0.26220818 | -0.005430551 | 0.5298469 | 0.0571537 | | 3-0 | 0.44465298 | 0.012168447 | 0.8771375 | 0.0413494 | | 2-1 | -0.02971143 | -0.294826001 | 0.2354032 | 0.9913773 | | 3-1 | 0.15273337 | -0.278193671 | 0.5836604 | 0.7938303 | | 3-2 | 0.18244480 | -0.253549441 | 0.6184390 | 0.6979962 | |
| 0: stroke, 1: statin, 2: control, 3: old. |

### VLDL

| TG | Cholesterol | TG/CH |
| --- | --- | --- |
|  |  |  |
| TukeyHSD   |  |  |  |  |  | | --- | --- | --- | --- | --- | | groups | diff | lwr | upr | p-adj | | 1-0 | 0.03166595 | -0.1848442 | 0.2481761 | 0.9812622 | | 2-0 | -0.01136363 | -0.2348345 | 0.2121073 | 0.9991736 | | 3-0 | 0.14151319 | -0.2195994 | 0.5026257 | 0.7391815 | | 2-1 | -0.04302958 | -0.2643929 | 0.1783337 | 0.9578002 | | 3-1 | 0.10984724 | -0.2499649 | 0.4696593 | 0.8575162 | | 3-2 | 0.15287682 | -0.2111662 | 0.5169199 | 0.6956487 | | TukeyHSD   |  |  |  |  |  | | --- | --- | --- | --- | --- | | groups | diff | lwr | upr | p-adj | | 1-0 | -0.04682450 | -0.5901326 | 0.4964836 | 0.9960265 | | 2-0 | -0.09668225 | -0.6574577 | 0.4640932 | 0.9699495 | | 3-0 | -0.43110733 | -1.3372792 | 0.4750645 | 0.6051060 | | 2-1 | -0.04985776 | -0.6053444 | 0.5056289 | 0.9955191 | | 3-1 | -0.38428284 | -1.2871914 | 0.5186257 | 0.6866548 | | 3-2 | -0.33442508 | -1.2479507 | 0.5791006 | 0.7773221 | | TukeyHSD   |  |  |  |  |  | | --- | --- | --- | --- | --- | | groups | diff | lwr | upr | p-adj | | 1-0 | 0.078490447 | -0.4392168 | 0.5961977 | 0.9792122 | | 2-0 | 0.085318622 | -0.4490328 | 0.6196700 | 0.9758736 | | 3-0 | 0.572620523 | -0.2908521 | 1.4360932 | 0.3155233 | | 2-1 | 0.006828175 | -0.5224837 | 0.5361400 | 0.9999864 | | 3-1 | 0.494130076 | -0.3662330 | 1.3544931 | 0.4448841 | | 3-2 | 0.487301901 | -0.3831780 | 1.3577818 | 0.4677888 | |
| 0: stroke, 1: statin, 2: control, 3: old. |

### Lp(a)

| TG | Cholesterol | TG/CH |
| --- | --- | --- |
|  |  |  |
| TukeyHSD   |  |  |  |  |  | | --- | --- | --- | --- | --- | | groups | diff | lwr | upr | p-adj | | 1-0 | -0.08940393 | -0.2878600 | 0.10905211 | 0.6465131 | | 2-0 | -0.23411607 | -0.4389524 | -0.02927970 | 0.0180614 | | 3-0 | -0.26964165 | -0.6006422 | 0.06135888 | 0.1526177 | | 2-1 | -0.14471214 | -0.3476167 | 0.05819238 | 0.2530338 | | 3-1 | -0.18023772 | -0.5100462 | 0.14957080 | 0.4891051 | | 3-2 | -0.03552558 | -0.3692123 | 0.29816110 | 0.9925845 | | TukeyHSD   |  |  |  |  |  | | --- | --- | --- | --- | --- | | groups | diff | lwr | upr | p-adj | | 1-0 | -0.16726223 | -0.2823468 | -0.05217763 | 0.0012958 | | 2-0 | -0.26329709 | -0.3820816 | -0.14451254 | 0.0000003 | | 3-0 | -0.23517849 | -0.4271256 | -0.04323139 | 0.0094843 | | 2-1 | -0.09603486 | -0.2136991 | 0.02162941 | 0.1513526 | | 3-1 | -0.06791626 | -0.2591721 | 0.12333959 | 0.7928844 | | 3-2 | 0.02811860 | -0.1653862 | 0.22162340 | 0.9816098 | | TukeyHSD   |  |  |  |  |  | | --- | --- | --- | --- | --- | | groups | diff | lwr | upr | p-adj | | 1-0 | 0.07785830 | -0.08931416 | 0.2450308 | 0.6215757 | | 2-0 | 0.02918102 | -0.14336601 | 0.2017281 | 0.9715558 | | 3-0 | -0.03446316 | -0.31328648 | 0.2443602 | 0.9885124 | | 2-1 | -0.04867728 | -0.21959698 | 0.1222424 | 0.8808529 | | 3-1 | -0.11232145 | -0.39014066 | 0.1654978 | 0.7201495 | | 3-2 | -0.06364418 | -0.34473021 | 0.2174419 | 0.9355276 | |
| 0: stroke, 1: statin, 2: control, 3: old. |

### TR

| TG | Cholesterol | TG/CH |
| --- | --- | --- |
|  |  |  |
| TukeyHSD   |  |  |  |  |  | | --- | --- | --- | --- | --- | | groups | diff | lwr | upr | p-adj | | 1-0 | -0.04088578 | -0.30609983 | 0.2243283 | 0.9781889 | | 2-0 | 0.13247886 | -0.14126178 | 0.4062195 | 0.5915632 | | 3-0 | 0.42198409 | -0.02036068 | 0.8643288 | 0.0674488 | | 2-1 | 0.17336465 | -0.09779429 | 0.4445236 | 0.3480804 | | 3-1 | 0.46286987 | 0.02211810 | 0.9036216 | 0.0355012 | | 3-2 | 0.28950522 | -0.15642927 | 0.7354397 | 0.3343863 | | TukeyHSD   |  |  |  |  |  | | --- | --- | --- | --- | --- | | groups | diff | lwr | upr | p-adj | | 1-0 | -0.38297755 | -1.6483013 | 0.8823462 | 0.8606114 | | 2-0 | -0.04923864 | -1.3552423 | 1.2567651 | 0.9996622 | | 3-0 | -1.15107592 | -3.2614820 | 0.9593301 | 0.4908185 | | 2-1 | 0.33373891 | -0.9599476 | 1.6274254 | 0.9082053 | | 3-1 | -0.76809837 | -2.8709043 | 1.3347076 | 0.7784832 | | 3-2 | -1.10183728 | -3.2293697 | 1.0256952 | 0.5356392 | | TukeyHSD   |  |  |  |  |  | | --- | --- | --- | --- | --- | | groups | diff | lwr | upr | p-adj | | 1-0 | 0.3420918 | -0.8738824 | 1.558066 | 0.8845935 | | 2-0 | 0.1817175 | -1.0733500 | 1.436785 | 0.9818014 | | 3-0 | 1.5730600 | -0.4550369 | 3.601157 | 0.1870549 | | 2-1 | -0.1603743 | -1.4036050 | 1.082856 | 0.9869867 | | 3-1 | 1.2309682 | -0.7898250 | 3.251761 | 0.3917229 | | 3-2 | 1.3913425 | -0.6532129 | 3.435898 | 0.2929195 | |
| 0: stroke, 1: statin, 2: control, 3: old. |

### LDL1

| TG | Cholesterol | TG/CH |
| --- | --- | --- |
|  |  |  |
| TukeyHSD   |  |  |  |  |  | | --- | --- | --- | --- | --- | | groups | diff | lwr | upr | p-adj | | 1-0 | -0.006858426 | -0.116622339 | 0.10290549 | 0.9984720 | | 2-0 | -0.099270200 | -0.212563007 | 0.01402261 | 0.1081930 | | 3-0 | 0.091836667 | -0.091236186 | 0.27490952 | 0.5623834 | | 2-1 | -0.092411774 | -0.204636093 | 0.01981255 | 0.1455195 | | 3-1 | 0.098695093 | -0.083718468 | 0.28110865 | 0.4978876 | | 3-2 | 0.191106867 | 0.006548337 | 0.37566540 | 0.0393150 | | TukeyHSD   |  |  |  |  |  | | --- | --- | --- | --- | --- | | groups | diff | lwr | upr | p-adj | | 1-0 | -0.060653111 | -0.12115671 | -0.0001495081 | 0.0491831 | | 2-0 | -0.013507641 | -0.07595643 | 0.0489411446 | 0.9431594 | | 3-0 | -0.007970661 | -0.10888331 | 0.0929419892 | 0.9969319 | | 2-1 | 0.047145470 | -0.01471435 | 0.1090052885 | 0.2001798 | | 3-1 | 0.052682451 | -0.04786679 | 0.1532316883 | 0.5257234 | | 3-2 | 0.005536980 | -0.09619460 | 0.1072685586 | 0.9989877 | | TukeyHSD   |  |  |  |  |  | | --- | --- | --- | --- | --- | | groups | diff | lwr | upr | p-adj | | 1-0 | 0.05379469 | -0.062118638 | 0.16970801 | 0.6242724 | | 2-0 | -0.08576256 | -0.205402480 | 0.03387736 | 0.2488219 | | 3-0 | 0.09980733 | -0.093521995 | 0.29313665 | 0.5383291 | | 2-1 | -0.13955724 | -0.258068816 | -0.02104567 | 0.0138405 | | 3-1 | 0.04601264 | -0.146620452 | 0.23864574 | 0.9253936 | | 3-2 | 0.18556989 | -0.009328346 | 0.38046812 | 0.0682376 | |
| 0: stroke, 1: statin, 2: control, 3: old. |

### LAC1

| TG | Cholesterol | TG/CH |
| --- | --- | --- |
|  |  |  |
| TukeyHSD   |  |  |  |  |  | | --- | --- | --- | --- | --- | | groups | diff | lwr | upr | p-adj | | 1-0 | -0.07654825 | -0.4669246 | 0.31382805 | 0.9567534 | | 2-0 | -0.36385807 | -0.7667849 | 0.03906878 | 0.0923626 | | 3-0 | 0.04154561 | -0.6095546 | 0.69264579 | 0.9983734 | | 2-1 | -0.28730982 | -0.6864366 | 0.11181694 | 0.2453728 | | 3-1 | 0.11809386 | -0.5306615 | 0.76684927 | 0.9649356 | | 3-2 | 0.40540368 | -0.2509803 | 1.06178768 | 0.3792158 | | TukeyHSD   |  |  |  |  |  | | --- | --- | --- | --- | --- | | groups | diff | lwr | upr | p-adj | | 1-0 | -0.18286494 | -0.7183304 | 0.3526005 | 0.8115515 | | 2-0 | -0.23625637 | -0.7889369 | 0.3164242 | 0.6836754 | | 3-0 | 0.19525020 | -0.6978410 | 1.0883414 | 0.9414566 | | 2-1 | -0.05339143 | -0.6008596 | 0.4940767 | 0.9942733 | | 3-1 | 0.37811514 | -0.5117598 | 1.2679901 | 0.6877653 | | 3-2 | 0.43150657 | -0.4688323 | 1.3318454 | 0.5992940 | | TukeyHSD   |  |  |  |  |  | | --- | --- | --- | --- | --- | | groups | diff | lwr | upr | p-adj | | 1-0 | 0.10631669 | -0.2205362 | 0.4331696 | 0.8328605 | | 2-0 | -0.12760170 | -0.4649629 | 0.2097595 | 0.7596116 | | 3-0 | -0.15370459 | -0.6988555 | 0.3914463 | 0.8839315 | | 2-1 | -0.23391838 | -0.5680978 | 0.1002611 | 0.2687348 | | 3-1 | -0.26002128 | -0.8032089 | 0.2831664 | 0.6002441 | | 3-2 | -0.02610289 | -0.5756778 | 0.5234720 | 0.9993260 | |
| 0: stroke, 1: statin, 2: control, 3: old. |

### LDL2+LAC2

| TG | Cholesterol | TG/CH |
| --- | --- | --- |
|  |  |  |
| TukeyHSD   |  |  |  |  |  | | --- | --- | --- | --- | --- | | groups | diff | lwr | upr | p-adj | | 1-0 | 0.05738289 | -0.05928458 | 0.1740504 | 0.5785368 | | 2-0 | 0.08063069 | -0.03978763 | 0.2010490 | 0.3070361 | | 3-0 | 0.11437133 | -0.08021583 | 0.3089585 | 0.4240512 | | 2-1 | 0.02324780 | -0.09603483 | 0.1425304 | 0.9574871 | | 3-1 | 0.05698844 | -0.13689796 | 0.2508748 | 0.8707084 | | 3-2 | 0.03374064 | -0.16242563 | 0.2299069 | 0.9701517 | | TukeyHSD   |  |  |  |  |  | | --- | --- | --- | --- | --- | | groups | diff | lwr | upr | p-adj | | 1-0 | 0.030526442 | -0.0269986 | 0.0880514 | 0.514756 | | 2-0 | 0.122289460 | 0.06291500 | 0.1816639 | 0.000001 | | 3-0 | 0.039432320 | -0.0565124 | 0.1353770 | 0.709733 | | 2-1 | 0.091763018 | 0.03294853 | 0.1505775 | 0.000463 | | 3-1 | 0.008905878 | -0.0866937 | 0.1045051 | 0.994997 | | 3-2 | -0.08285714 | -0.1795805 | 0.0138662 | 0.121057 | | TukeyHSD   |  |  |  |  |  | | --- | --- | --- | --- | --- | | groups | diff | lwr | upr | p-adj | | 1-0 | 0.02685645 | -0.1044386 | 0.15815151 | 0.9513270 | | 2-0 | -0.04165877 | -0.1771750 | 0.09385741 | 0.8549901 | | 3-0 | 0.07493901 | -0.1440452 | 0.29392320 | 0.8106009 | | 2-1 | -0.06851522 | -0.2027533 | 0.06572288 | 0.5479522 | | 3-1 | 0.04808256 | -0.1701130 | 0.26627813 | 0.9401611 | | 3-2 | 0.11659778 | -0.1041635 | 0.33735907 | 0.5188353 | |
| 0: stroke, 1: statin, 2: control, 3: old. |

### mHDL

| TG | Cholesterol | TG/CH |
| --- | --- | --- |
|  |  |  |
| TukeyHSD   |  |  |  |  |  | | --- | --- | --- | --- | --- | | groups | diff | lwr | upr | p-adj | | 1-0 | 0.21225540 | -0.4984211 | 0.9229319 | 0.8652813 | | 2-0 | -0.05187019 | -0.7853948 | 0.6816544 | 0.9977914 | | 3-0 | 0.22801638 | -0.9573055 | 1.4133383 | 0.9590202 | | 2-1 | -0.26412558 | -0.9907322 | 0.4624810 | 0.7810131 | | 3-1 | 0.01576098 | -1.1652923 | 1.1968142 | 0.9999850 | | 3-2 | 0.27988657 | -0.9150545 | 1.4748276 | 0.9292673 | | TukeyHSD   |  |  |  |  |  | | --- | --- | --- | --- | --- | | groups | diff | lwr | upr | p-adj | | 1-0 | -0.17429630 | -1.0671584 | 0.7185658 | 0.9572945 | | 2-0 | -0.13579989 | -1.0573674 | 0.7857676 | 0.9808515 | | 3-0 | 0.09613465 | -1.3930507 | 1.5853200 | 0.9983161 | | 2-1 | 0.03849641 | -0.8743796 | 0.9513724 | 0.9995277 | | 3-1 | 0.27043096 | -1.2133915 | 1.7542534 | 0.9648147 | | 3-2 | 0.23193455 | -1.2693359 | 1.7332050 | 0.9780539 | | TukeyHSD   |  |  |  |  |  | | --- | --- | --- | --- | --- | | groups | diff | lwr | upr | p-adj | | 1-0 | 0.38655170 | -0.4470833 | 1.2201867 | 0.6249287 | | 2-0 | 0.08392971 | -0.7765065 | 0.9443659 | 0.9942700 | | 3-0 | 0.13188173 | -1.2585200 | 1.5222834 | 0.9947244 | | 2-1 | -0.30262199 | -1.1549432 | 0.5496993 | 0.7929552 | | 3-1 | -0.25466997 | -1.6400645 | 1.1307245 | 0.9639489 | | 3-2 | 0.04795202 | -1.3537331 | 1.4496371 | 0.9997474 | |
| 0: stroke, 1: statin, 2: control, 3: old. |

### HDL1

| TG | Cholesterol | TG/CH |
| --- | --- | --- |
|  |  |  |
| TukeyHSD   |  |  |  |  |  | | --- | --- | --- | --- | --- | | groups | diff | lwr | upr | p-adj | | 1-0 | -0.120801405 | -0.25123361 | 0.009630805 | 0.0802563 | | 2-0 | 0.210913357 | 0.07628777 | 0.345538944 | 0.0004349 | | 3-0 | 0.005797464 | -0.21174759 | 0.223342522 | 0.9998805 | | 2-1 | 0.331714762 | 0.19835886 | 0.465070667 | 0.0000000 | | 3-1 | 0.126598868 | -0.09016275 | 0.343360491 | 0.4297773 | | 3-2 | -0.205115893 | -0.42442638 | 0.014194590 | 0.0758557 | | TukeyHSD   |  |  |  |  |  | | --- | --- | --- | --- | --- | | groups | diff | lwr | upr | p-adj | | 1-0 | 0.7441361 | 0.09761364 | 1.3906585 | 0.0169904 | | 2-0 | 1.6164066 | 0.94909858 | 2.2837147 | 0.0000000 | | 3-0 | 1.1949565 | 0.11663573 | 2.2732773 | 0.0234185 | | 2-1 | 0.8722705 | 0.21125601 | 1.5332851 | 0.0043130 | | 3-1 | 0.4508204 | -0.62361705 | 1.5252579 | 0.6962091 | | 3-2 | -0.4214501 | -1.50852172 | 0.6656215 | 0.7455088 | | TukeyHSD   |  |  |  |  |  | | --- | --- | --- | --- | --- | | groups | diff | lwr | upr | p-adj | | 1-0 | -0.8649375 | -1.5376399 | -0.19223506 | 0.0057547 | | 2-0 | -1.4054933 | -2.0998230 | -0.71116355 | 0.0000029 | | 3-0 | -1.1891591 | -2.3111449 | -0.06717323 | 0.0331671 | | 2-1 | -0.5405558 | -1.2283371 | 0.14722558 | 0.1774533 | | 3-1 | -0.3242216 | -1.4421668 | 0.79372371 | 0.8751045 | | 3-2 | 0.2163342 | -0.9147568 | 1.34742521 | 0.9596793 | |
| 0: stroke, 1: statin, 2: control, 3: old. |

### HDL2

| TG | Cholesterol | TG/CH |
| --- | --- | --- |
|  |  |  |
| TukeyHSD   |  |  |  |  |  | | --- | --- | --- | --- | --- | | groups | diff | lwr | upr | p-adj | | 1-0 | 0.01578431 | -0.4896155 | 0.5211842 | 0.9998077 | | 2-0 | 0.20819169 | -0.3134567 | 0.7298401 | 0.7281584 | | 3-0 | -1.04845570 | -1.8914012 | -0.2055102 | 0.0081549 | | 2-1 | 0.19240737 | -0.3243212 | 0.7091360 | 0.7682376 | | 3-1 | -1.06424001 | -1.9041498 | -0.2243302 | 0.0067290 | | 3-2 | -1.25664738 | -2.1064335 | -0.4068612 | 0.0010203 | | TukeyHSD   |  |  |  |  |  | | --- | --- | --- | --- | --- | | groups | diff | lwr | upr | p-adj | | 1-0 | 0.2658196 | -0.03507669 | 0.5667159 | 0.1037451 | | 2-0 | 0.3705950 | 0.06002492 | 0.6811651 | 0.0122390 | | 3-0 | 0.1301791 | -0.37167933 | 0.6320375 | 0.9068519 | | 2-1 | 0.1047754 | -0.20286564 | 0.4124164 | 0.8128013 | | 3-1 | -0.1356405 | -0.63569162 | 0.3644106 | 0.8951279 | | 3-2 | -0.2404159 | -0.74634701 | 0.2655152 | 0.6060134 | | TukeyHSD   |  |  |  |  |  | | --- | --- | --- | --- | --- | | groups | diff | lwr | upr | p-adj | | 1-0 | -0.25003529 | -0.7833813 | 0.28331075 | 0.6165323 | | 2-0 | -0.16240330 | -0.7128963 | 0.38808974 | 0.8694731 | | 3-0 | -1.17863478 | -2.0681911 | -0.28907846 | 0.0041174 | | 2-1 | 0.08763199 | -0.4576692 | 0.63293322 | 0.9754216 | | 3-1 | -0.92859949 | -1.8149523 | -0.04224669 | 0.0361310 | | 3-2 | -1.01623148 | -1.9130067 | -0.11945622 | 0.0194384 | |
| 0: stroke, 1: statin, 2: control, 3: old. |

### Free Glycerol

| Glycerol |
| --- |
|  |
| TukeyHSD   |  |  |  |  |  | | --- | --- | --- | --- | --- | | groups | diff | lwr | upr | p-adj | | 1-0 | -0.10659677 | -0.2374917 | 0.02429814 | 0.1528280 | | 2-0 | -0.19385482 | -0.3289580 | -0.05875165 | 0.0015393 | | 3-0 | -0.42482680 | -0.6431436 | -0.20651002 | 0.0000073 | | 2-1 | -0.08725804 | -0.2210870 | 0.04657093 | 0.3305502 | | 3-1 | -0.31823003 | -0.5357606 | -0.10069946 | 0.0011850 | | 3-2 | -0.23097198 | -0.4510605 | -0.01088351 | 0.0356832 | |
| 0: stroke, 1: statin, 2: control, 3: old. |

| Glycerol to CM1 ratio | Glycerol to TR | Glycerol to LDL1 | Glycerol to LDL2+LAC2 |
| --- | --- | --- | --- |
|  |  |  |  |
|  |
| Correlation to glycerol |
| |  |  | | --- | --- | | class | Pearson's r | | CM1 | -0.167529150865999 | | CM2 | -0.0841126067569958 | | VLDL | -0.0814365817125813 | | Lp(a) | -0.216746854774793 | | TR | -0.207463680240653 | | LDL1 | 0.0162021823361841 | | LAC1 | -0.0732322047123116 | | LDL2 | -0.175758937353437 | | LAC2 | -0.086804455339854 | | mHDL | -0.183683185654165 | | HDL1 | -0.0980736922032977 | | HDL2 | 0.0732104080735861 | |

  
  

| Glycerol and age | Glycerol and age of 65 |
| --- | --- |
|  |  |
|  | TukeyHSD   |  |  |  |  |  | | --- | --- | --- | --- | --- | | groups | diff | lwr | upr | p-adj | | 1-0 | 0.13071375 | -0.03943972 | 0.300867226 | 0.1941940 | | 2-0 | -0.23097198 | -0.45425102 | -0.007692946 | 0.0395941 | | 3-0 | 0.14211208 | 0.01755029 | 0.266673867 | 0.0183433 | | 2-1 | -0.36168574 | -0.60503819 | -0.118333279 | 0.0009503 | | 3-1 | 0.01139833 | -0.14634364 | 0.169140295 | 0.9976448 | | 3-2 | 0.37308406 | 0.15911244 | 0.587055680 | 0.0000697 | |
|  | 0 and 1: under 65, 2 and 3: over 65, 0 and 2: control, 1 and 3: stroke. |

  
  


## S6 Fig: Current methods

| Total TG | Total Cholesterol |
| --- | --- |
|  |  |
| TukeyHSD   |  |  |  |  |  | | --- | --- | --- | --- | --- | | groups | diff | lwr | upr | p-adj | | 1-0 | -0.15526944 | -0.6214609 | 0.3109220 | 0.8227774 | | 2-0 | -0.37328102 | -0.8544604 | 0.1078984 | 0.1869324 | | 3-0 | -0.07210346 | -0.8496540 | 0.7054471 | 0.9950652 | | 2-1 | -0.21801158 | -0.6946529 | 0.2586297 | 0.6352573 | | 3-1 | 0.08316598 | -0.6915845 | 0.8579164 | 0.9924024 | | 3-2 | 0.30117756 | -0.4826830 | 1.0850381 | 0.7507130 | | TukeyHSD   |  |  |  |  |  | | --- | --- | --- | --- | --- | | groups | diff | lwr | upr | p-adj | | 1-0 | -0.8275592 | -1.5841213 | -0.07099712 | 0.0260051 | | 2-0 | 0.1047257 | -0.6761597 | 0.88561117 | 0.9854271 | | 3-0 | -0.5635101 | -1.8253636 | 0.69834338 | 0.6528974 | | 2-1 | 0.9322849 | 0.1587642 | 1.70580568 | 0.0111315 | | 3-1 | 0.2640491 | -0.9932602 | 1.52135832 | 0.9476227 | | 3-2 | -0.6682359 | -1.9403296 | 0.60385787 | 0.5235040 | |

0: stroke, 1: statin, 2: control, 3: old.

| LDL | HDL |
| --- | --- |
|  |  |
| TukeyHSD   |  |  |  |  |  | | --- | --- | --- | --- | --- | | groups | diff | lwr | upr | p-adj | | 1-0 | -1.0754578 | -1.790797962 | -0.3601176 | 0.0008065 | | 2-0 | -0.3540259 | -1.092364172 | 0.3843124 | 0.5989332 | | 3-0 | -0.7426145 | -1.935714921 | 0.4504859 | 0.3722322 | | 2-1 | 0.7214319 | -0.009942977 | 1.4528067 | 0.0547032 | | 3-1 | 0.3328433 | -0.855960487 | 1.5216470 | 0.8860344 | | 3-2 | -0.3885886 | -1.591371295 | 0.8141941 | 0.8356614 | | TukeyHSD   |  |  |  |  |  | | --- | --- | --- | --- | --- | | groups | diff | lwr | upr | p-adj | | 1-0 | -0.15526944 | -0.6214609 | 0.3109220 | 0.8227774 | | 2-0 | -0.37328102 | -0.8544604 | 0.1078984 | 0.1869324 | | 3-0 | -0.07210346 | -0.8496540 | 0.7054471 | 0.9950652 | | 2-1 | -0.21801158 | -0.6946529 | 0.2586297 | 0.6352573 | | 3-1 | 0.08316598 | -0.6915845 | 0.8579164 | 0.9924024 | | 3-2 | 0.30117756 | -0.4826830 | 1.0850381 | 0.7507130 | |

0: stroke, 1: statin, 2: control, 3: old.

  
  

index
