# Supplementary material for: Distinctive features of lipoprotein profiles in stroke patients
Source: PLoS One. 2023 Apr 5;18(4):e0283855. doi: 10.1371/journal.pone.0283855 (PMC10075468; doi:10.1371/journal.pone.0283855)
Supplement: S1 File — (ZIP) [file pone.0283855.s001.zip › supplement/ind/PC.htm]

Support


## Supporting information

for **Distinctive features of lipoprotein profiles in stroke patients**  
  
Jump to S3 and  S4 .

## S3 Fig: TG and Cholesterol

### PC\_samples

| PC1, 2 | PC3, 4 | PC5, 6 |
| --- | --- | --- |
|  |  |  |
|  |  |  |
| PC7, 8 | PC9, 10 |  |
|  |  |  |
|  |  |

### PC\_items

| PC1, 2 | PC3, 4 | PC5, 6 |
| --- | --- | --- |
|  |  |  |
|  |  |
| PC7, 8 | PC9, 10 |  |
|  |  |
|  |  |


## S4 Fig: Current Methods

  

### PC\_samples

| PC1, 2 | PC3, 4 |
| --- | --- |
|  |  |

  

### PC\_items

|  |  |
| --- | --- |
|  |  |

  
  

index
