# Supplementary material for: Distinctive features of lipoprotein profiles in stroke patients
Source: PLoS One. 2023 Apr 5;18(4):e0283855. doi: 10.1371/journal.pone.0283855 (PMC10075468; doi:10.1371/journal.pone.0283855)
Supplement: S1 File — (ZIP) [file pone.0283855.s001.zip › supplement/index.htm]

Support


## Supporting information

for **Distinctive features of lipoprotein profiles in stroke patients**

  

## S1 Fig: Age of sample

## S2 Fig: Contribution of PCA

## S3 Fig: TG and Cholesterol

## S4 Fig: Current Methods

## S5 Fig: Box plots / Classes

## S6 Fig: Box plots / Current Methods

## S1 Table: symptom records

## S2 Table: fitting data

## S3 Table: PC for the samples

## S4 Table: PC for the items
