# Supplementary material for: Distinctive features of lipoprotein profiles in stroke patients
Source: PLoS One. 2023 Apr 5;18(4):e0283855. doi: 10.1371/journal.pone.0283855 (PMC10075468; doi:10.1371/journal.pone.0283855)
Supplement: S1 File — (ZIP) [file pone.0283855.s001.zip › supplement/pages/S2Fig.htm]

Support


Click on the image to enlarge

## S2 Fig: Contribution of PCA

| TG and Cholesterol | Current Methods |
| --- | --- |
|  |  |

index page
