# Supplementary material for: Distinctive features of lipoprotein profiles in stroke patients
Source: PLoS One. 2023 Apr 5;18(4):e0283855. doi: 10.1371/journal.pone.0283855 (PMC10075468; doi:10.1371/journal.pone.0283855)
Supplement: S1 File — (ZIP) [file pone.0283855.s001.zip › supplement/pages/S6Fig.htm]

Support


Click on the image to enlarge

## S6 Fig: Current methods

| Total TG | Total Cholesterol |
| --- | --- |
|  |  |
| TukeyHSD   |  |  |  |  |  | | --- | --- | --- | --- | --- | | groups | diff | lwr | upr | p-adj | | 1-0 | -0.15526944 | -0.6214609 | 0.3109220 | 0.8227774 | | 2-0 | -0.37328102 | -0.8544604 | 0.1078984 | 0.1869324 | | 3-0 | -0.07210346 | -0.8496540 | 0.7054471 | 0.9950652 | | 2-1 | -0.21801158 | -0.6946529 | 0.2586297 | 0.6352573 | | 3-1 | 0.08316598 | -0.6915845 | 0.8579164 | 0.9924024 | | 3-2 | 0.30117756 | -0.4826830 | 1.0850381 | 0.7507130 | | TukeyHSD   |  |  |  |  |  | | --- | --- | --- | --- | --- | | groups | diff | lwr | upr | p-adj | | 1-0 | -0.8275592 | -1.5841213 | -0.07099712 | 0.0260051 | | 2-0 | 0.1047257 | -0.6761597 | 0.88561117 | 0.9854271 | | 3-0 | -0.5635101 | -1.8253636 | 0.69834338 | 0.6528974 | | 2-1 | 0.9322849 | 0.1587642 | 1.70580568 | 0.0111315 | | 3-1 | 0.2640491 | -0.9932602 | 1.52135832 | 0.9476227 | | 3-2 | -0.6682359 | -1.9403296 | 0.60385787 | 0.5235040 | |

0: stroke, 1: statin, 2: control, 3: old.

| LDL | HDL |
| --- | --- |
|  |  |
| TukeyHSD   |  |  |  |  |  | | --- | --- | --- | --- | --- | | groups | diff | lwr | upr | p-adj | | 1-0 | -1.0754578 | -1.790797962 | -0.3601176 | 0.0008065 | | 2-0 | -0.3540259 | -1.092364172 | 0.3843124 | 0.5989332 | | 3-0 | -0.7426145 | -1.935714921 | 0.4504859 | 0.3722322 | | 2-1 | 0.7214319 | -0.009942977 | 1.4528067 | 0.0547032 | | 3-1 | 0.3328433 | -0.855960487 | 1.5216470 | 0.8860344 | | 3-2 | -0.3885886 | -1.591371295 | 0.8141941 | 0.8356614 | | TukeyHSD   |  |  |  |  |  | | --- | --- | --- | --- | --- | | groups | diff | lwr | upr | p-adj | | 1-0 | -0.15526944 | -0.6214609 | 0.3109220 | 0.8227774 | | 2-0 | -0.37328102 | -0.8544604 | 0.1078984 | 0.1869324 | | 3-0 | -0.07210346 | -0.8496540 | 0.7054471 | 0.9950652 | | 2-1 | -0.21801158 | -0.6946529 | 0.2586297 | 0.6352573 | | 3-1 | 0.08316598 | -0.6915845 | 0.8579164 | 0.9924024 | | 3-2 | 0.30117756 | -0.4826830 | 1.0850381 | 0.7507130 | |

0: stroke, 1: statin, 2: control, 3: old.

  
  

index
