# Supplementary figures and images for: Distinctive features of lipoprotein profiles in stroke patients
Source: PLoS One. 2023 Apr 5;18(4):e0283855. doi: 10.1371/journal.pone.0283855 (PMC10075468; doi:10.1371/journal.pone.0283855)

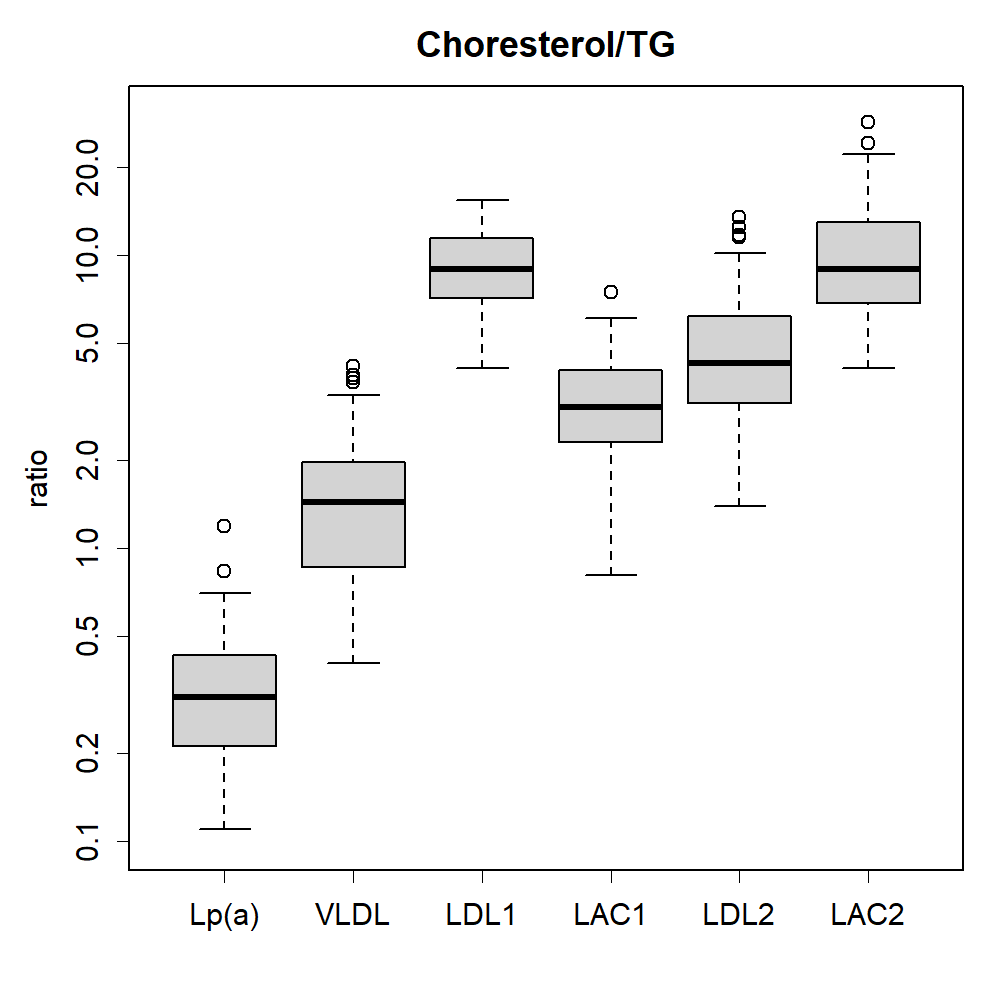

Supplement: S1 File — (ZIP) [file pone.0283855.s001.zip › supplement/CH/CH_TGbox.png]

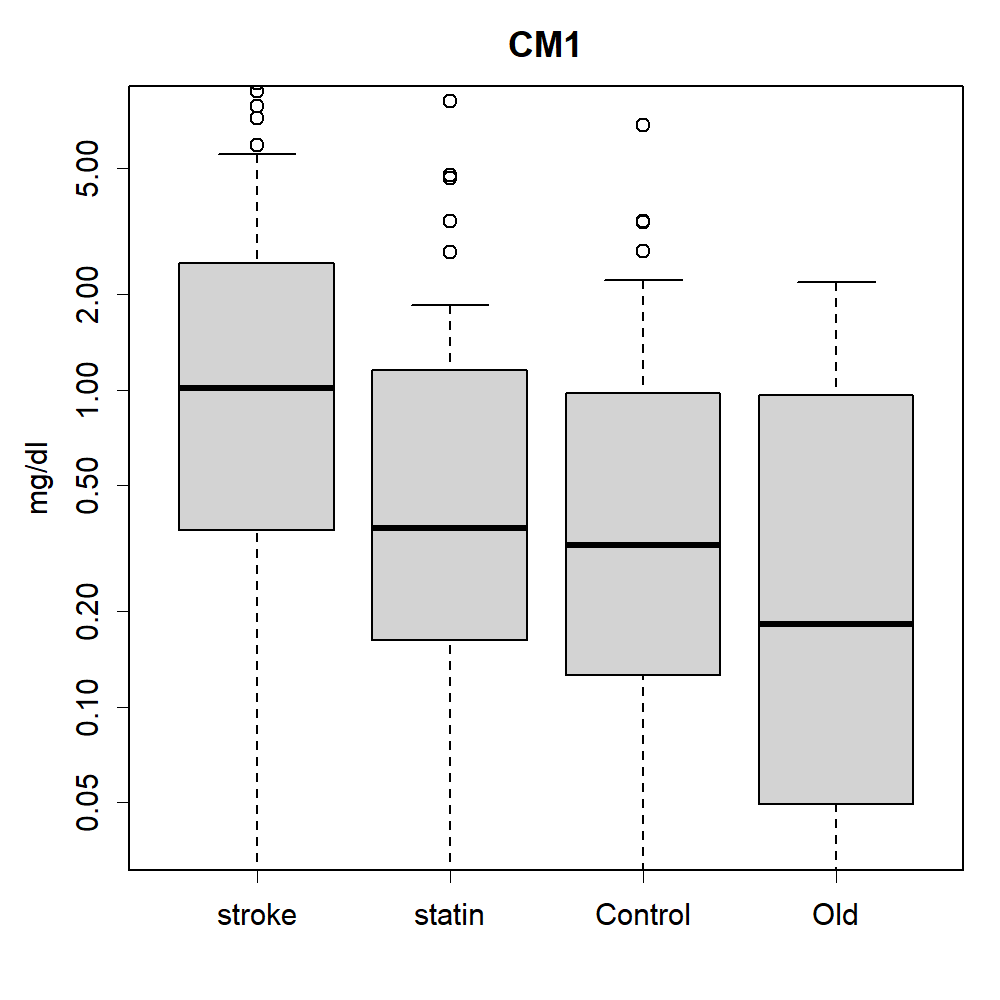

Supplement: S1 File — (ZIP) [file pone.0283855.s001.zip › supplement/CH/CM1.png]

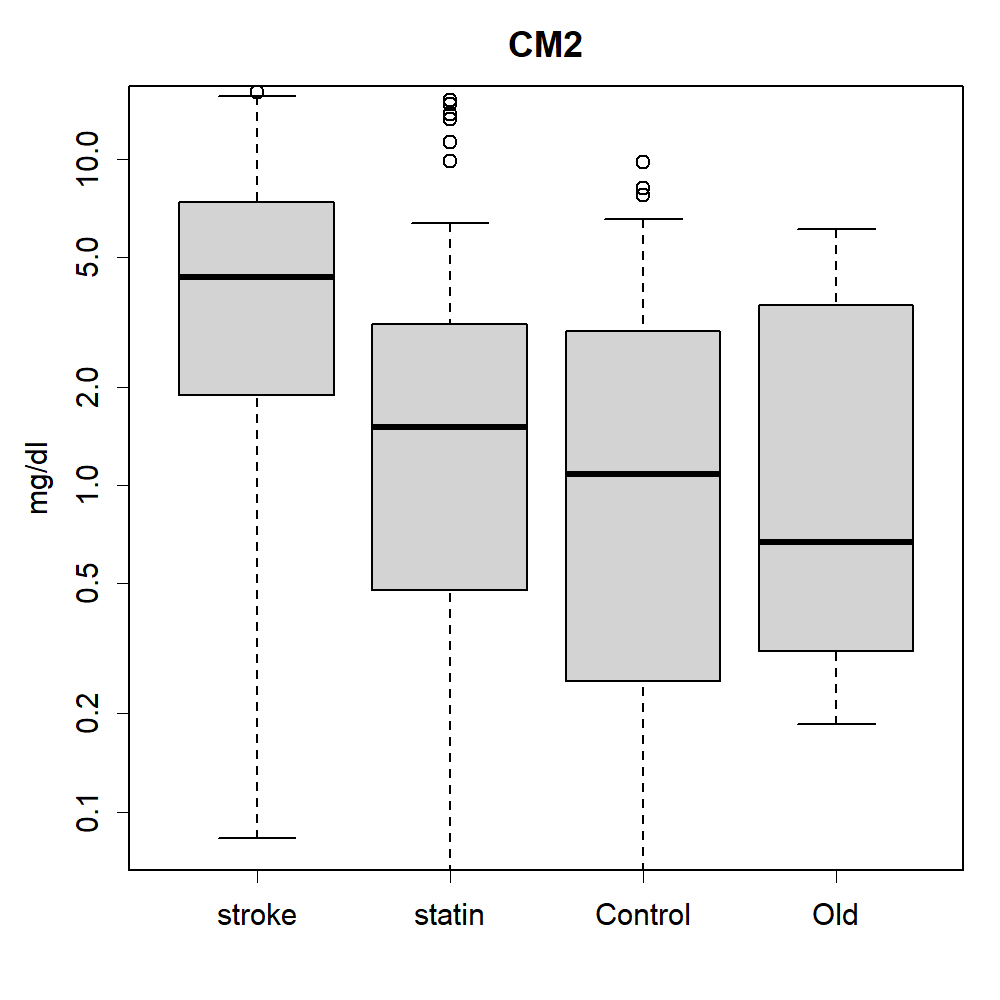

Supplement: S1 File — (ZIP) [file pone.0283855.s001.zip › supplement/CH/CM2.png]

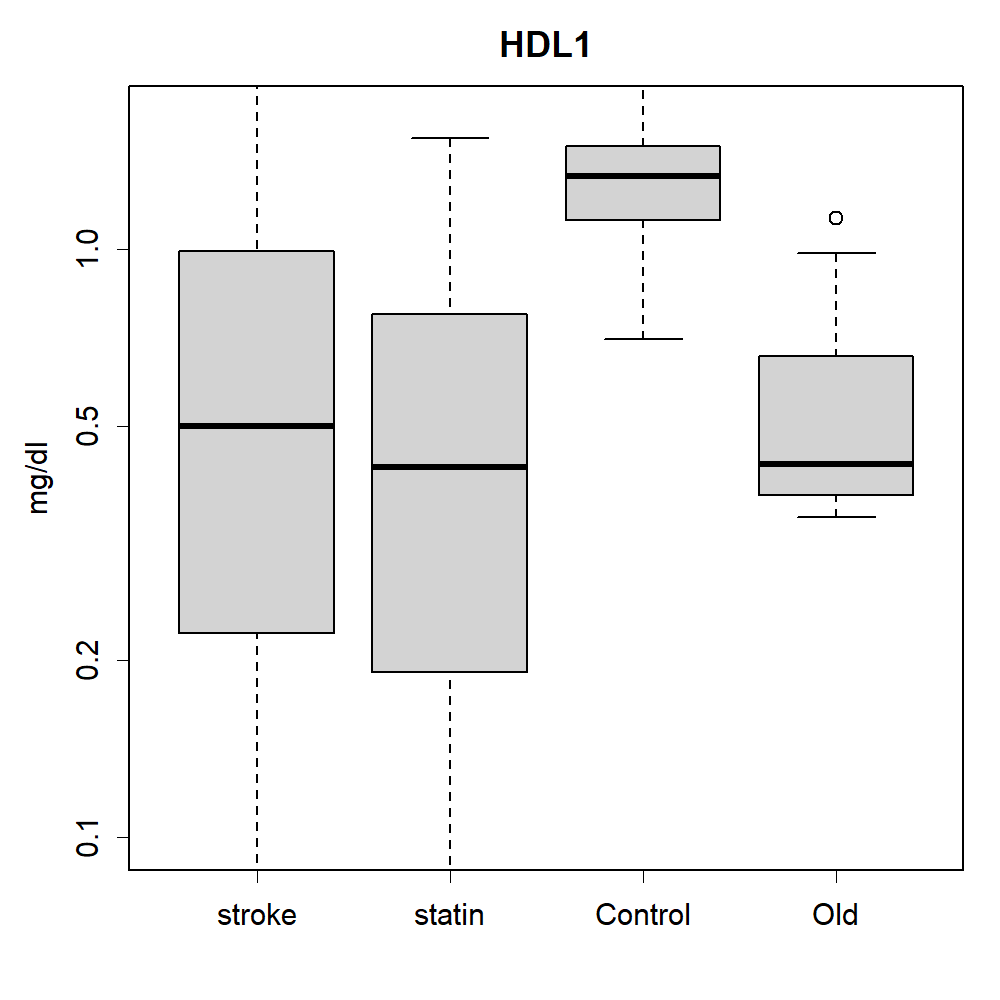

Supplement: S1 File — (ZIP) [file pone.0283855.s001.zip › supplement/CH/HDL1.png]

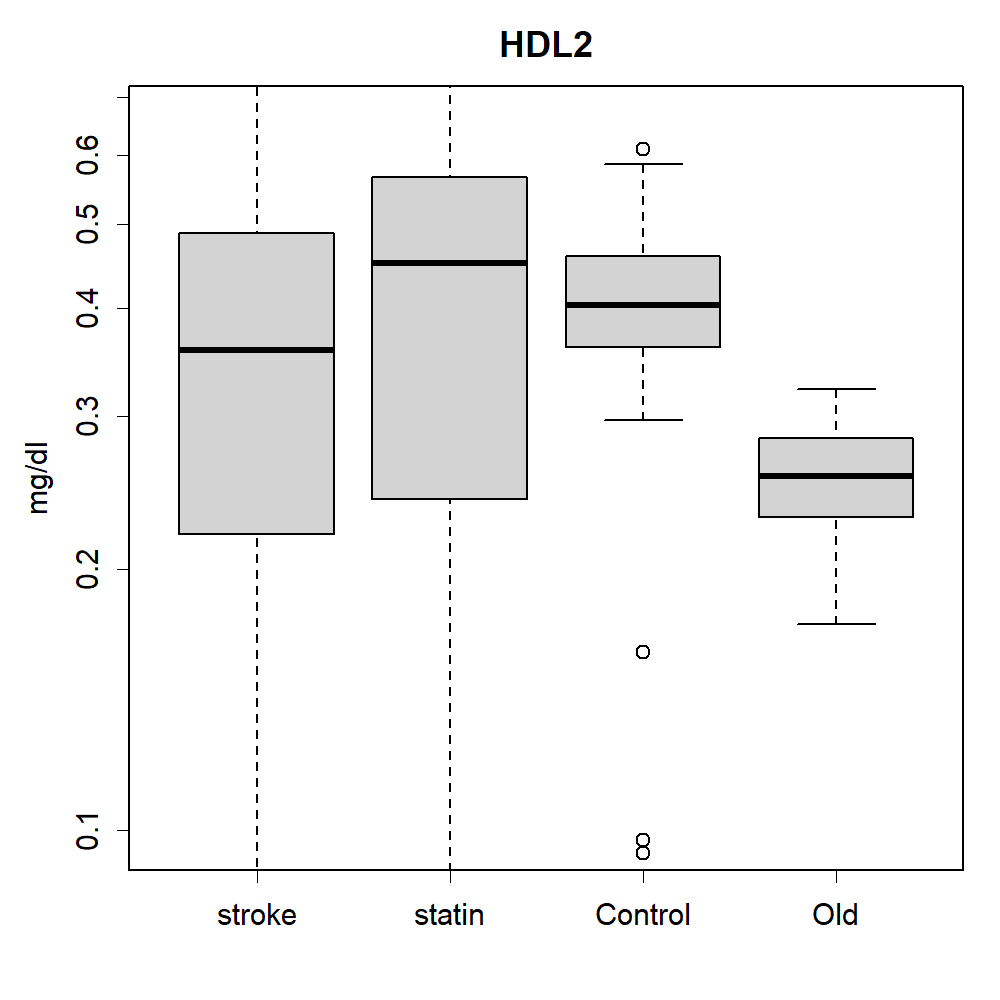

Supplement: S1 File — (ZIP) [file pone.0283855.s001.zip › supplement/CH/HDL2.png]

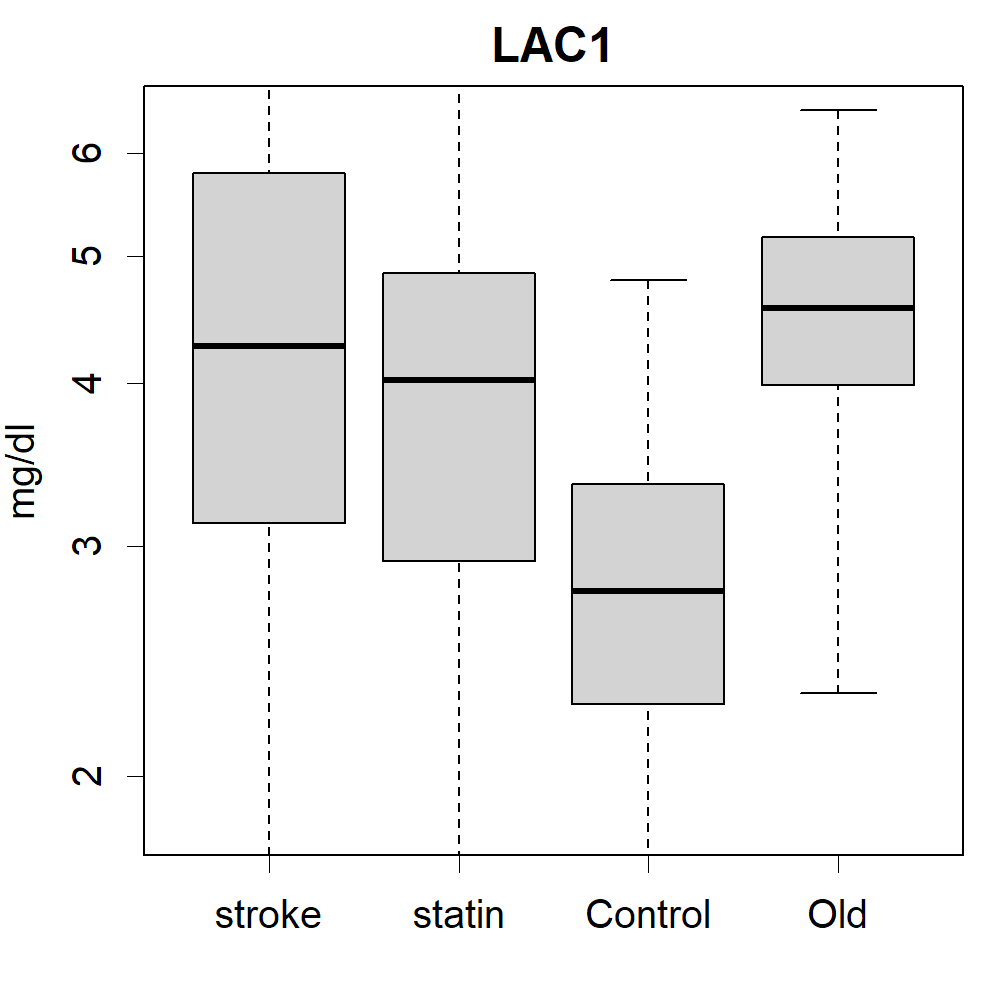

Supplement: S1 File — (ZIP) [file pone.0283855.s001.zip › supplement/CH/LAC1.png]

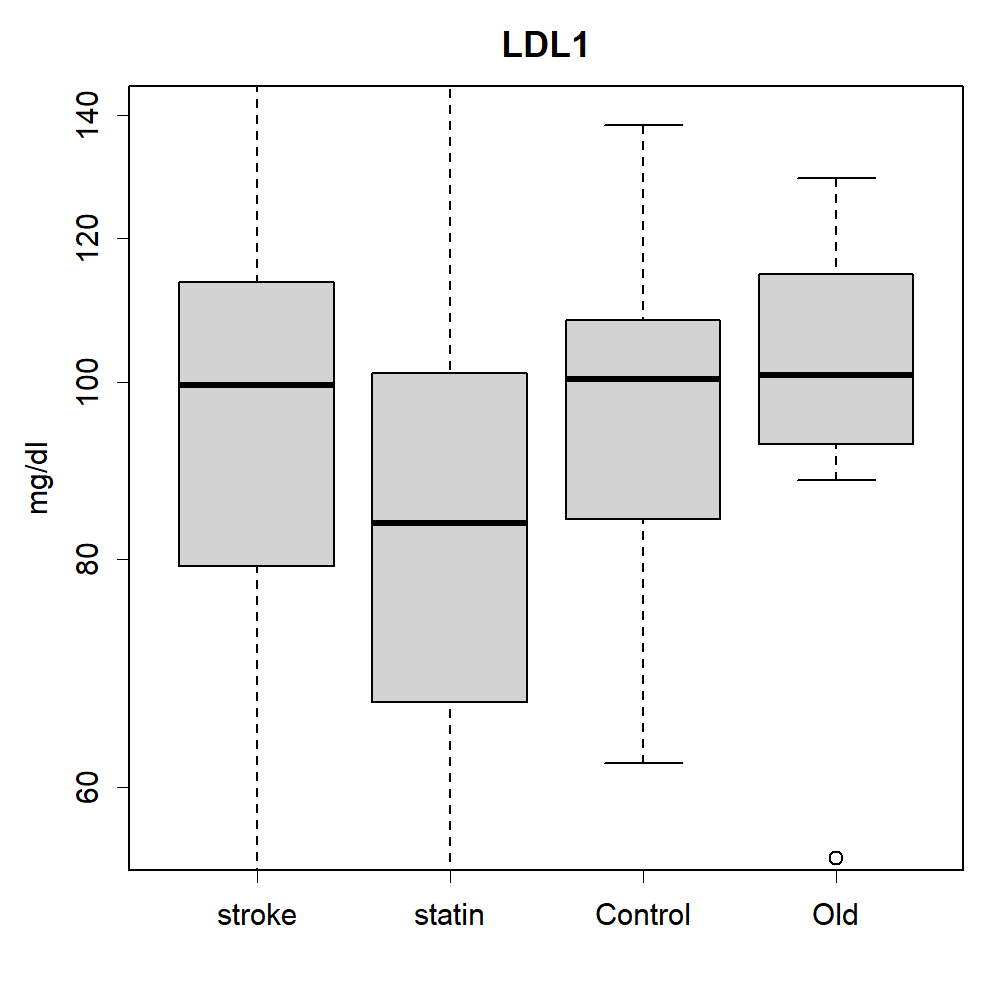

Supplement: S1 File — (ZIP) [file pone.0283855.s001.zip › supplement/CH/LDL1.png]

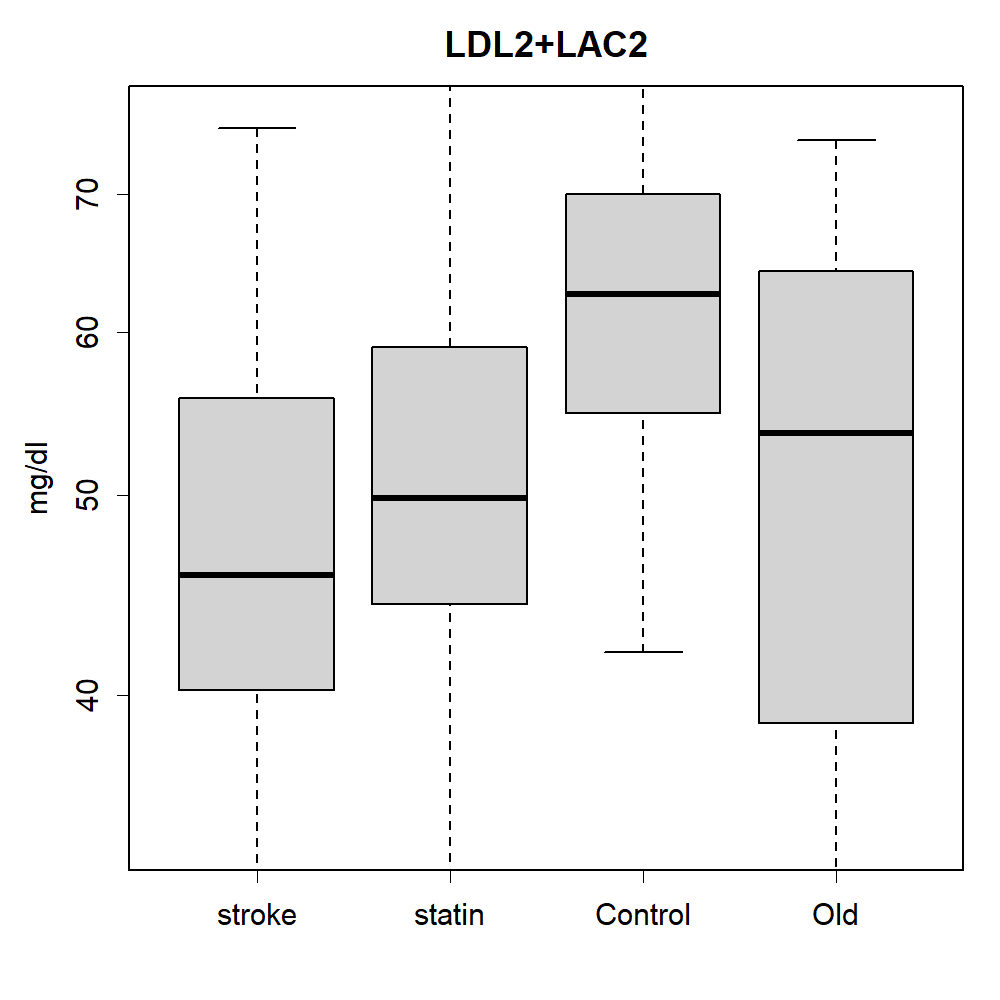

Supplement: S1 File — (ZIP) [file pone.0283855.s001.zip › supplement/CH/LDL2+LAC2.png]

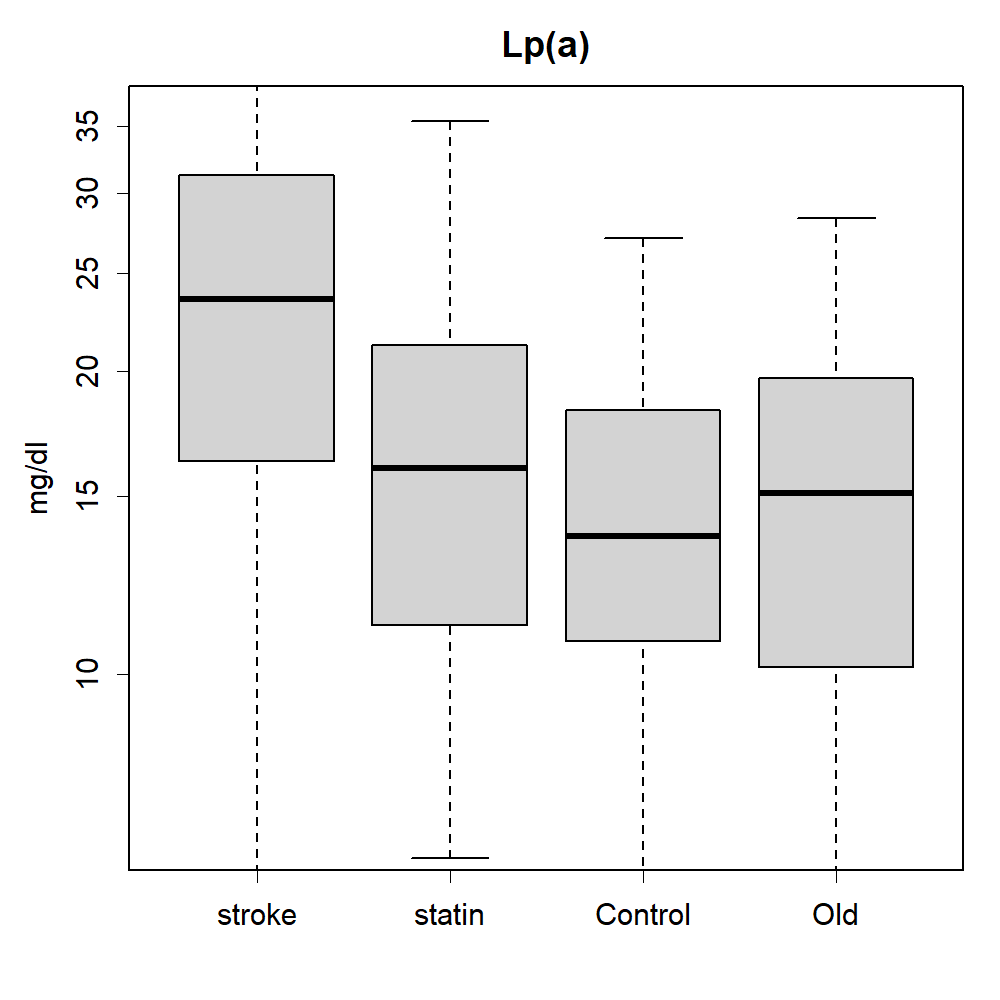

Supplement: S1 File — (ZIP) [file pone.0283855.s001.zip › supplement/CH/Lp(a).png]

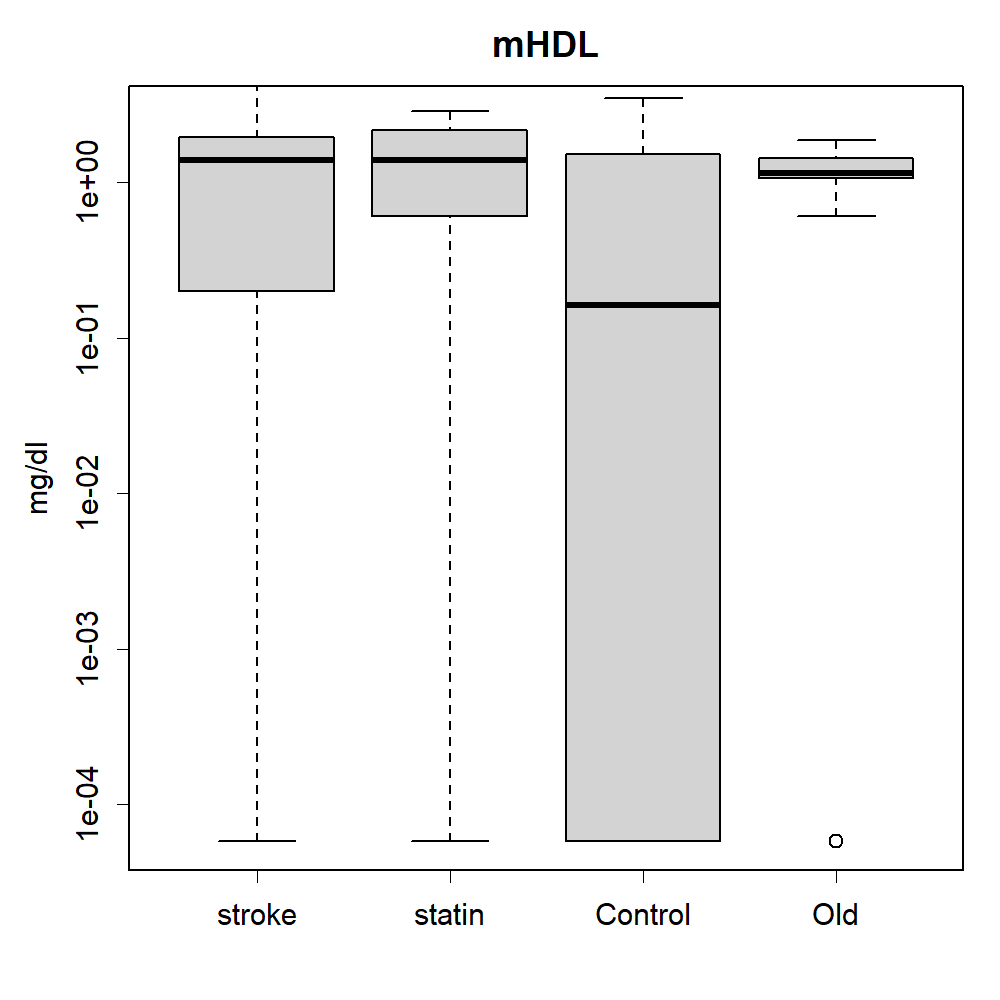

Supplement: S1 File — (ZIP) [file pone.0283855.s001.zip › supplement/CH/mHDL.png]

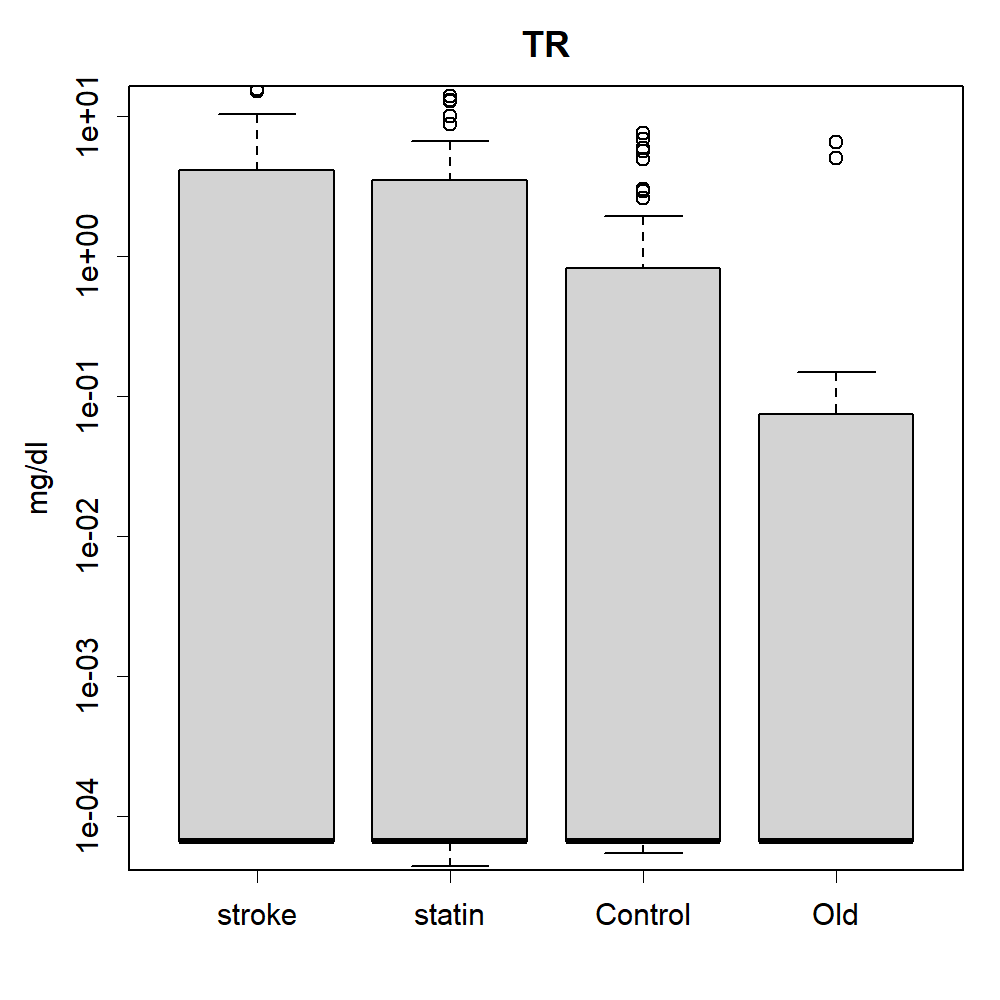

Supplement: S1 File — (ZIP) [file pone.0283855.s001.zip › supplement/CH/TR.png]

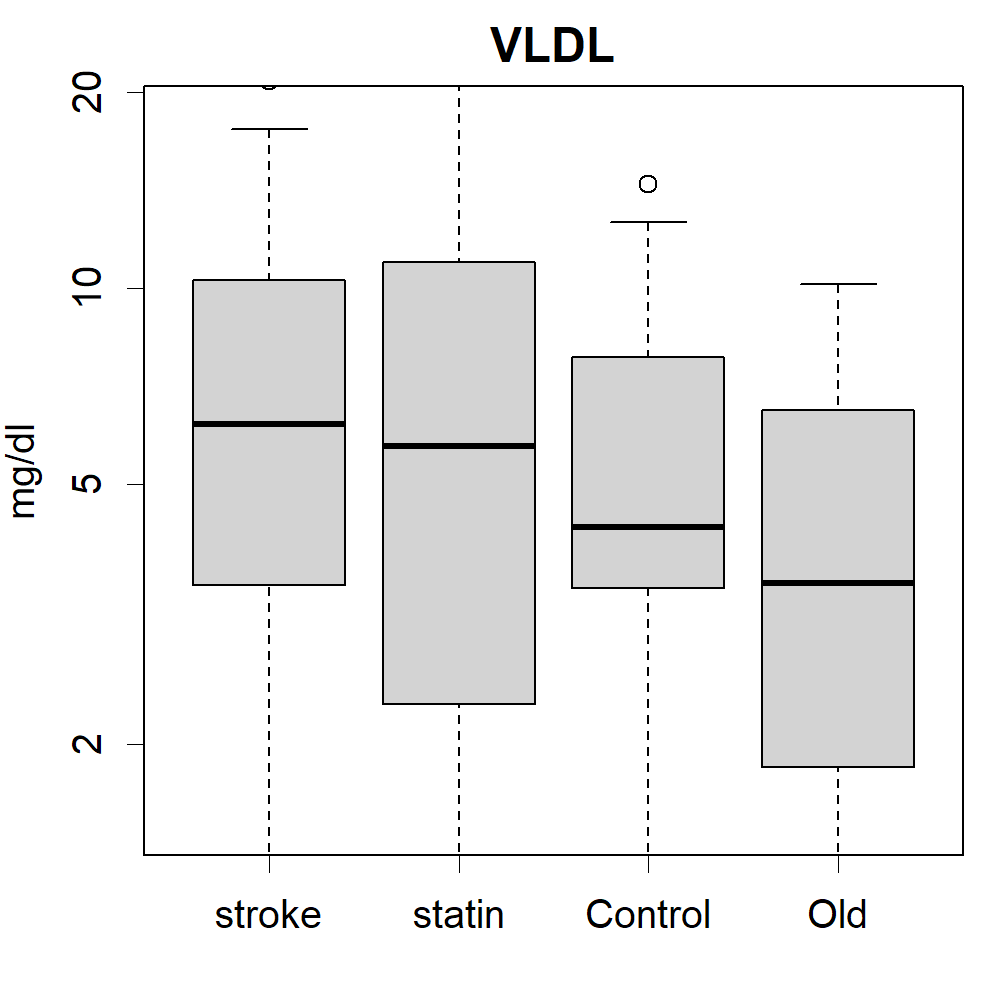

Supplement: S1 File — (ZIP) [file pone.0283855.s001.zip › supplement/CH/VLDL.png]

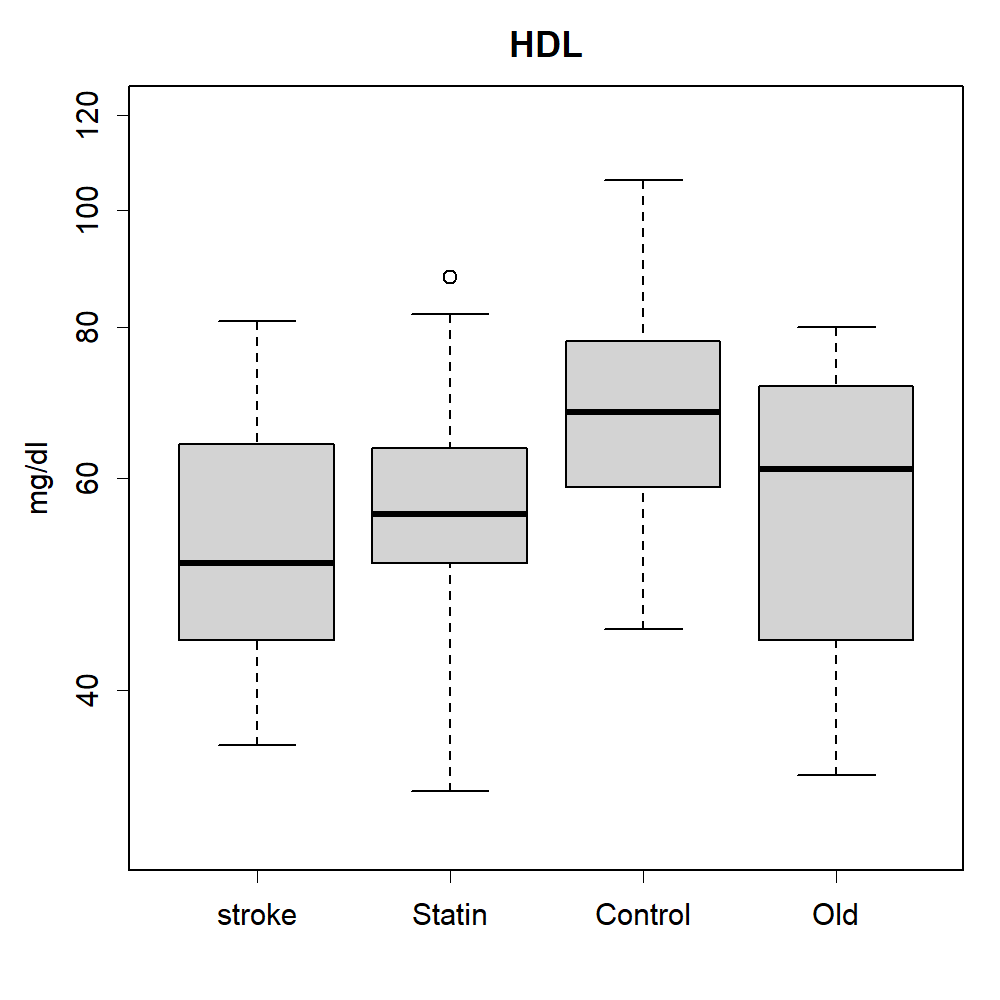

Supplement: S1 File — (ZIP) [file pone.0283855.s001.zip › supplement/curr/Box2HDL.png]

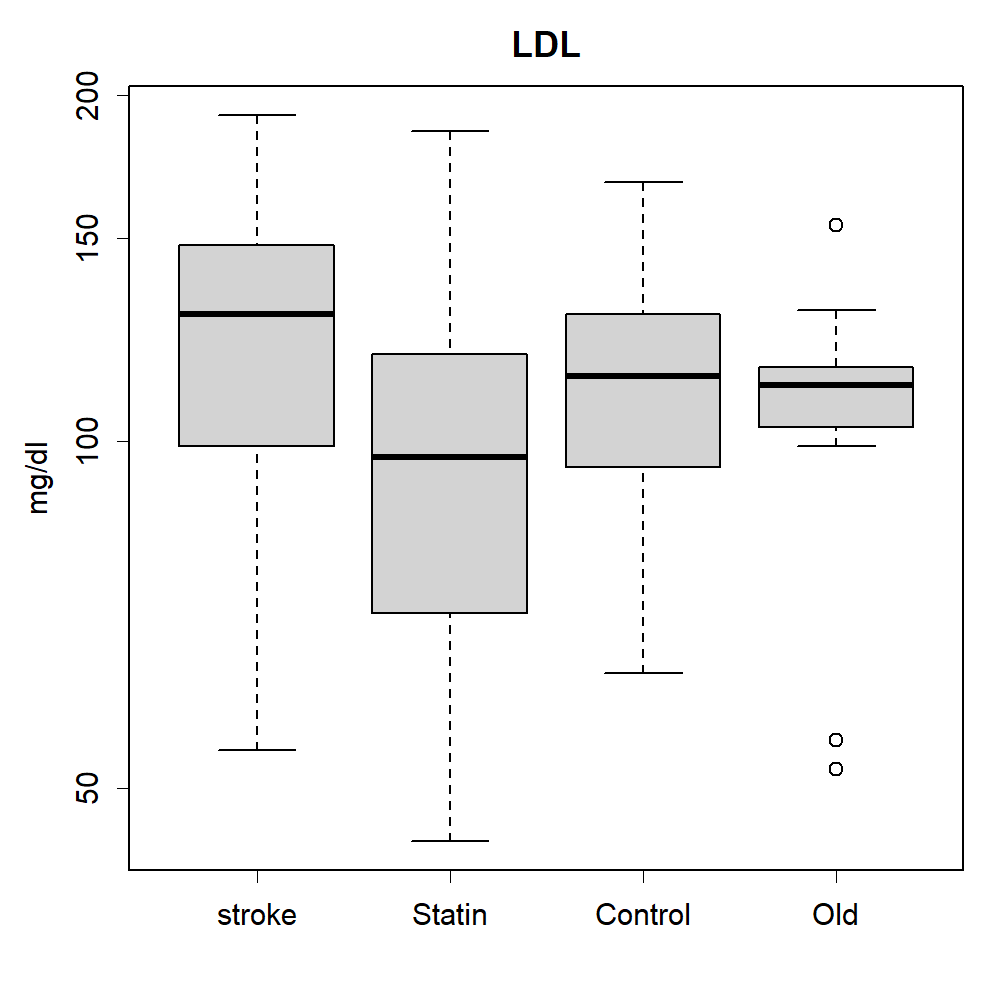

Supplement: S1 File — (ZIP) [file pone.0283855.s001.zip › supplement/curr/Box2LDL.png]

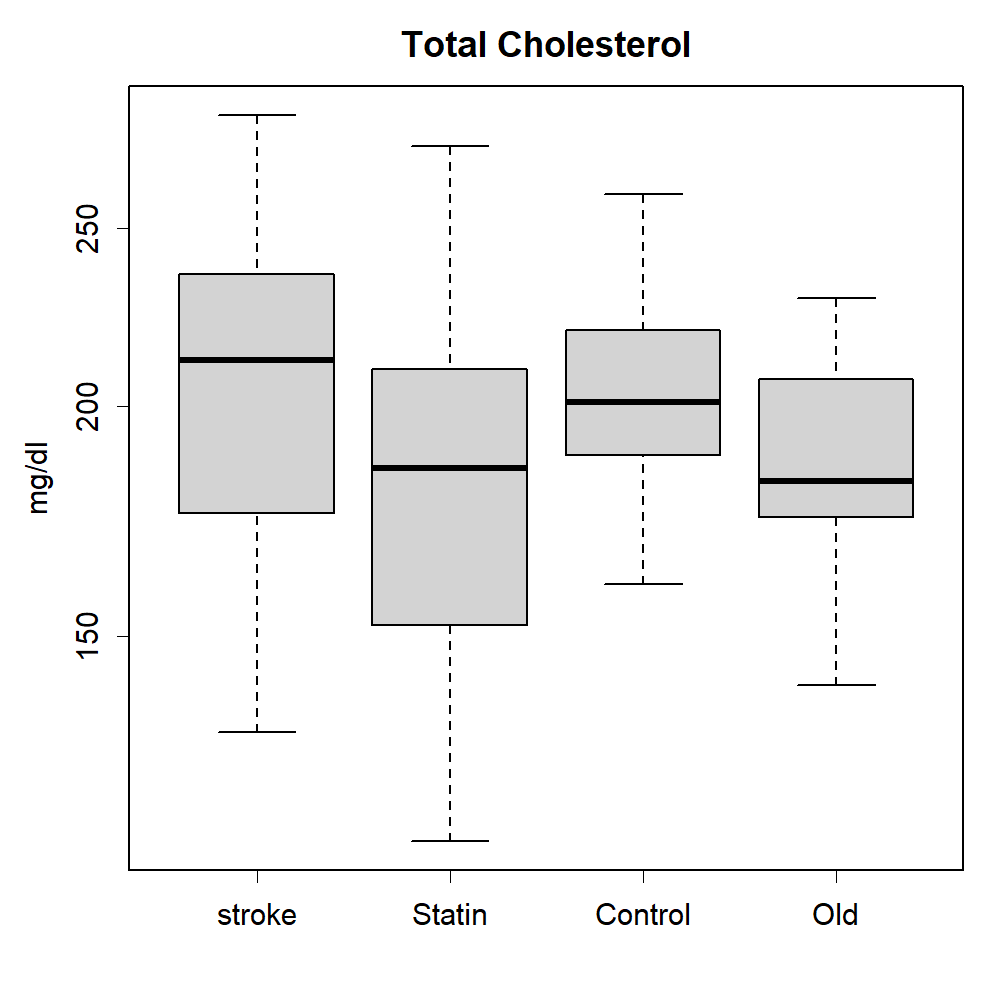

Supplement: S1 File — (ZIP) [file pone.0283855.s001.zip › supplement/curr/Box2Tch.png]

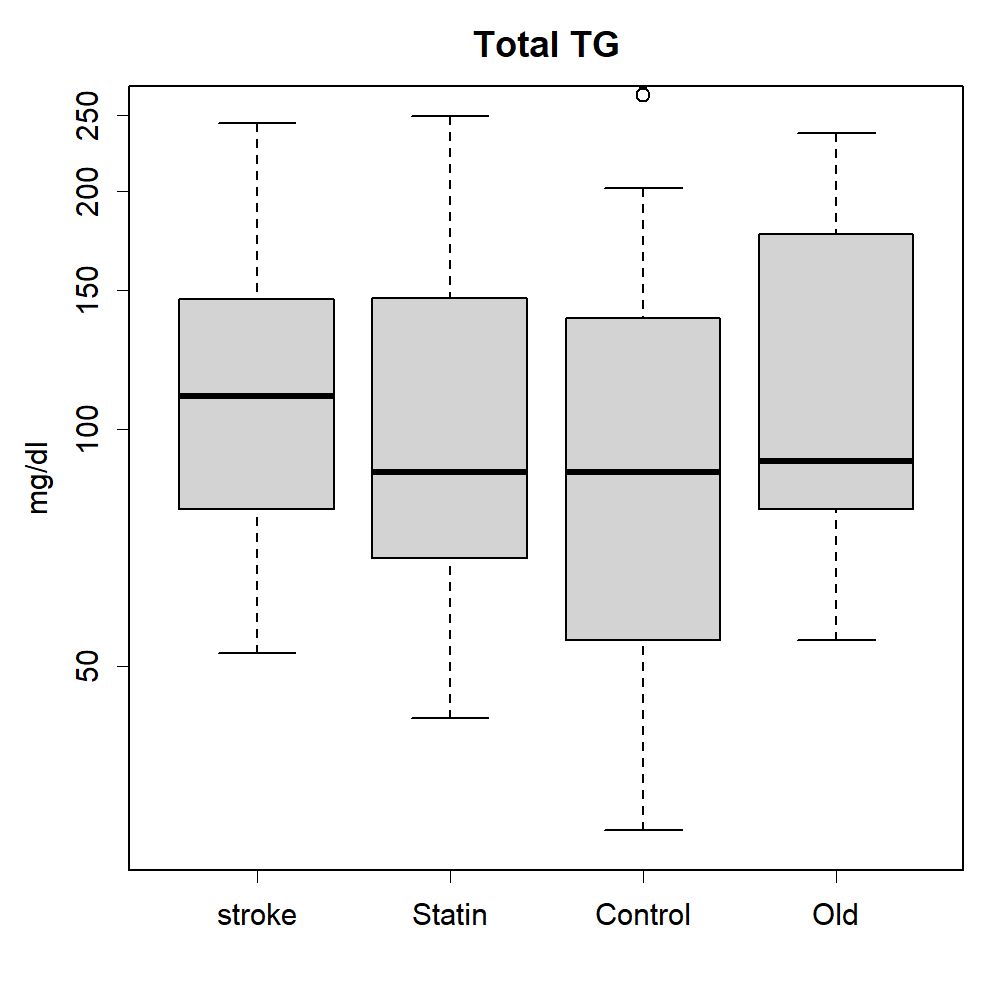

Supplement: S1 File — (ZIP) [file pone.0283855.s001.zip › supplement/curr/Box2TG.png]

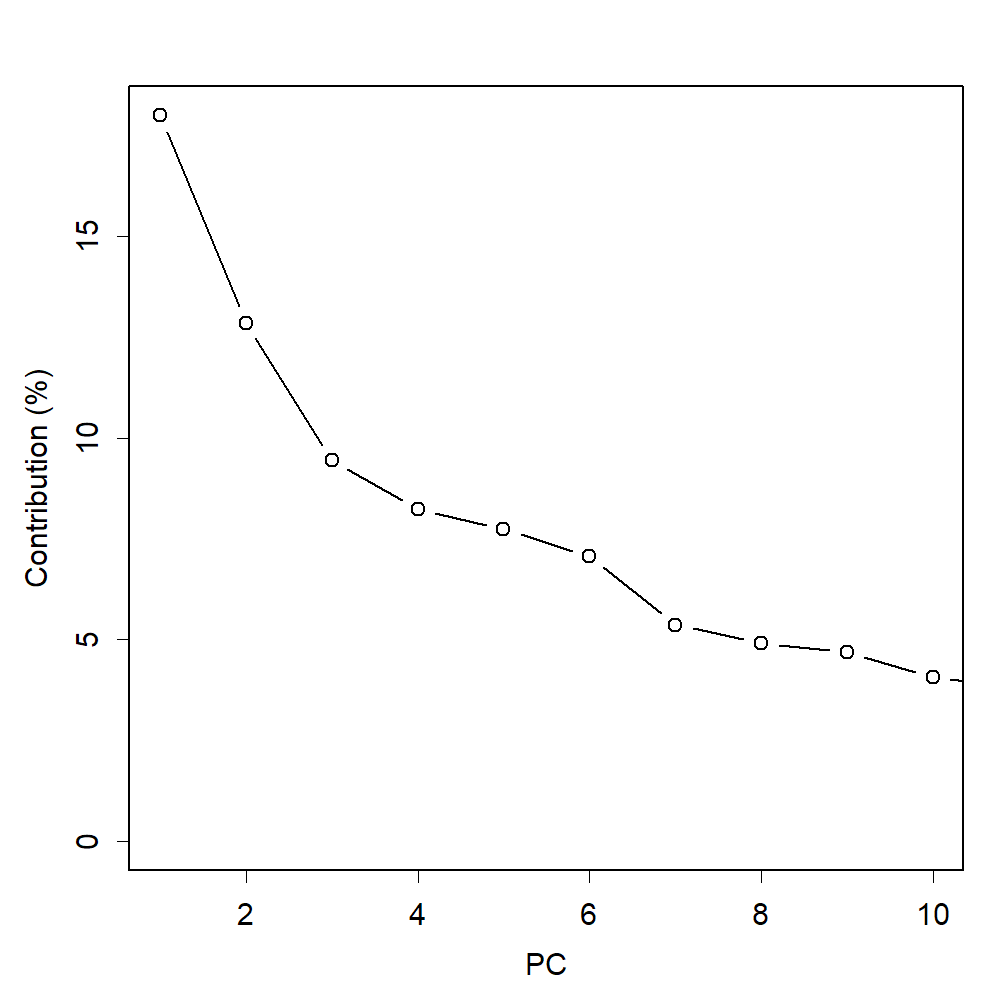

Supplement: S1 File — (ZIP) [file pone.0283855.s001.zip › supplement/ind/contribution.png]

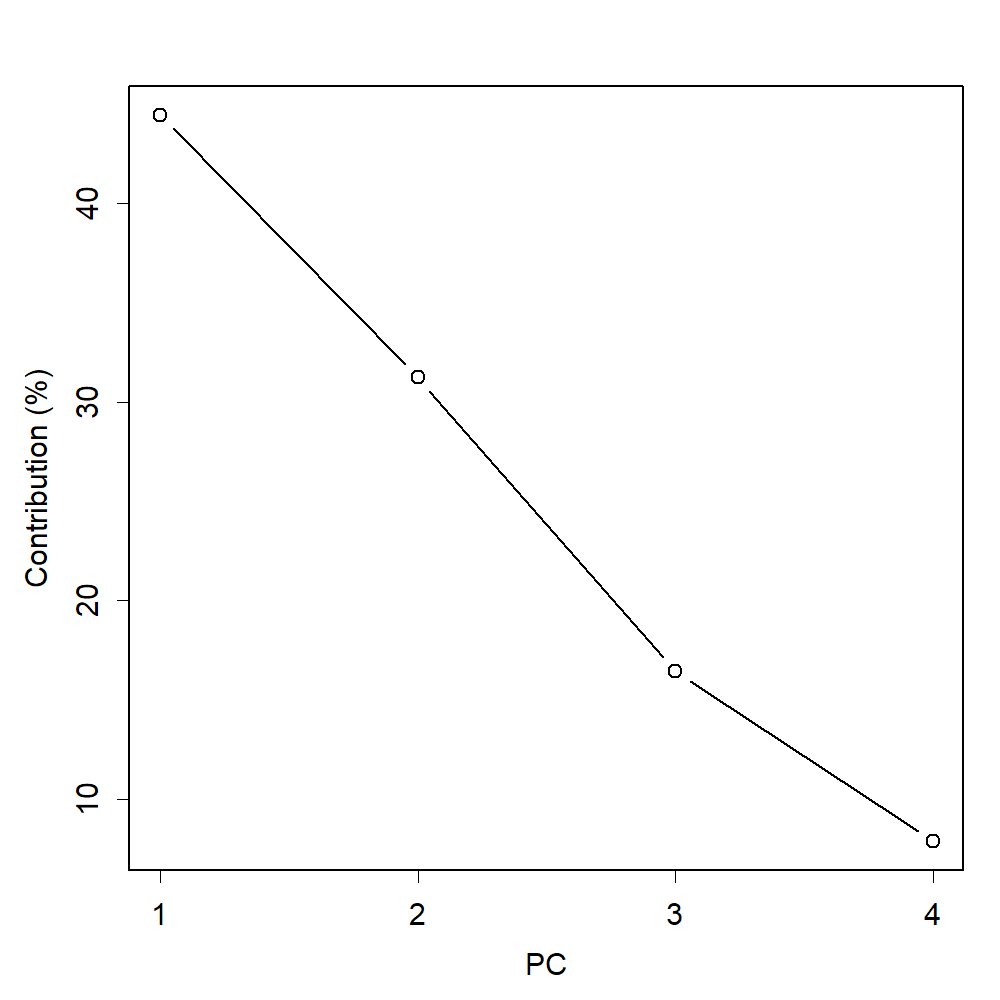

Supplement: S1 File — (ZIP) [file pone.0283855.s001.zip › supplement/ind/contribution2.png]

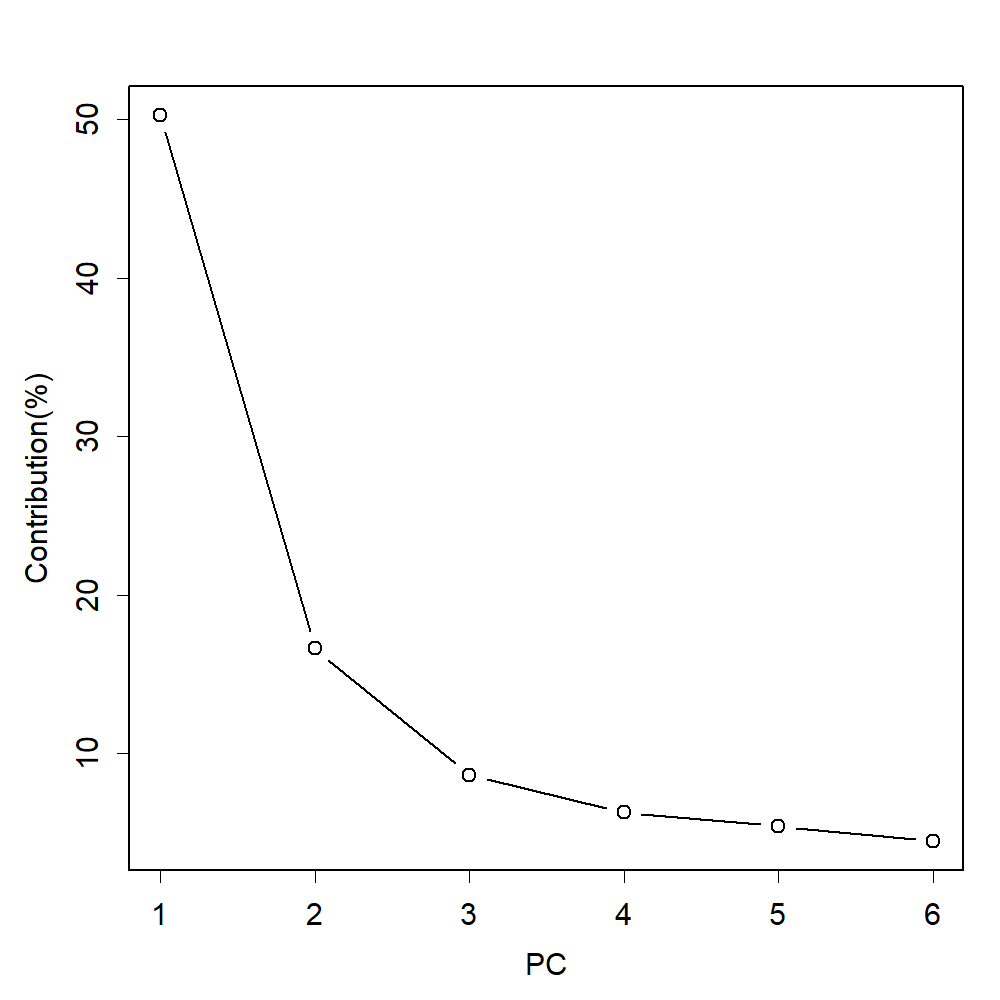

Supplement: S1 File — (ZIP) [file pone.0283855.s001.zip › supplement/ind/contribution3.png]

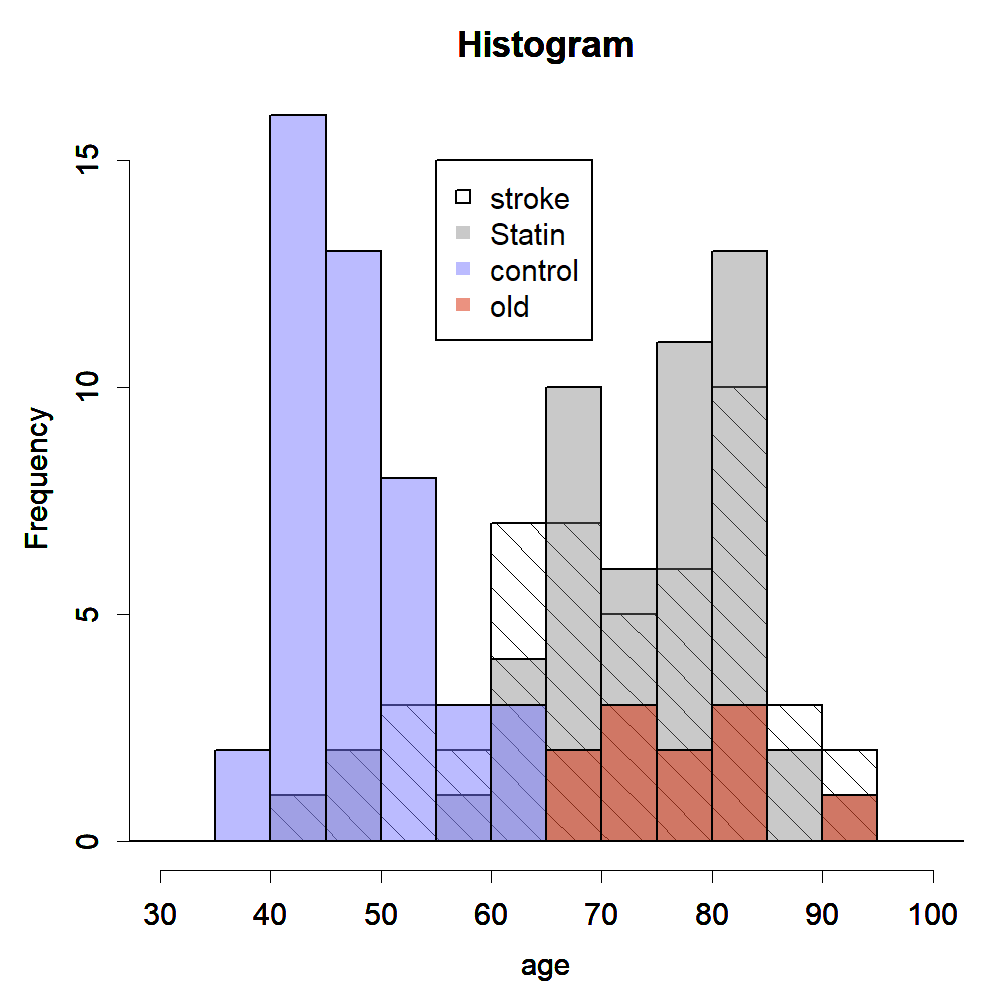

Supplement: S1 File — (ZIP) [file pone.0283855.s001.zip › supplement/ind/histAge2.png]

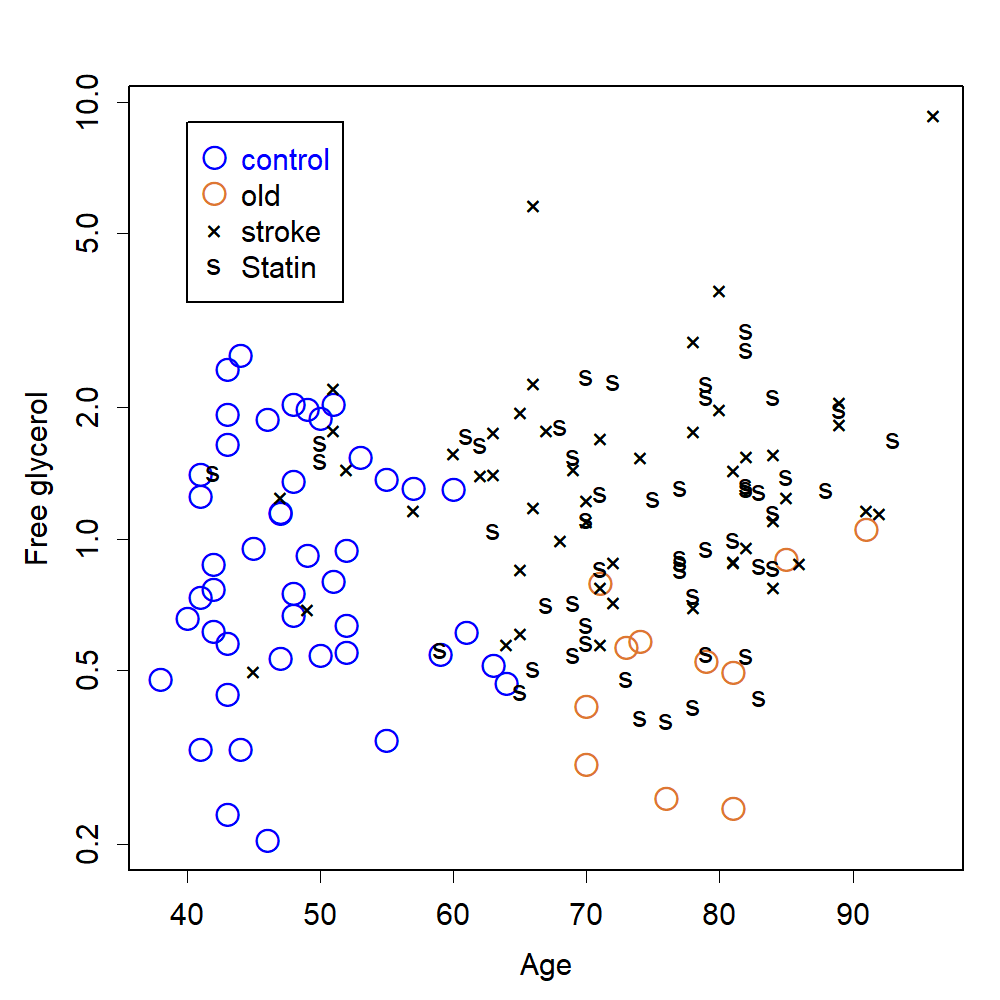

Supplement: S1 File — (ZIP) [file pone.0283855.s001.zip › supplement/PC/age.png]

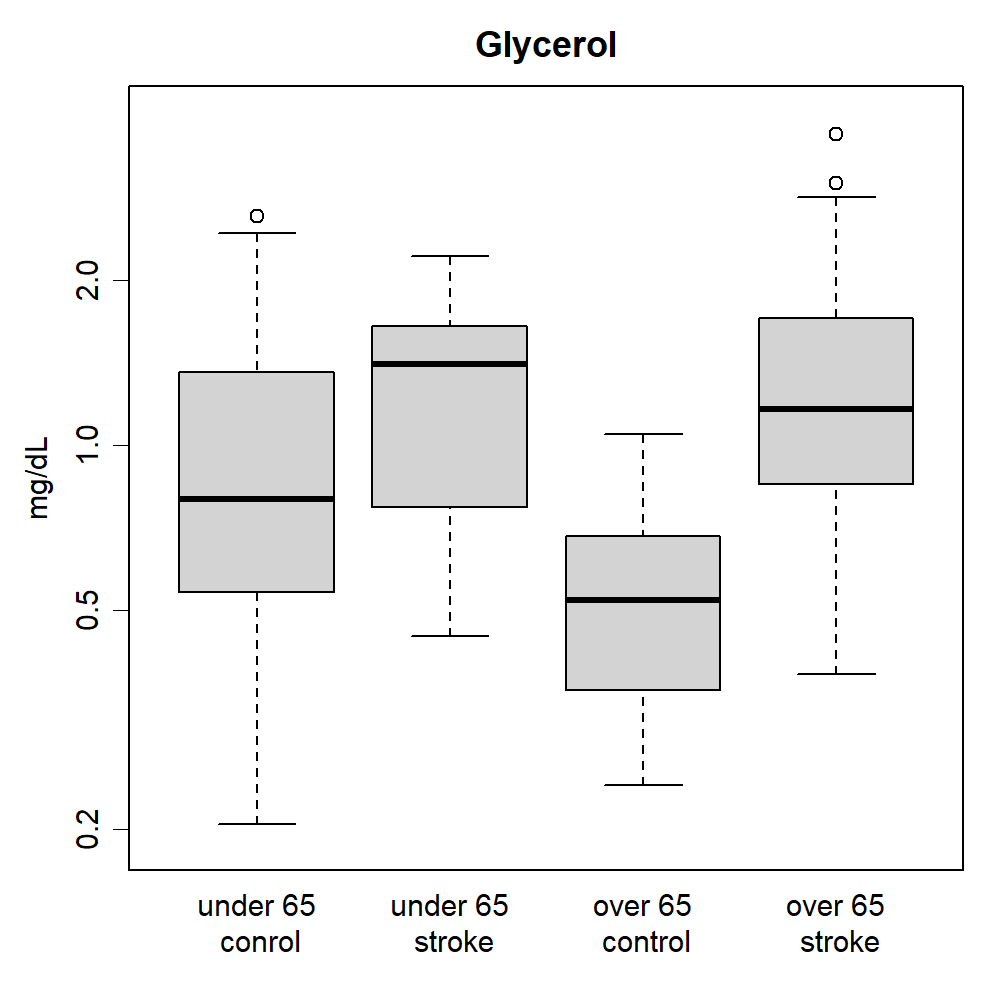

Supplement: S1 File — (ZIP) [file pone.0283855.s001.zip › supplement/PC/agebox.png]

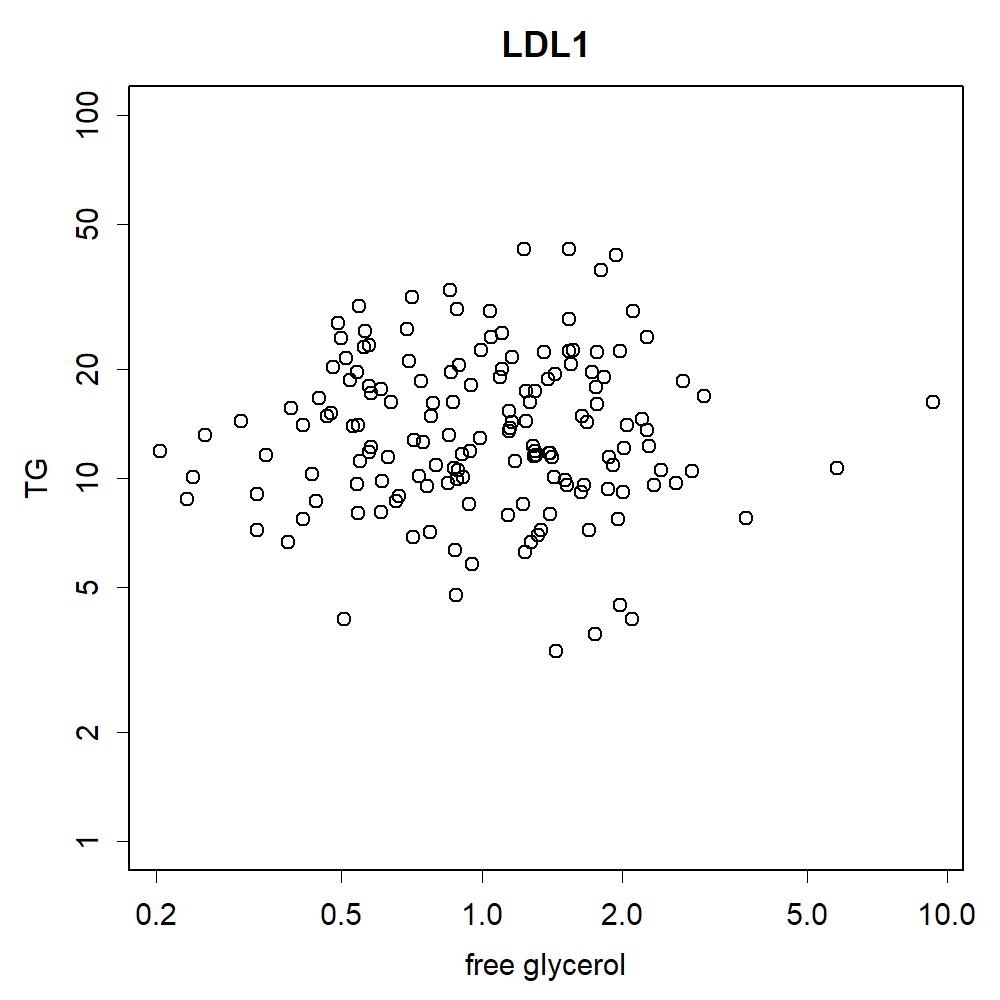

Supplement: S1 File — (ZIP) [file pone.0283855.s001.zip › supplement/PC/glycerolLDL1.png]

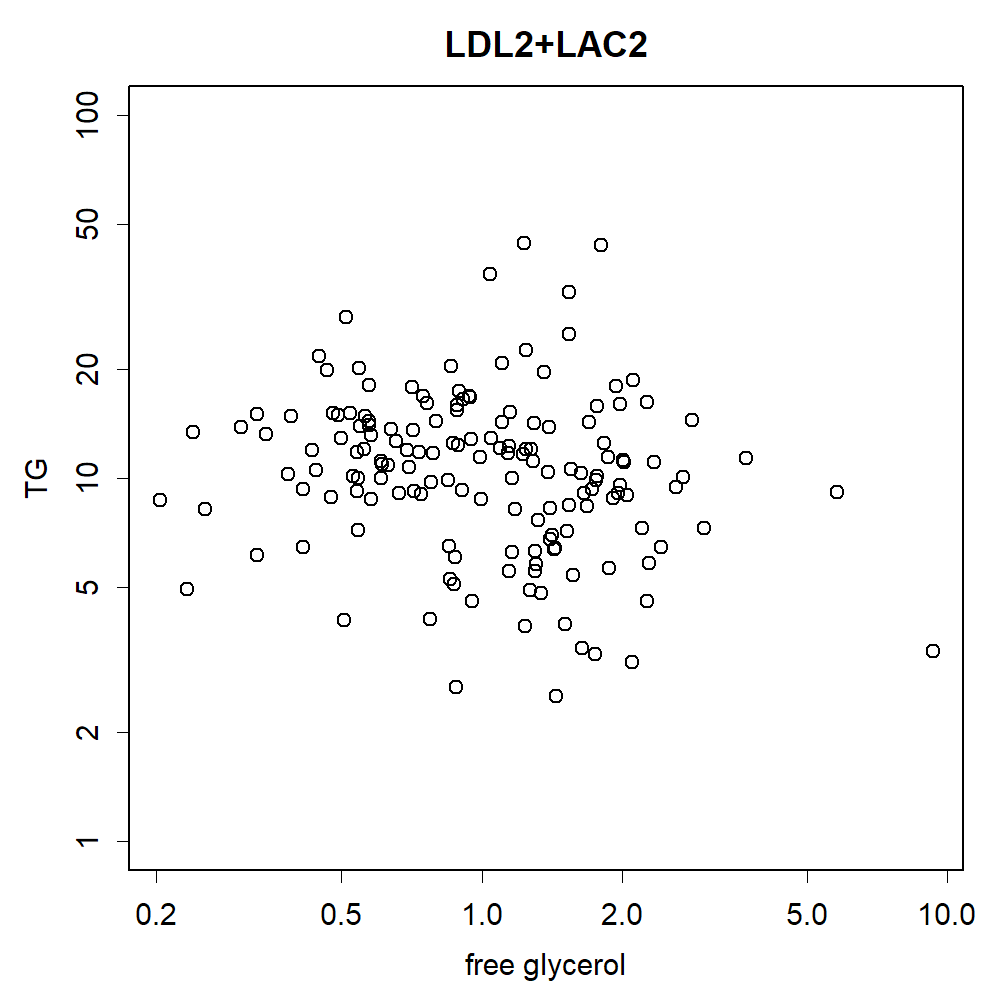

Supplement: S1 File — (ZIP) [file pone.0283855.s001.zip › supplement/PC/glycerolLDL2.png]

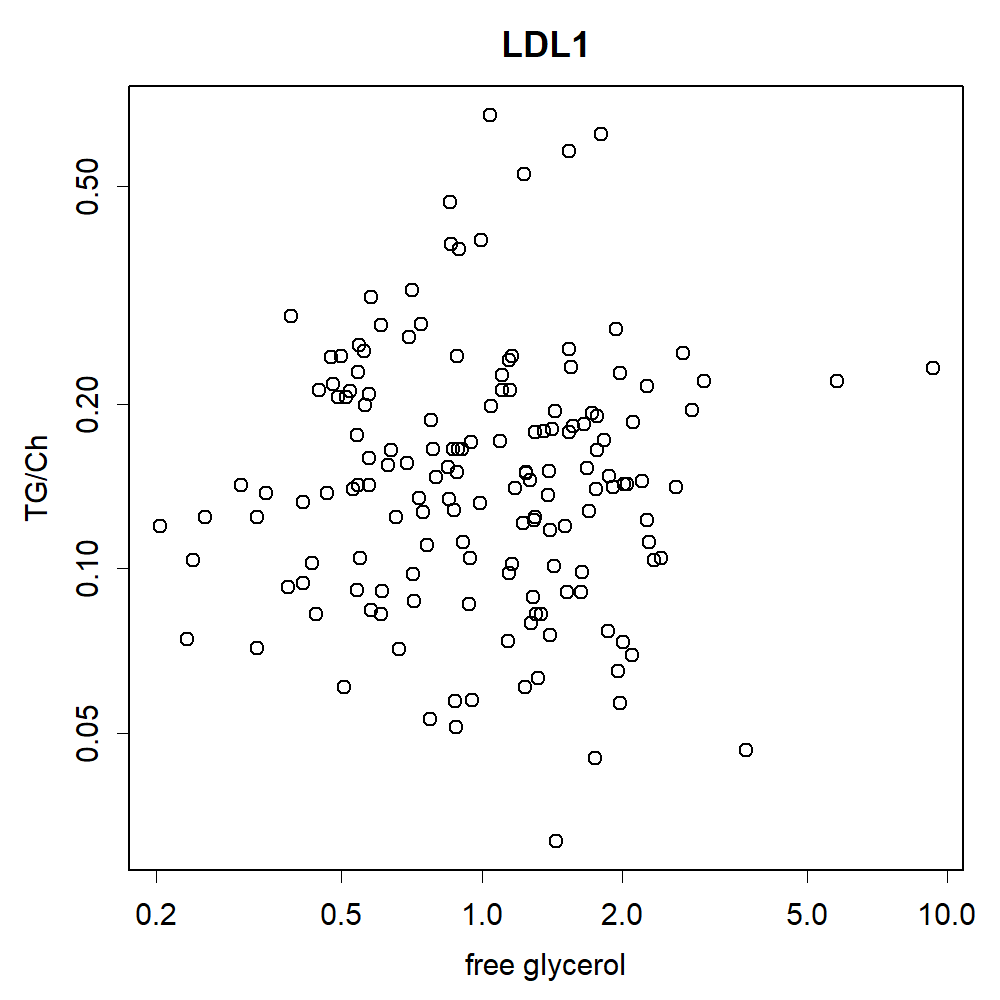

Supplement: S1 File — (ZIP) [file pone.0283855.s001.zip › supplement/PC/glycerolratio.png]

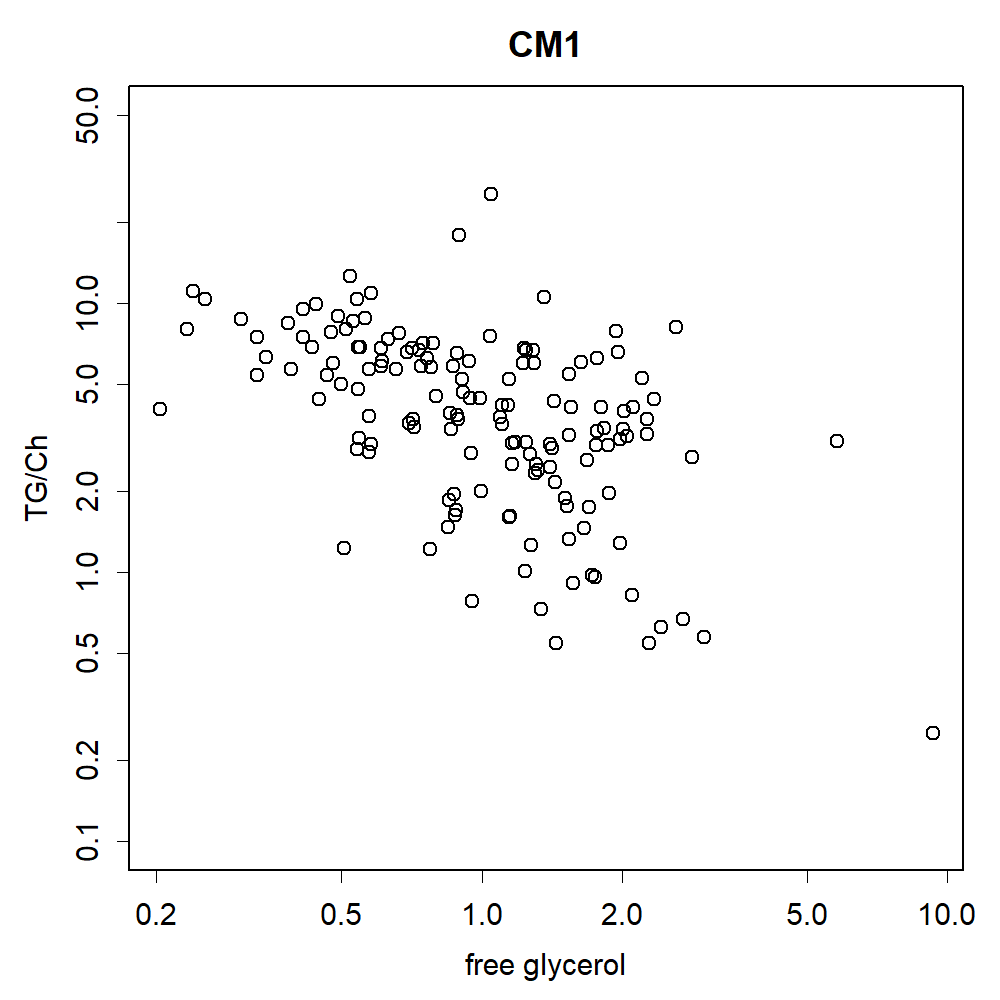

Supplement: S1 File — (ZIP) [file pone.0283855.s001.zip › supplement/PC/glycerolratioCM1.png]

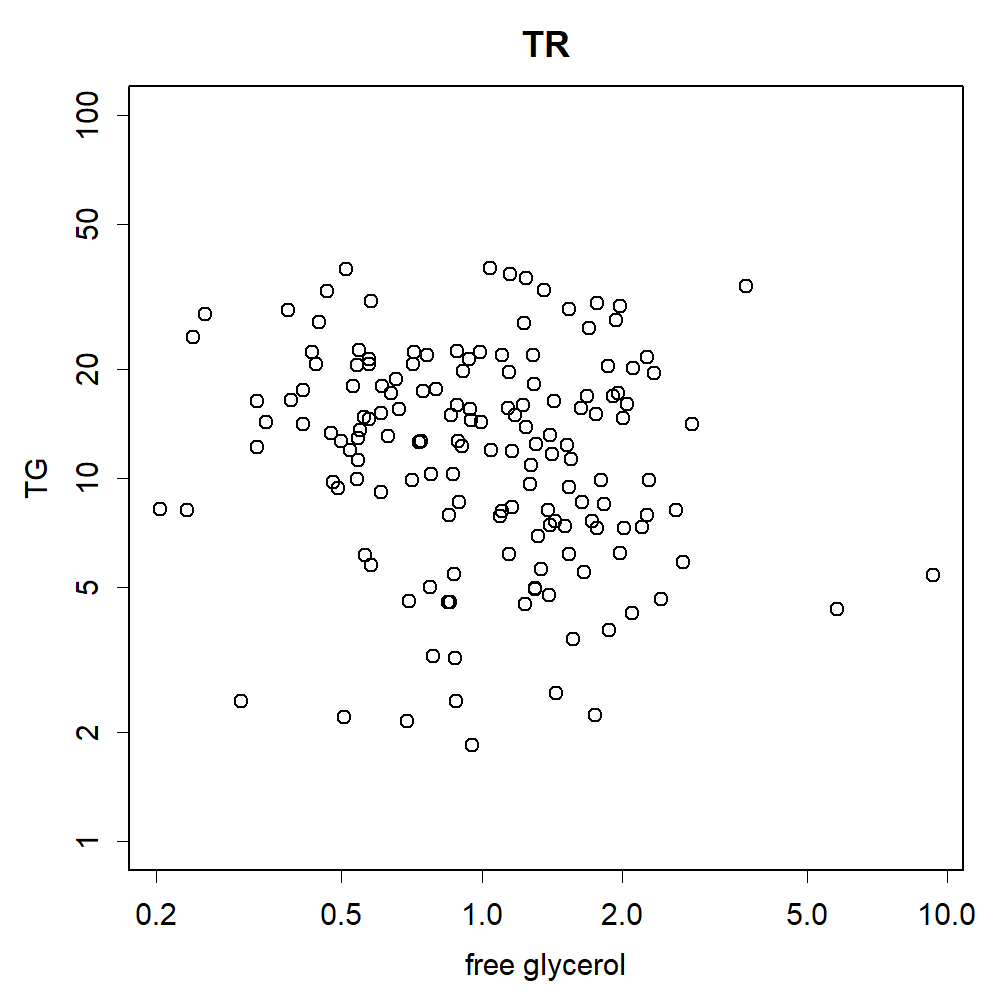

Supplement: S1 File — (ZIP) [file pone.0283855.s001.zip › supplement/PC/glycerolTR.png]

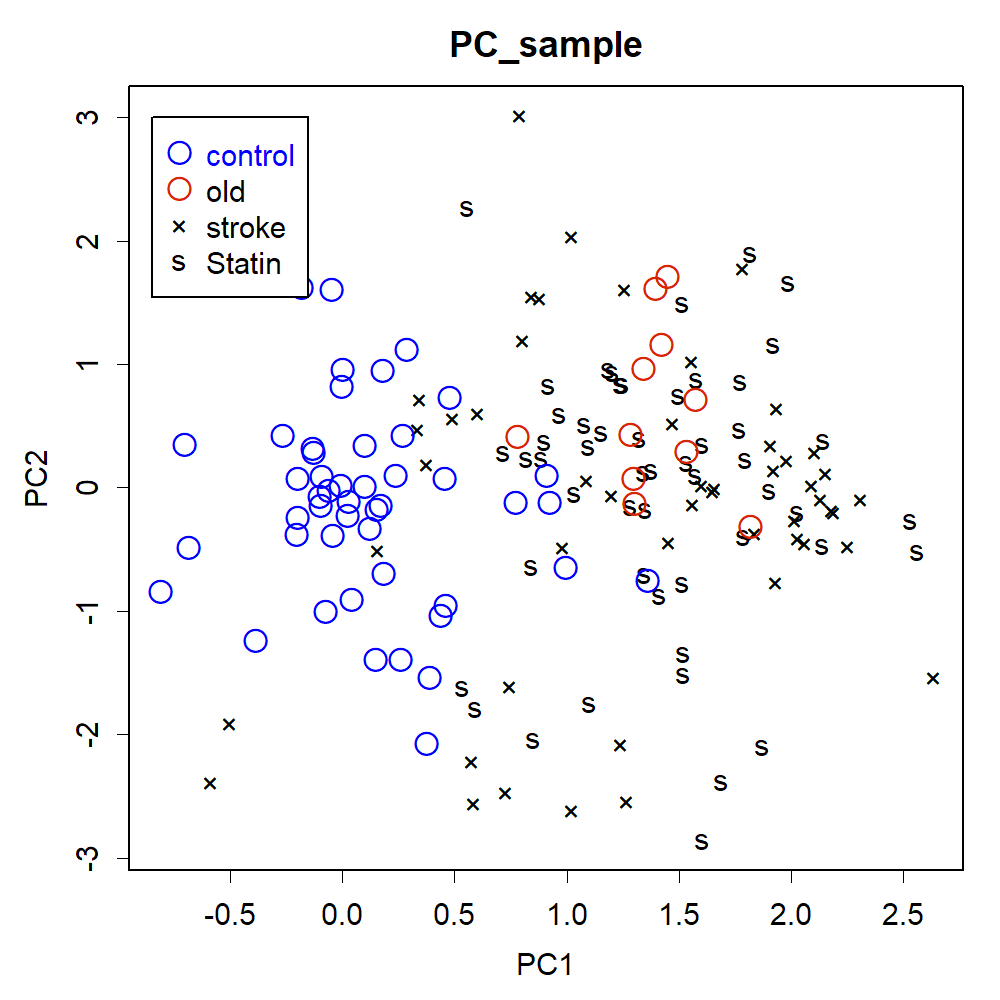

Supplement: S1 File — (ZIP) [file pone.0283855.s001.zip › supplement/PC/PC12.png]

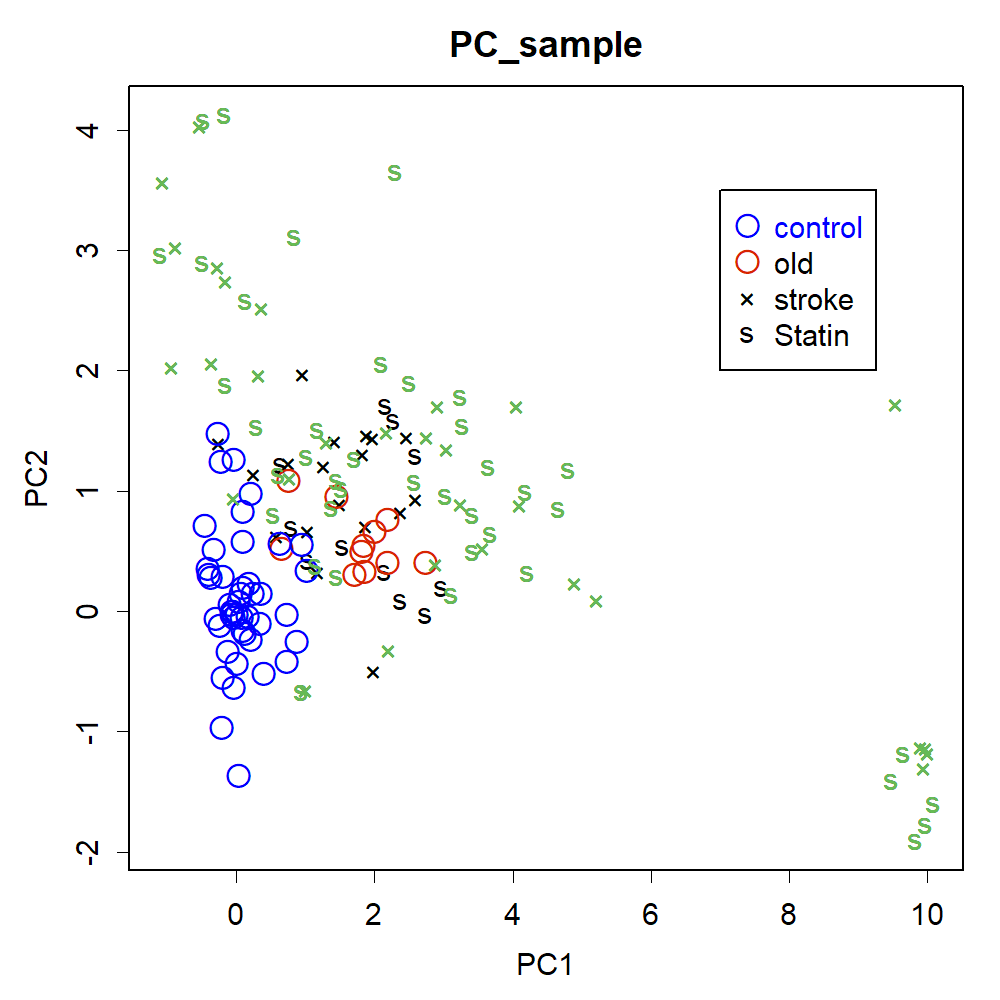

Supplement: S1 File — (ZIP) [file pone.0283855.s001.zip › supplement/PC/PC12A.png]

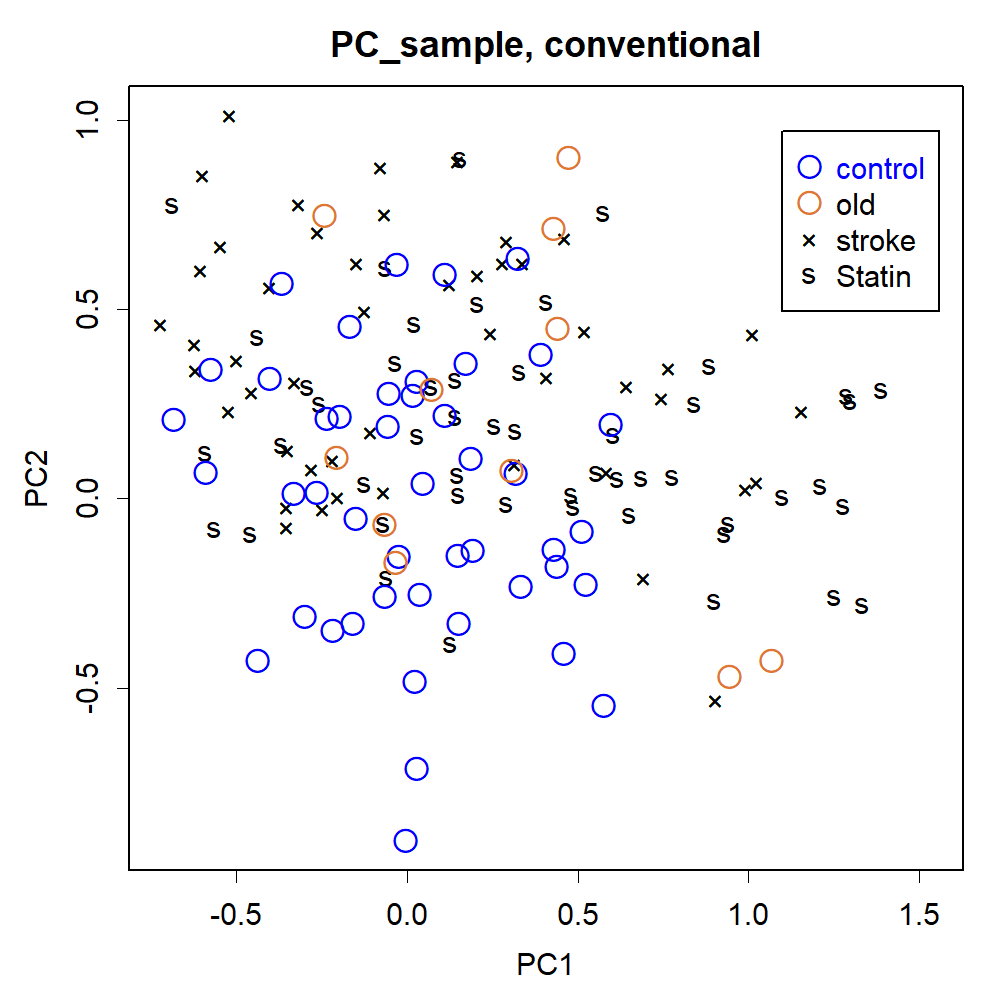

Supplement: S1 File — (ZIP) [file pone.0283855.s001.zip › supplement/PC/PC12Furui.png]

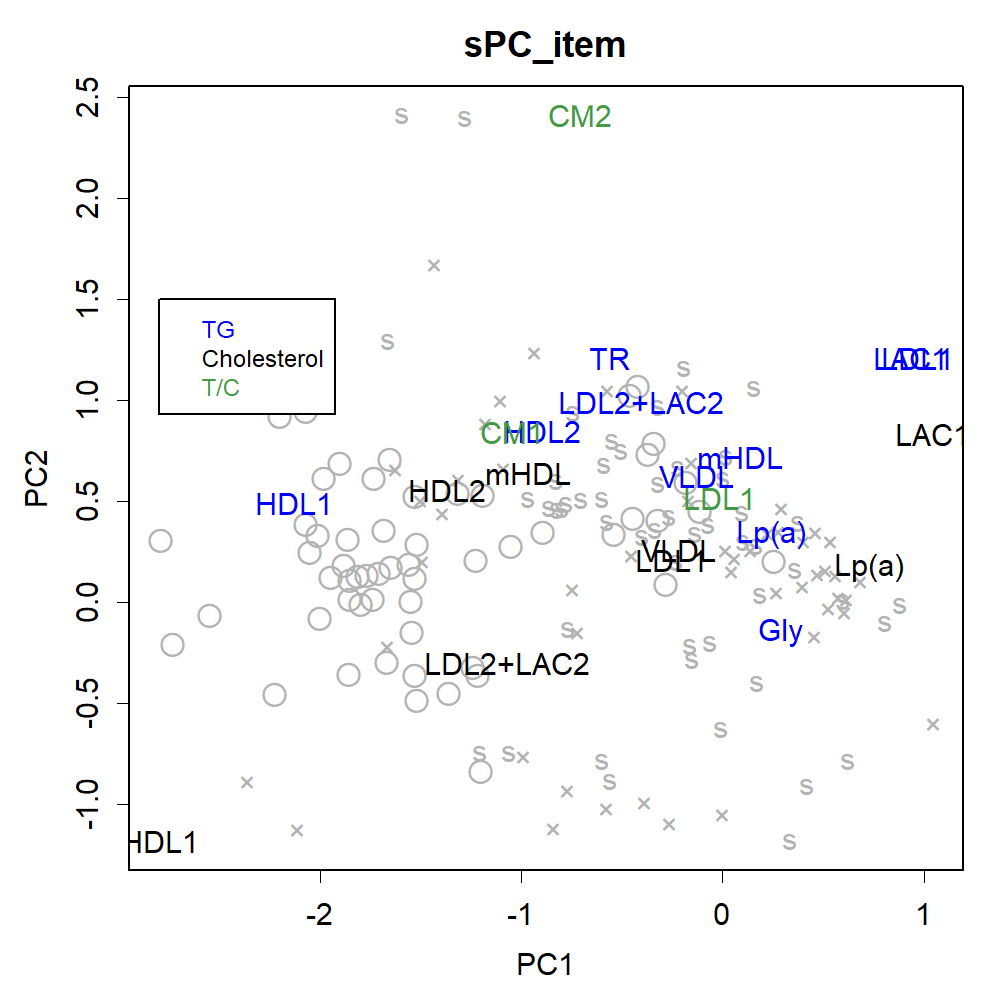

Supplement: S1 File — (ZIP) [file pone.0283855.s001.zip › supplement/PC/PC12item.png]

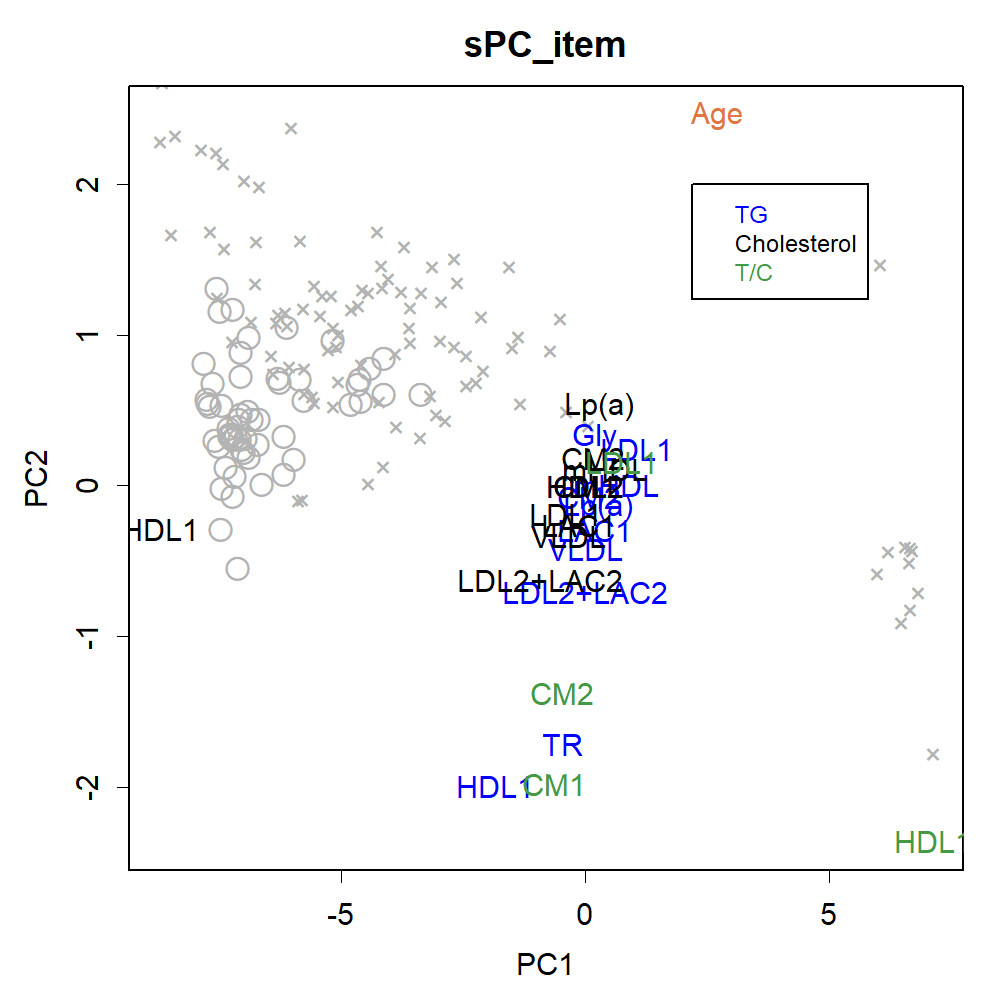

Supplement: S1 File — (ZIP) [file pone.0283855.s001.zip › supplement/PC/PC12itemA.png]

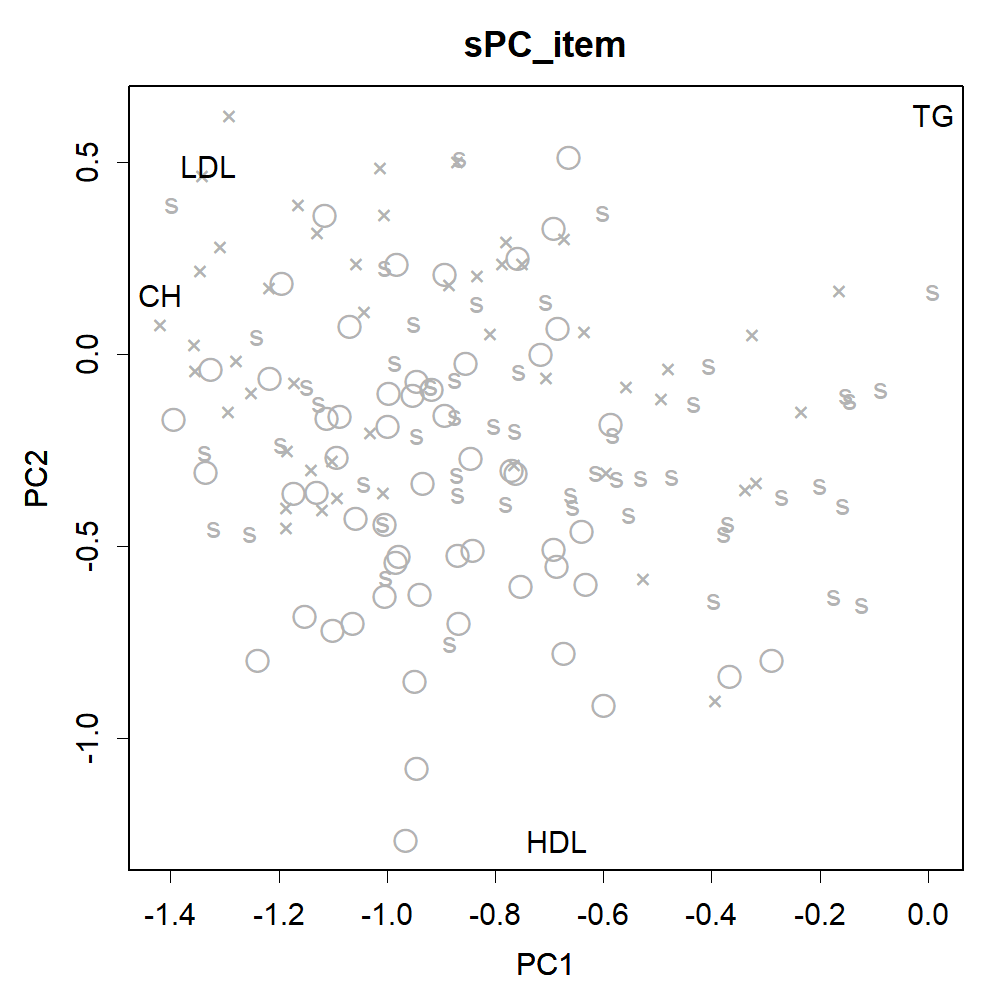

Supplement: S1 File — (ZIP) [file pone.0283855.s001.zip › supplement/PC/PC12itemFurui.png]

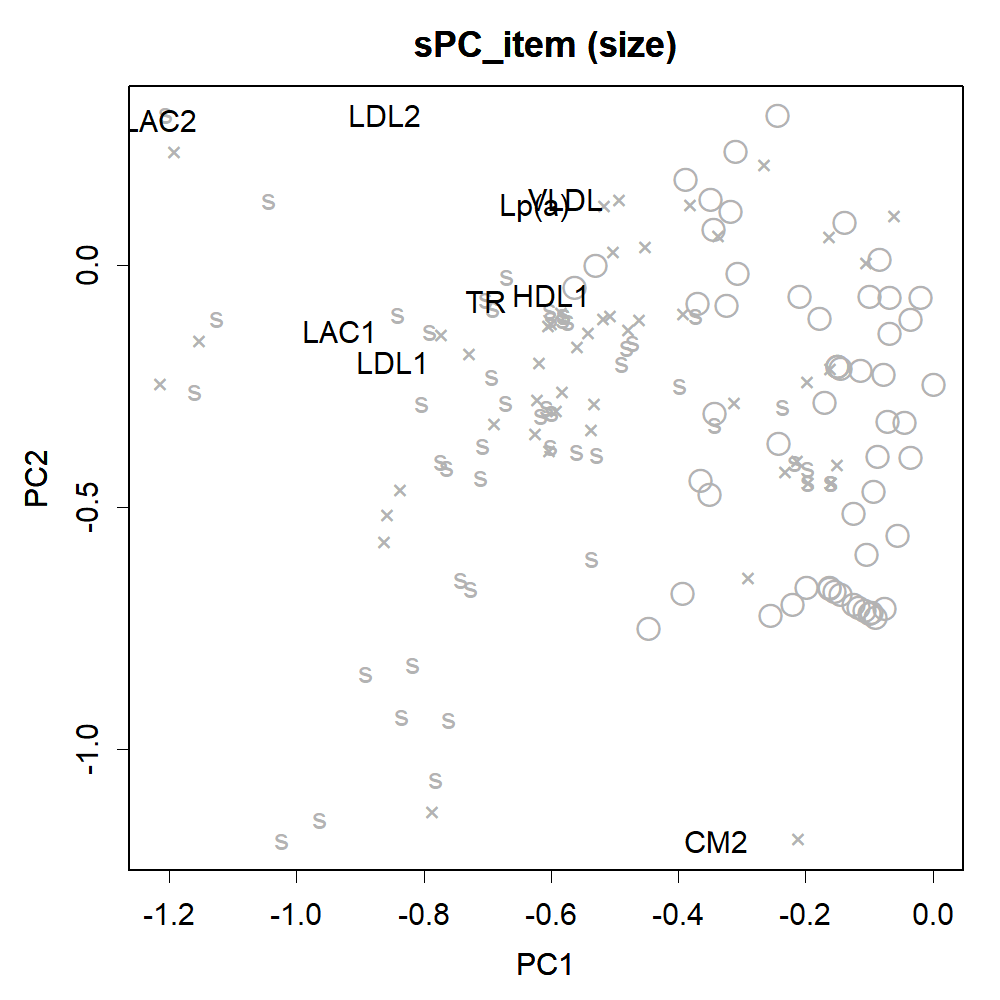

Supplement: S1 File — (ZIP) [file pone.0283855.s001.zip › supplement/PC/PC12itemOOKISA.png]

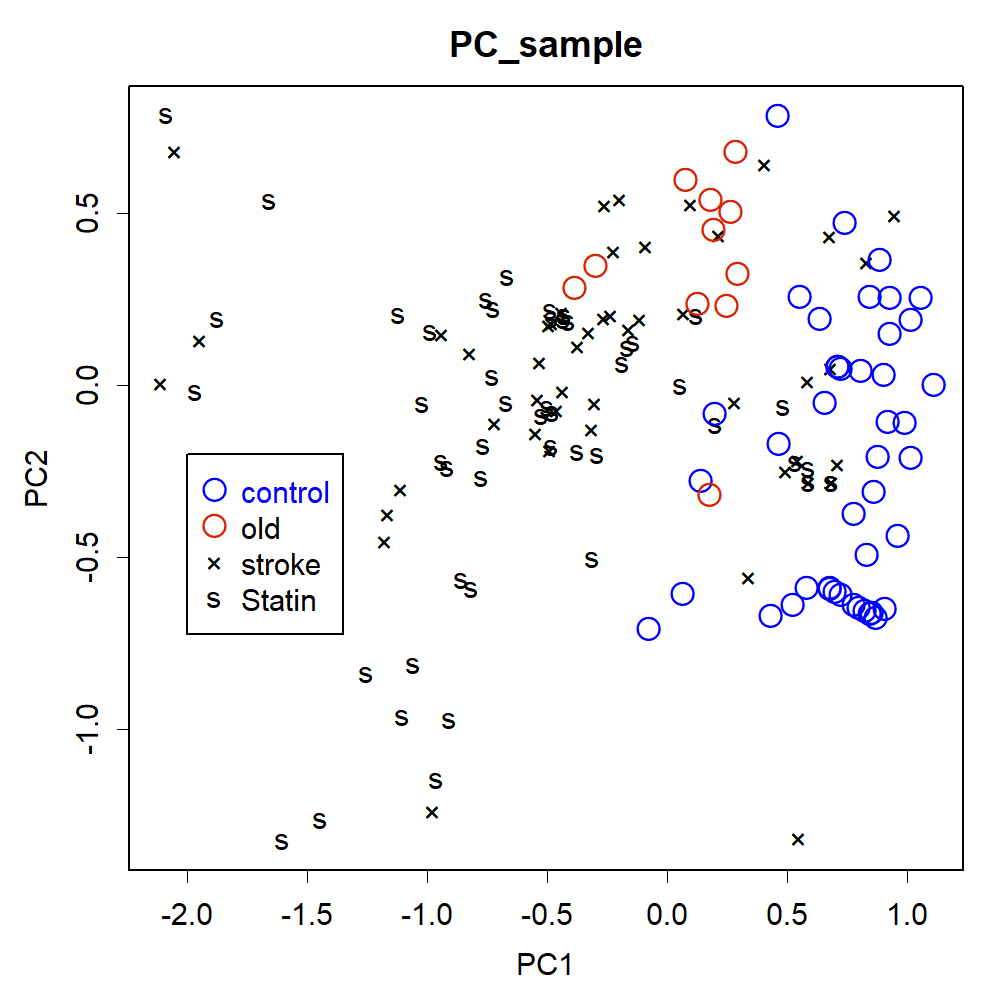

Supplement: S1 File — (ZIP) [file pone.0283855.s001.zip › supplement/PC/PC12ookisa.png]

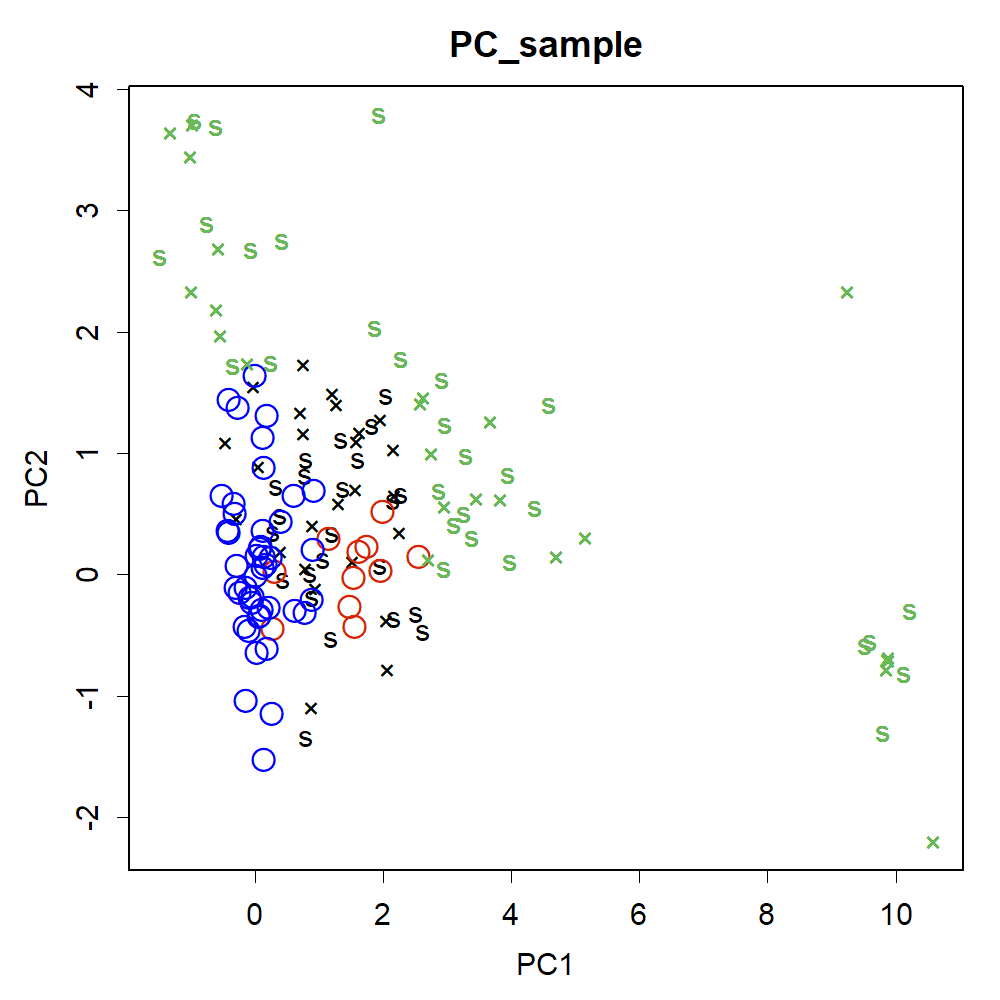

Supplement: S1 File — (ZIP) [file pone.0283855.s001.zip › supplement/PC/PC12orig.png]

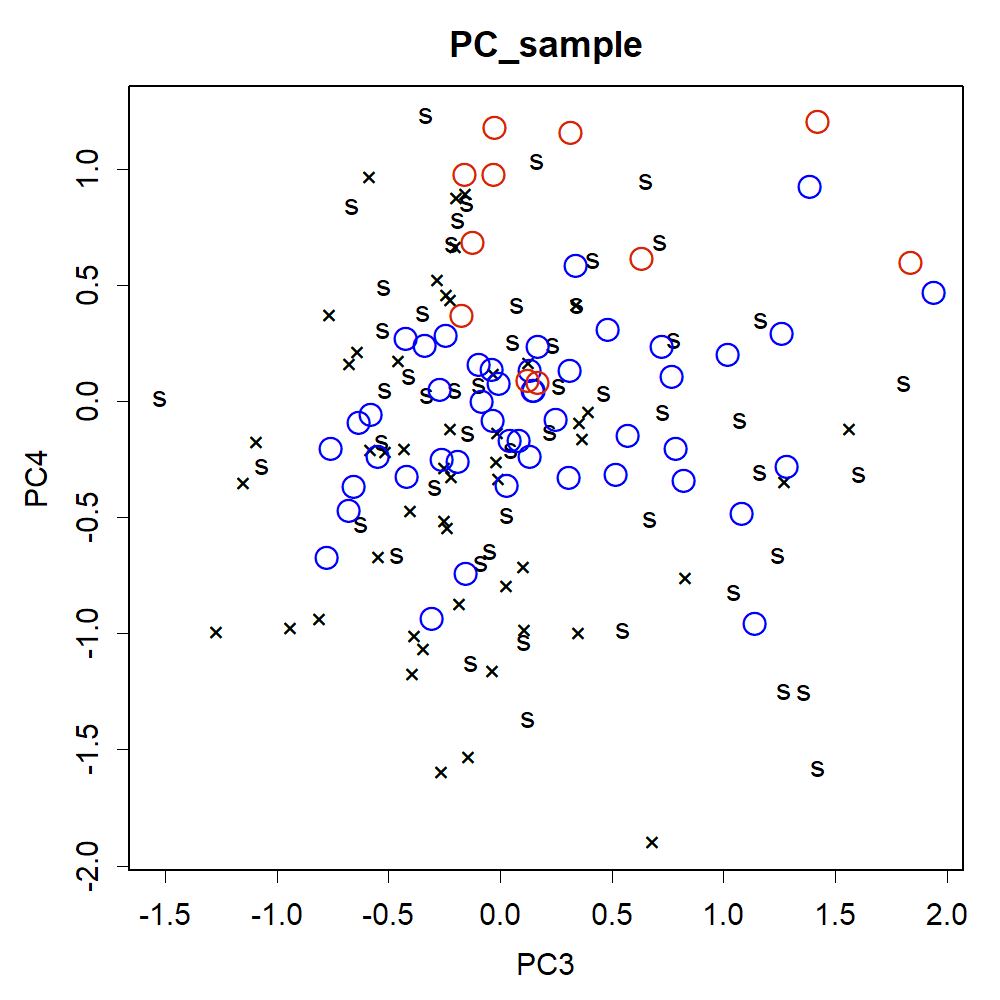

Supplement: S1 File — (ZIP) [file pone.0283855.s001.zip › supplement/PC/PC34.png]

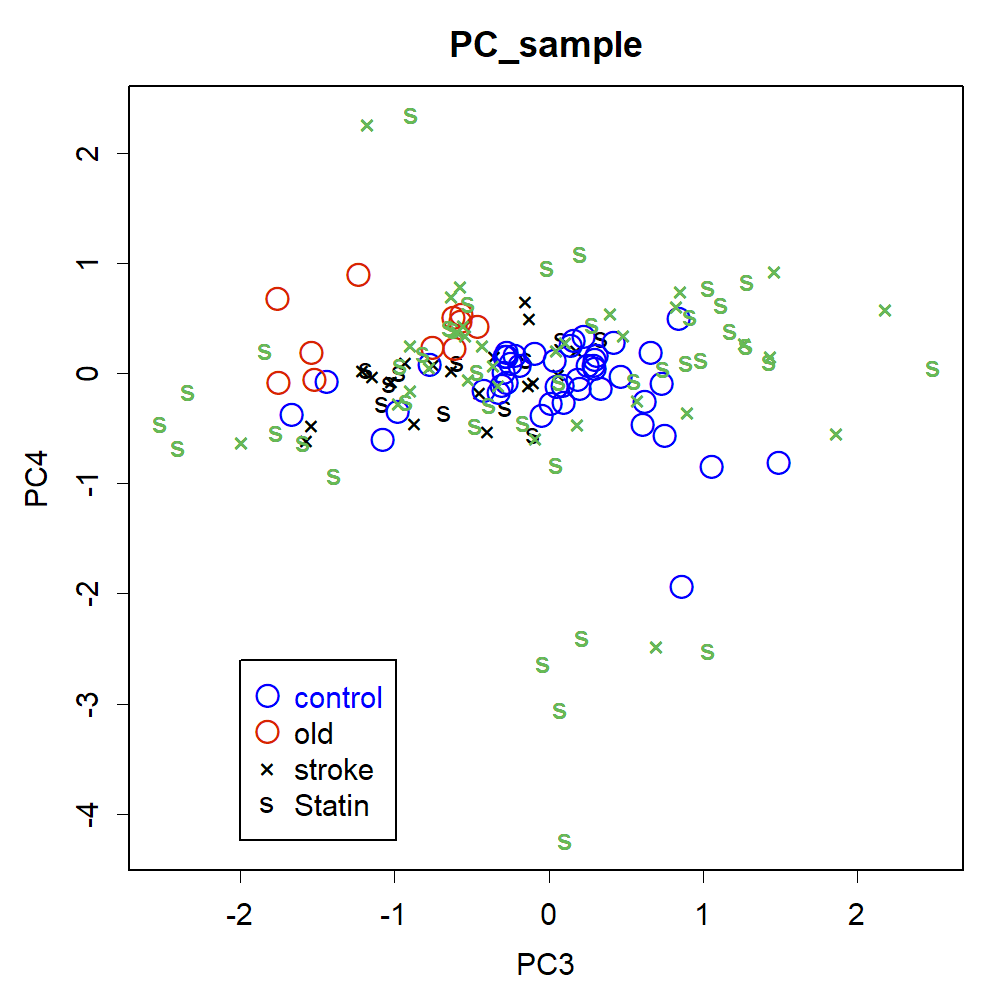

Supplement: S1 File — (ZIP) [file pone.0283855.s001.zip › supplement/PC/PC34A.png]

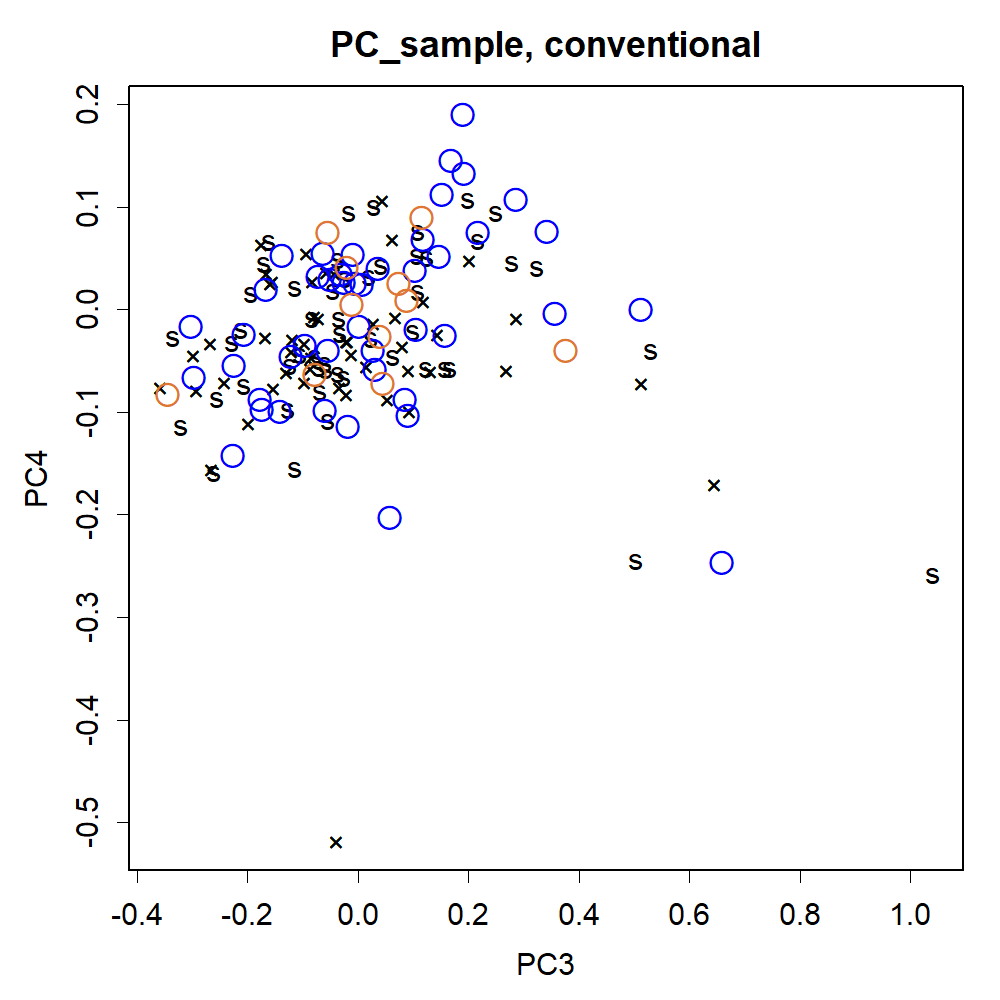

Supplement: S1 File — (ZIP) [file pone.0283855.s001.zip › supplement/PC/PC34Furui.png]

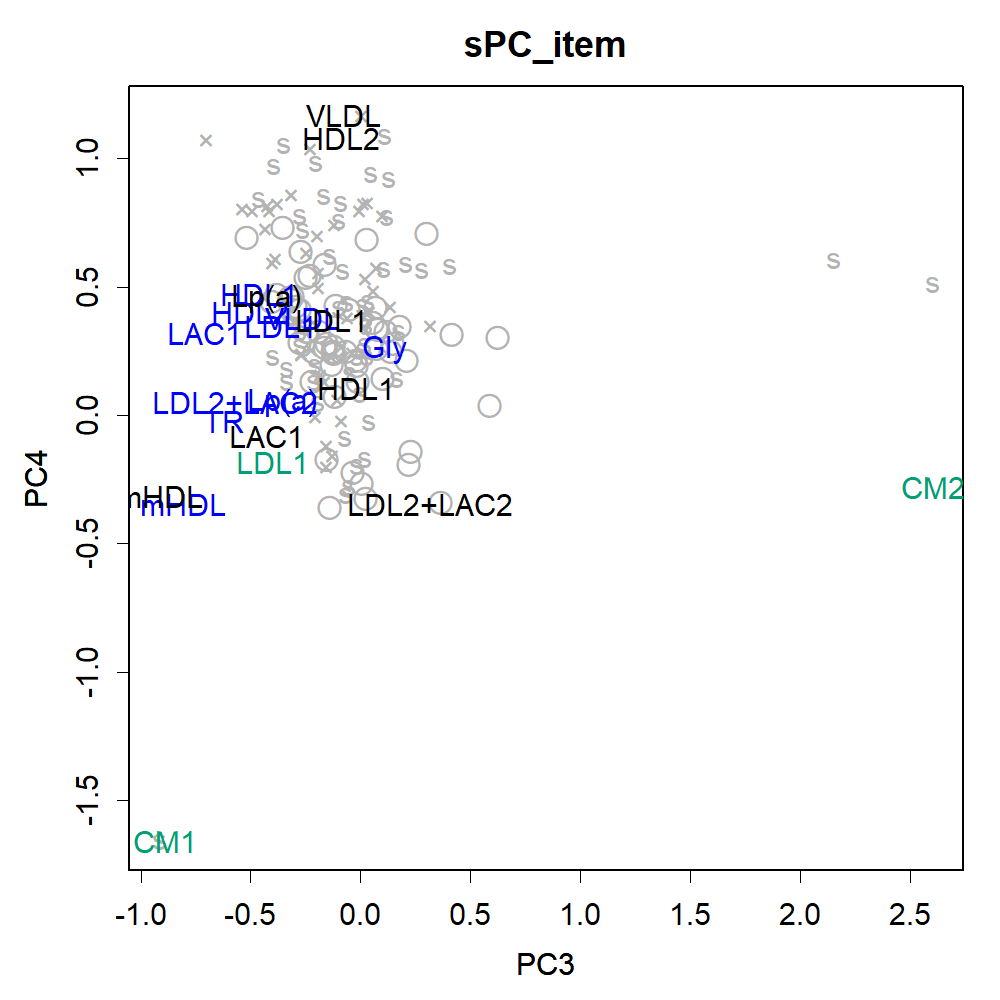

Supplement: S1 File — (ZIP) [file pone.0283855.s001.zip › supplement/PC/PC34item.png]

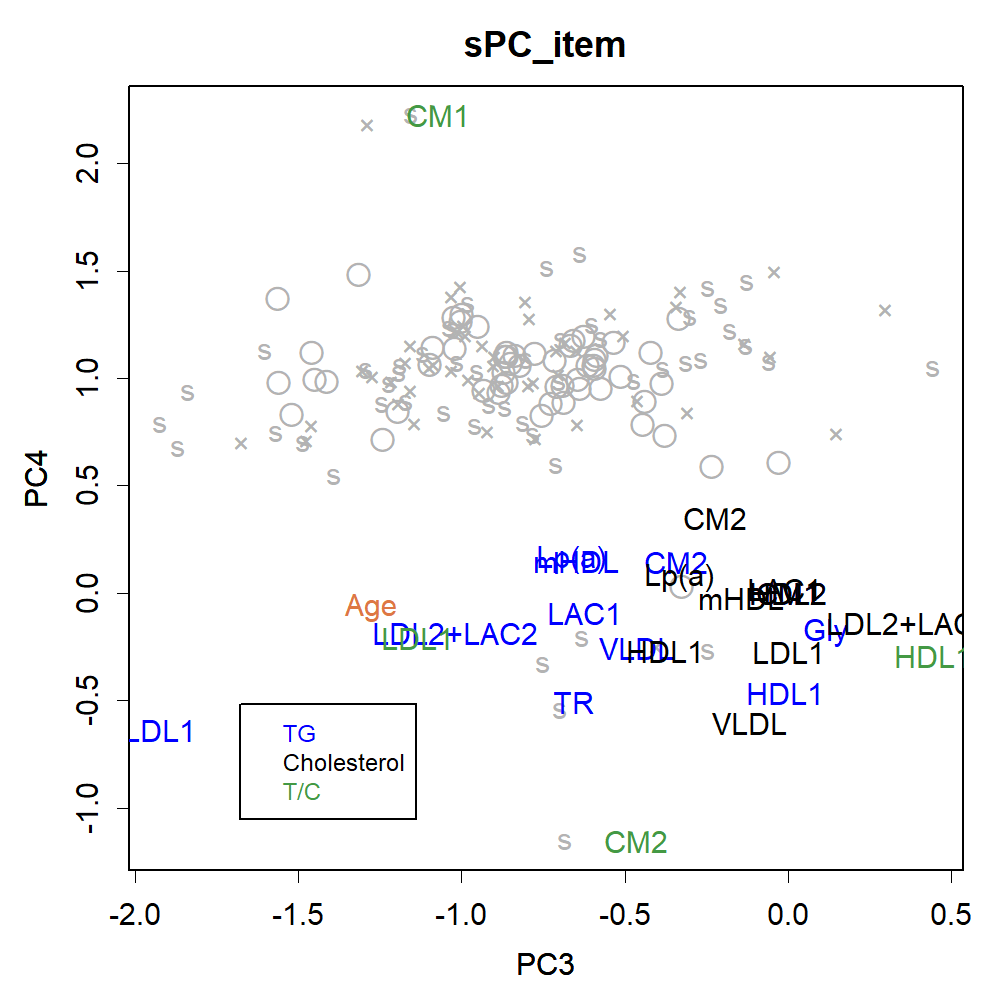

Supplement: S1 File — (ZIP) [file pone.0283855.s001.zip › supplement/PC/PC34itemA.png]

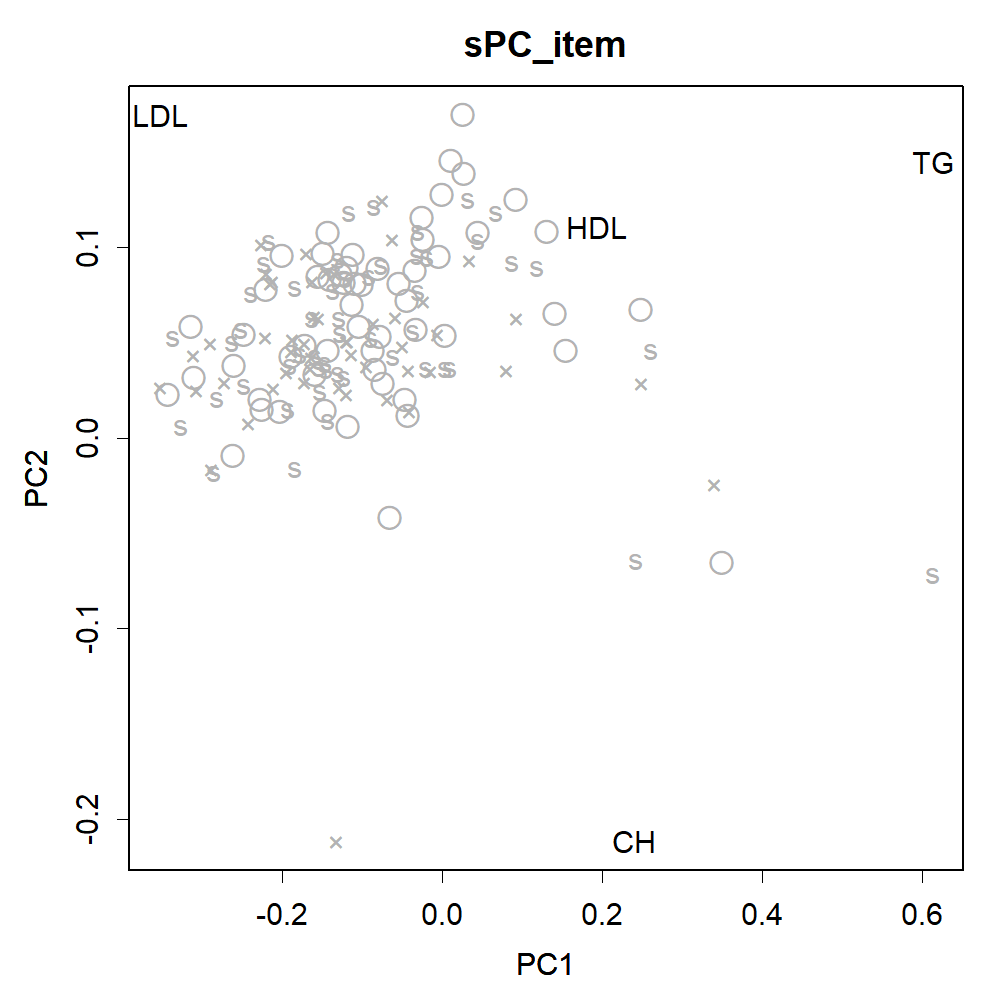

Supplement: S1 File — (ZIP) [file pone.0283855.s001.zip › supplement/PC/PC34itemFurui.png]

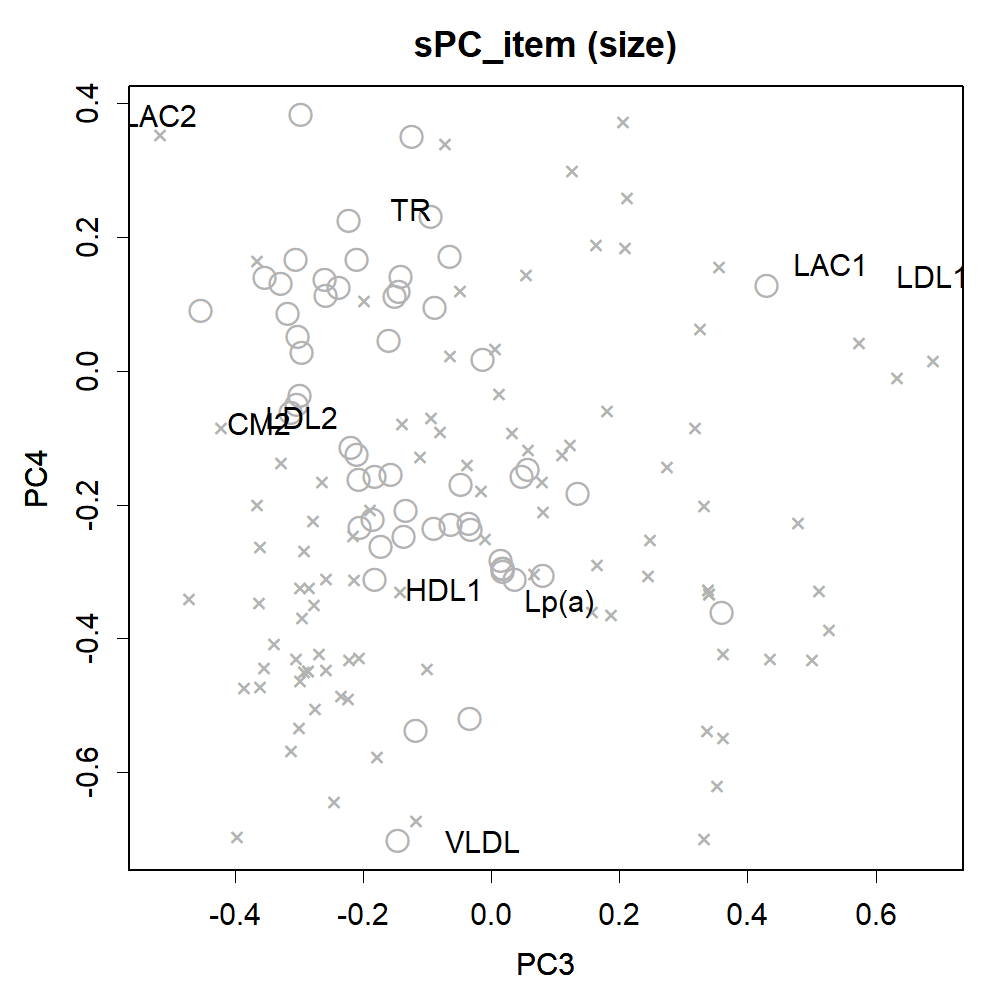

Supplement: S1 File — (ZIP) [file pone.0283855.s001.zip › supplement/PC/PC34itemOOKISA.png]

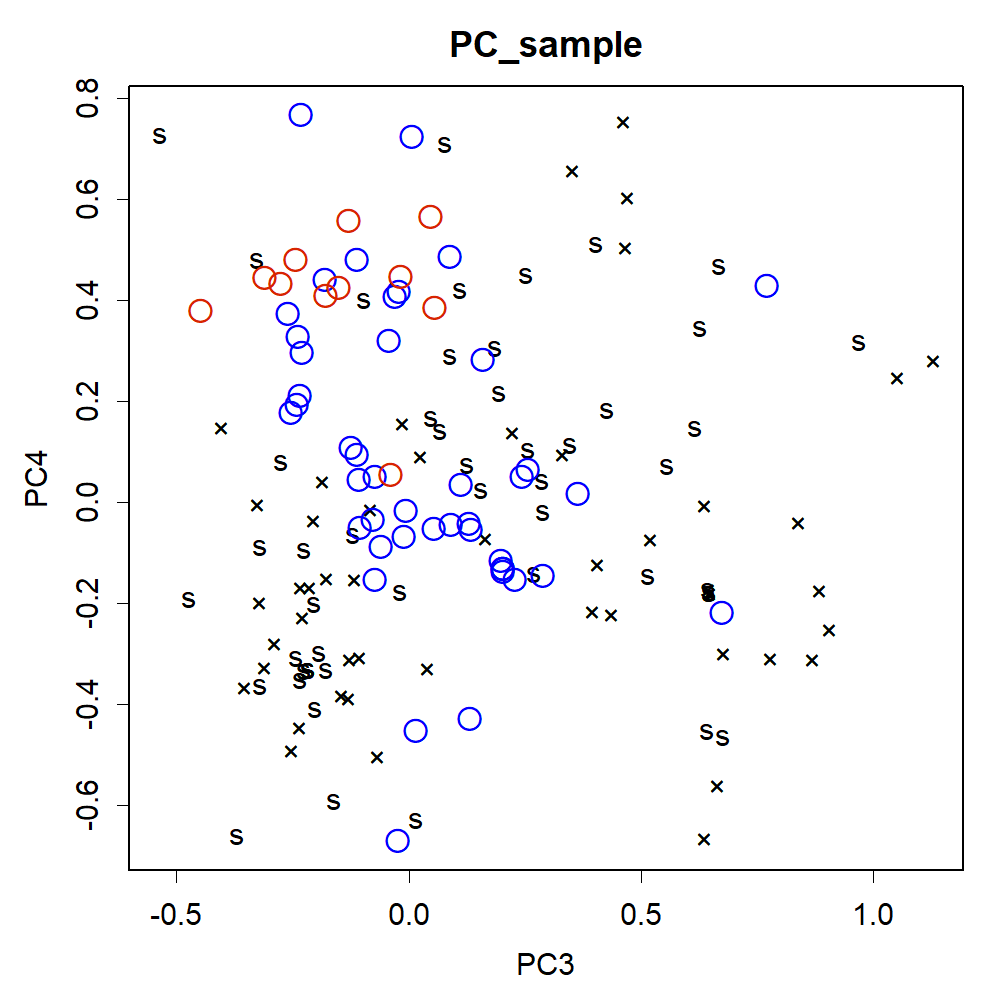

Supplement: S1 File — (ZIP) [file pone.0283855.s001.zip › supplement/PC/PC34ookisa.png]

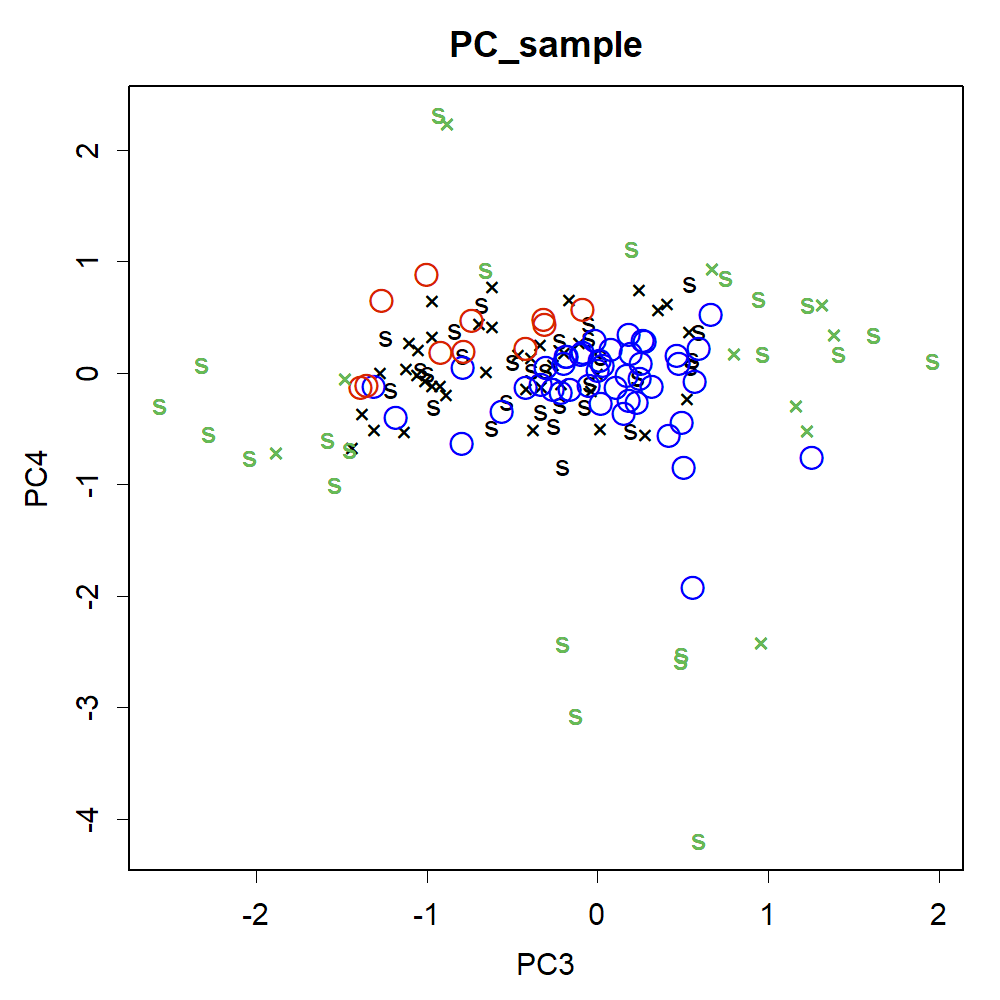

Supplement: S1 File — (ZIP) [file pone.0283855.s001.zip › supplement/PC/PC34orig.png]

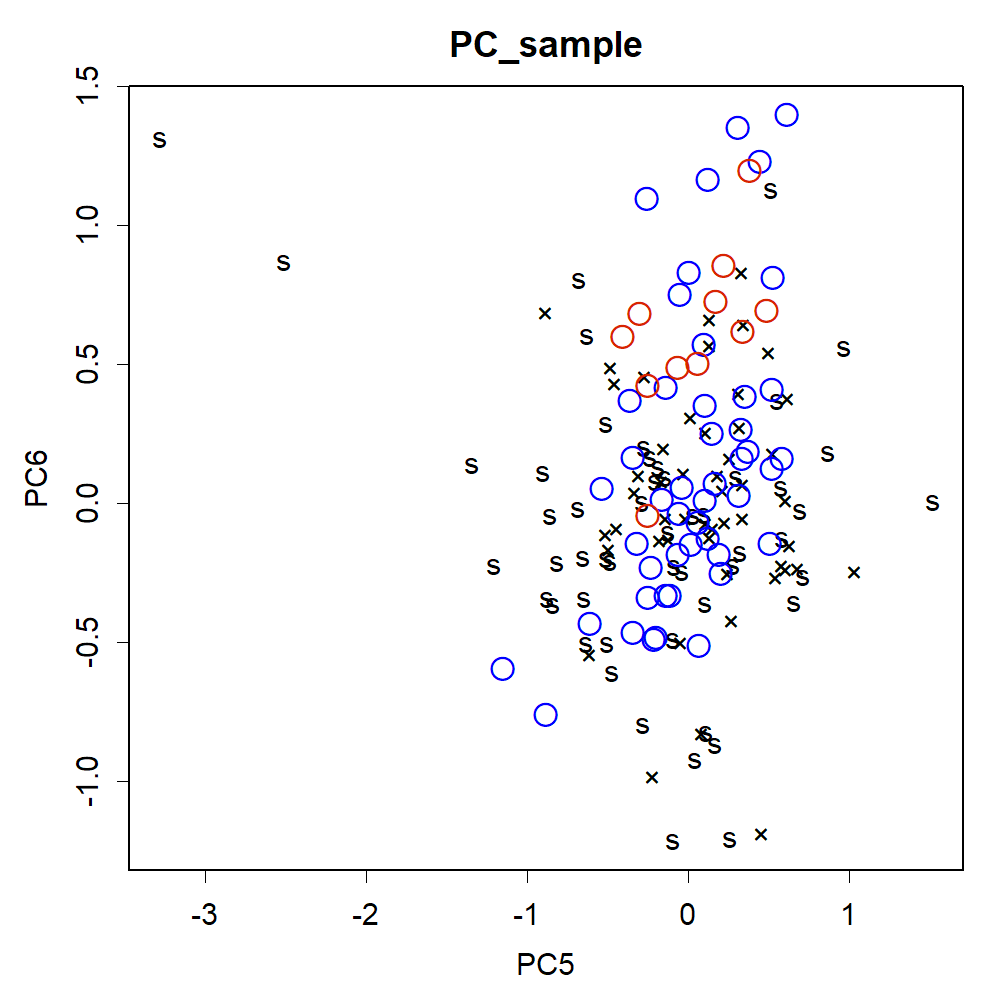

Supplement: S1 File — (ZIP) [file pone.0283855.s001.zip › supplement/PC/PC56.png]

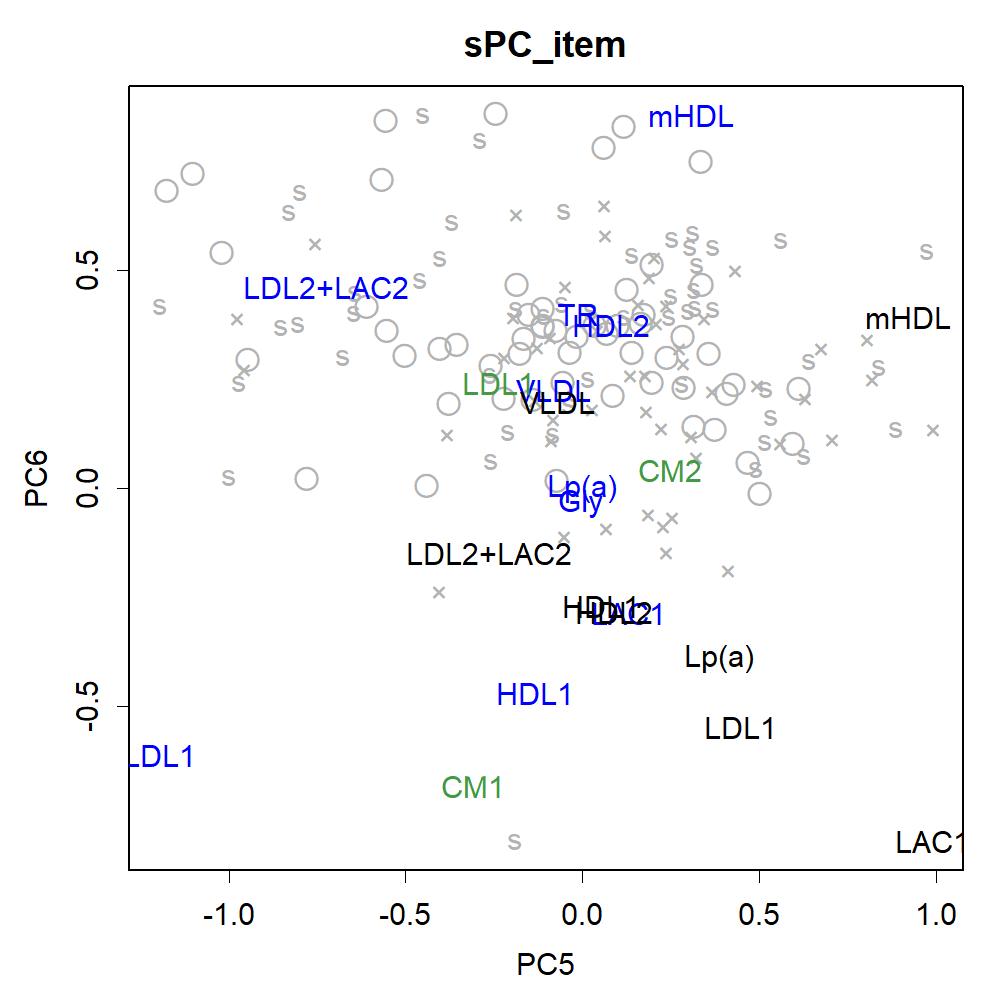

Supplement: S1 File — (ZIP) [file pone.0283855.s001.zip › supplement/PC/PC56item.png]

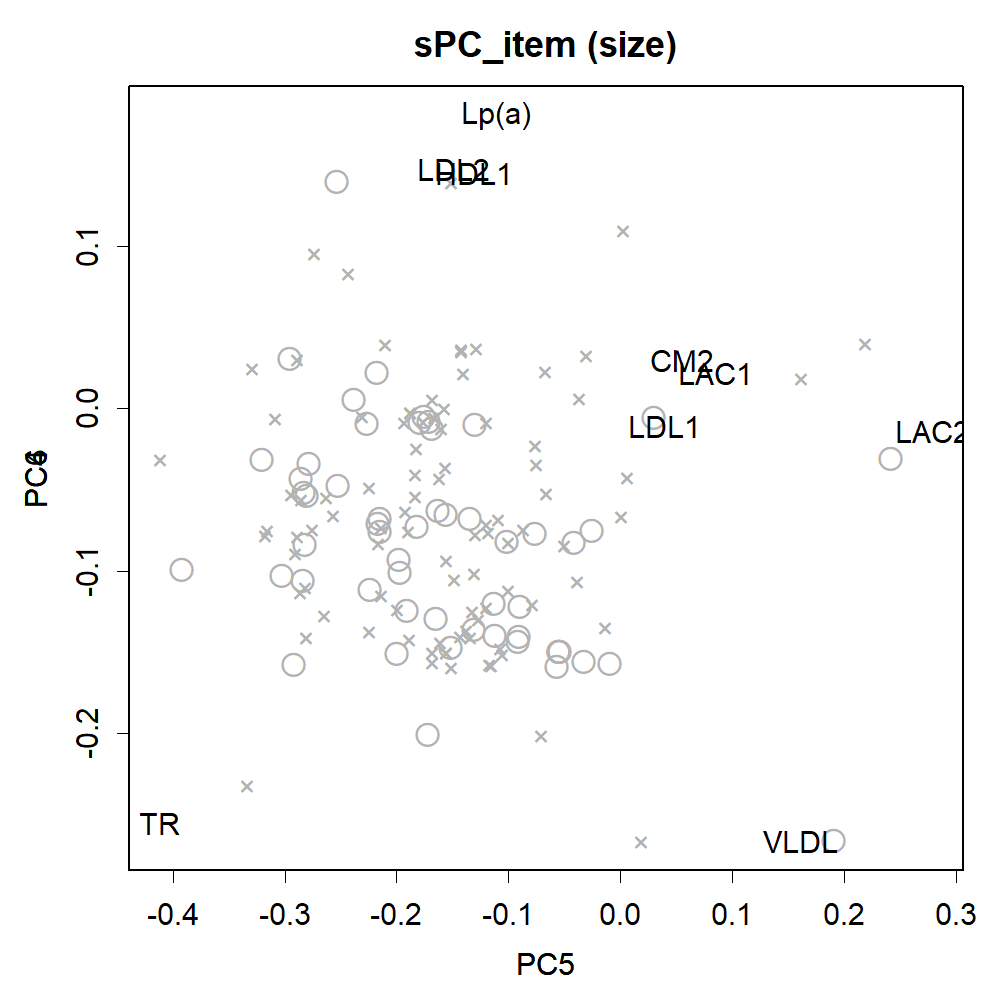

Supplement: S1 File — (ZIP) [file pone.0283855.s001.zip › supplement/PC/PC56itemOOKISA.png]

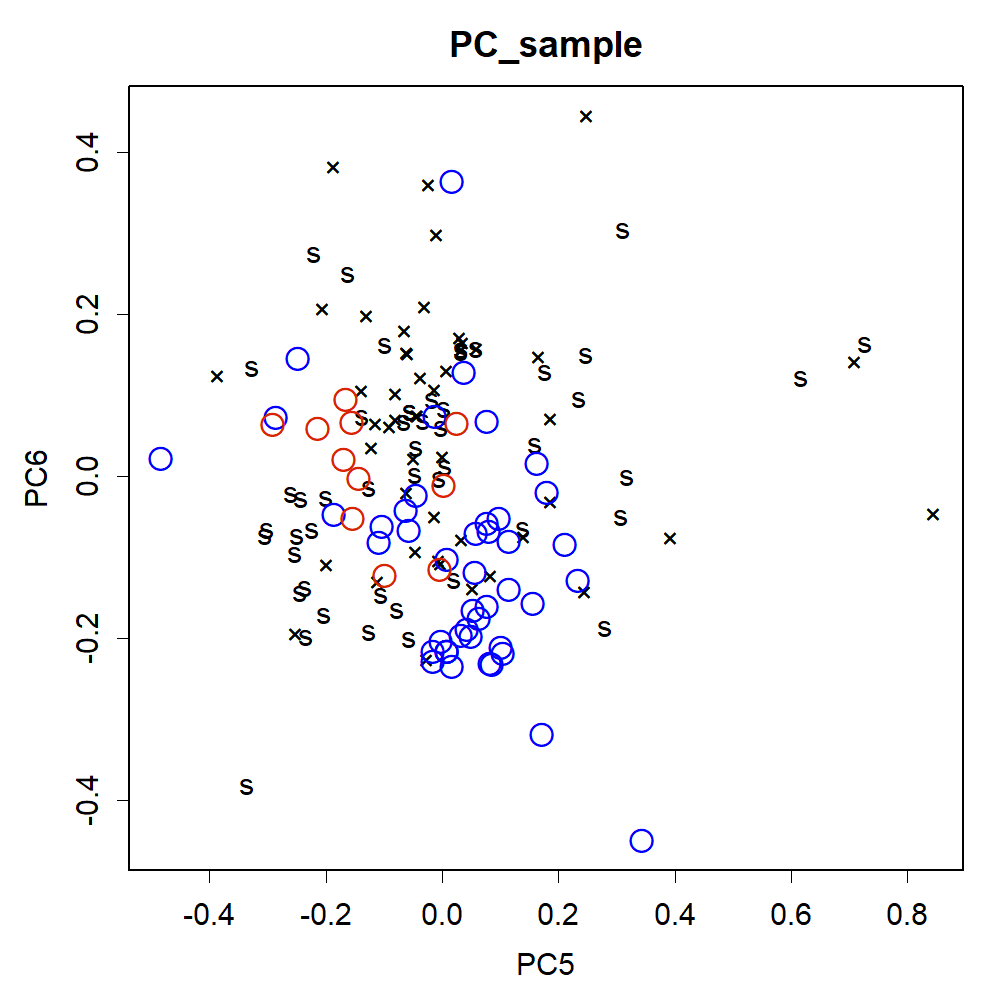

Supplement: S1 File — (ZIP) [file pone.0283855.s001.zip › supplement/PC/PC56ookisa.png]

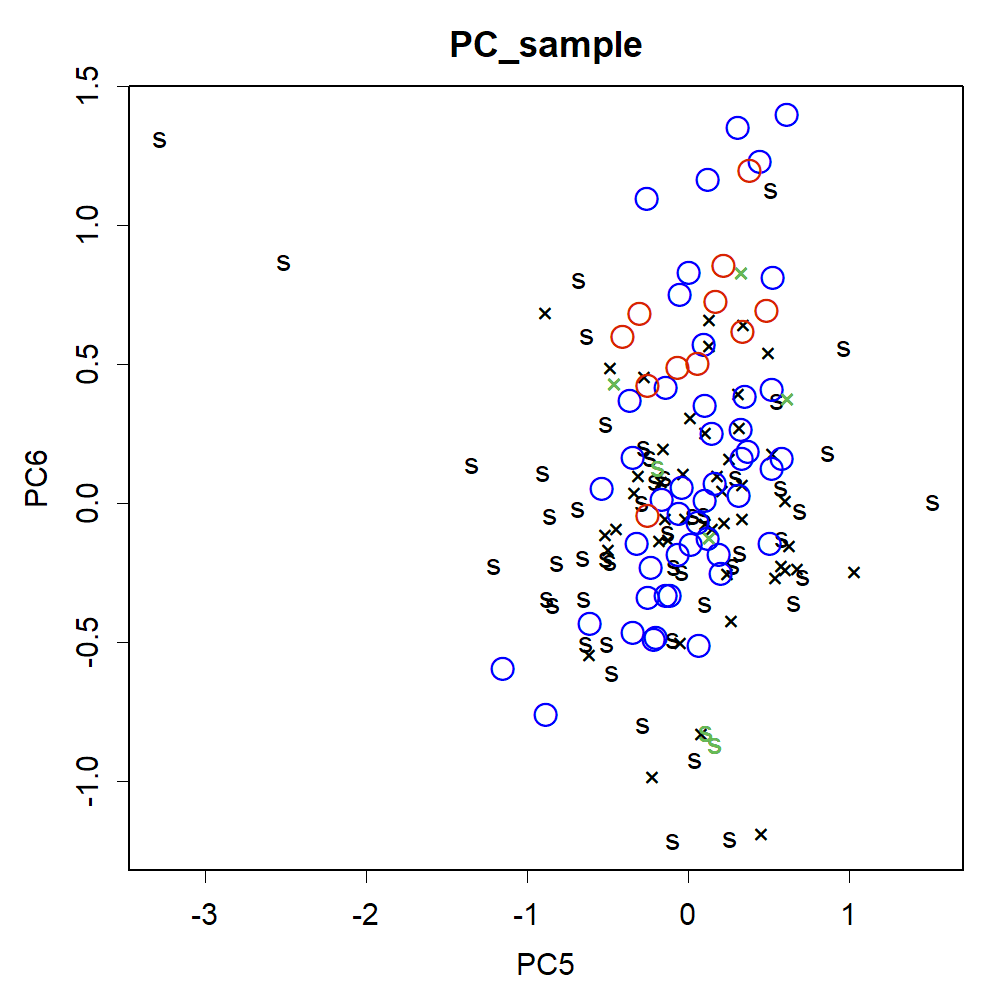

Supplement: S1 File — (ZIP) [file pone.0283855.s001.zip › supplement/PC/PC56Orig.png]

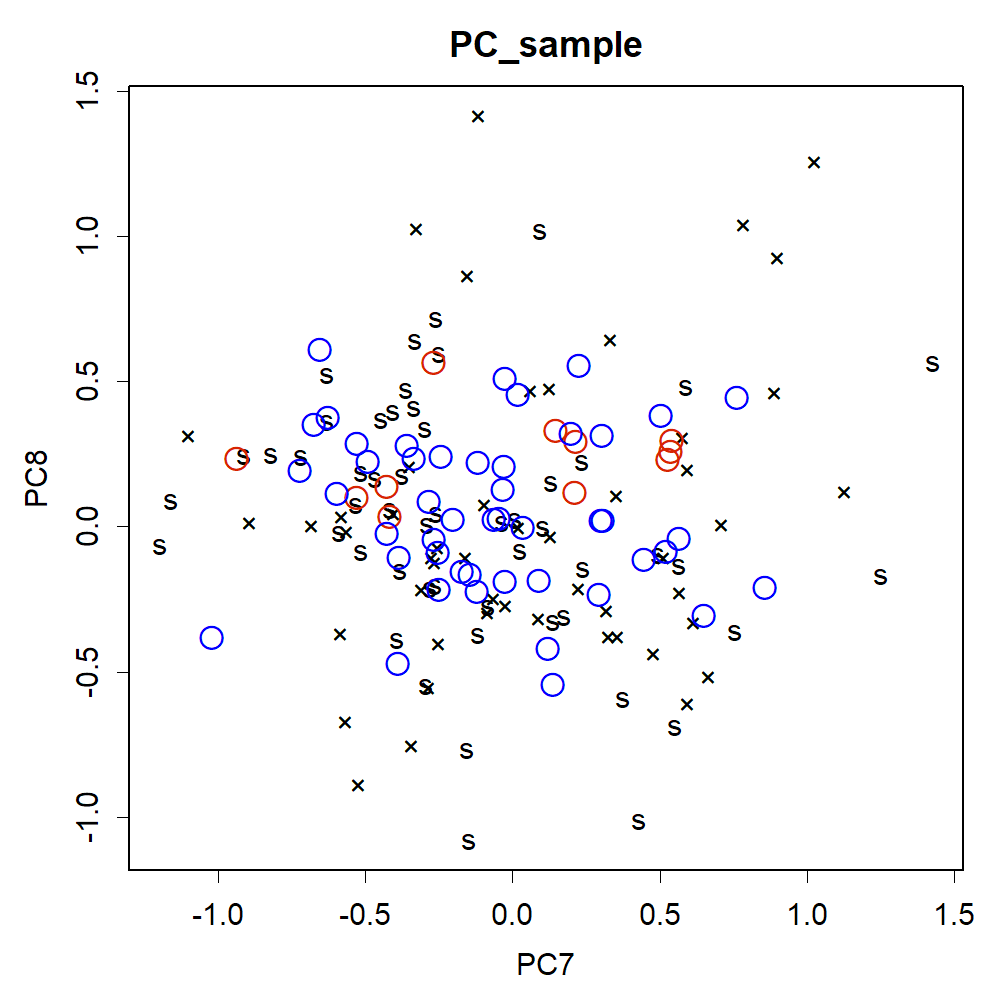

Supplement: S1 File — (ZIP) [file pone.0283855.s001.zip › supplement/PC/PC78.png]

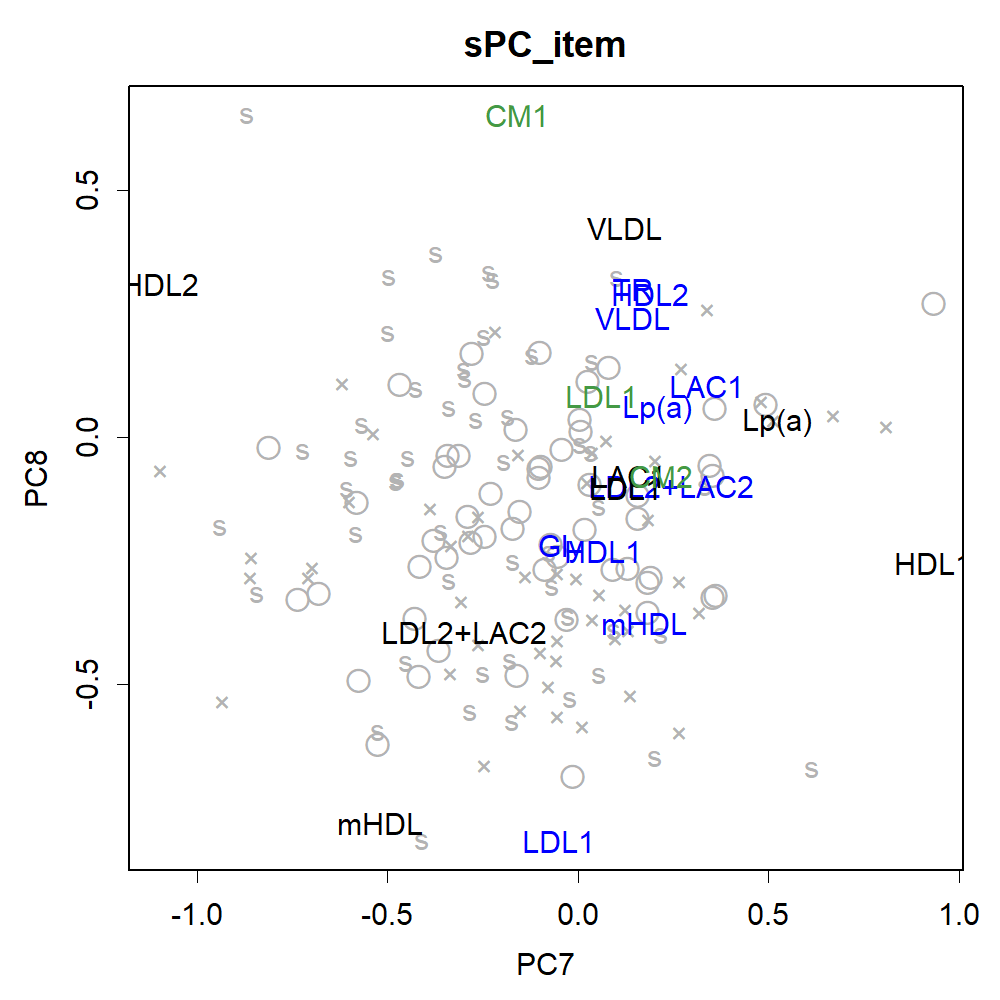

Supplement: S1 File — (ZIP) [file pone.0283855.s001.zip › supplement/PC/PC78item.png]

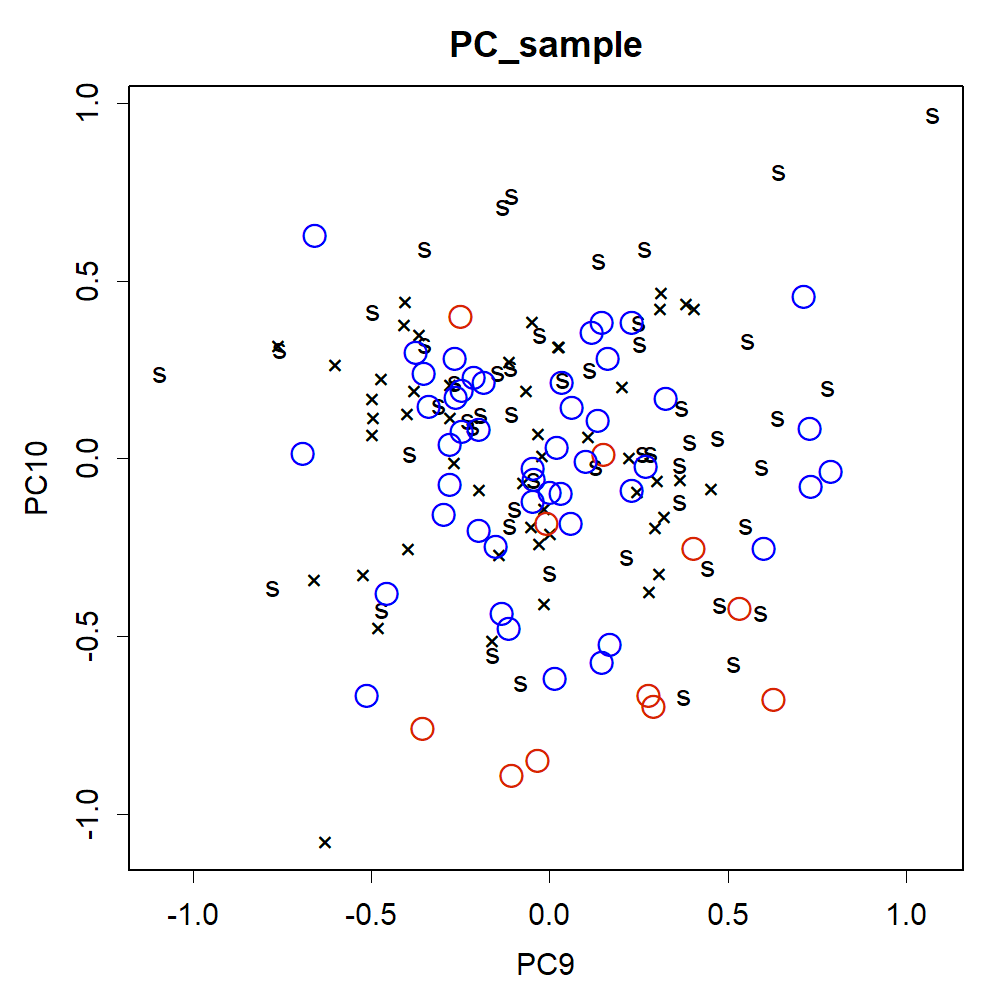

Supplement: S1 File — (ZIP) [file pone.0283855.s001.zip › supplement/PC/PC910.png]

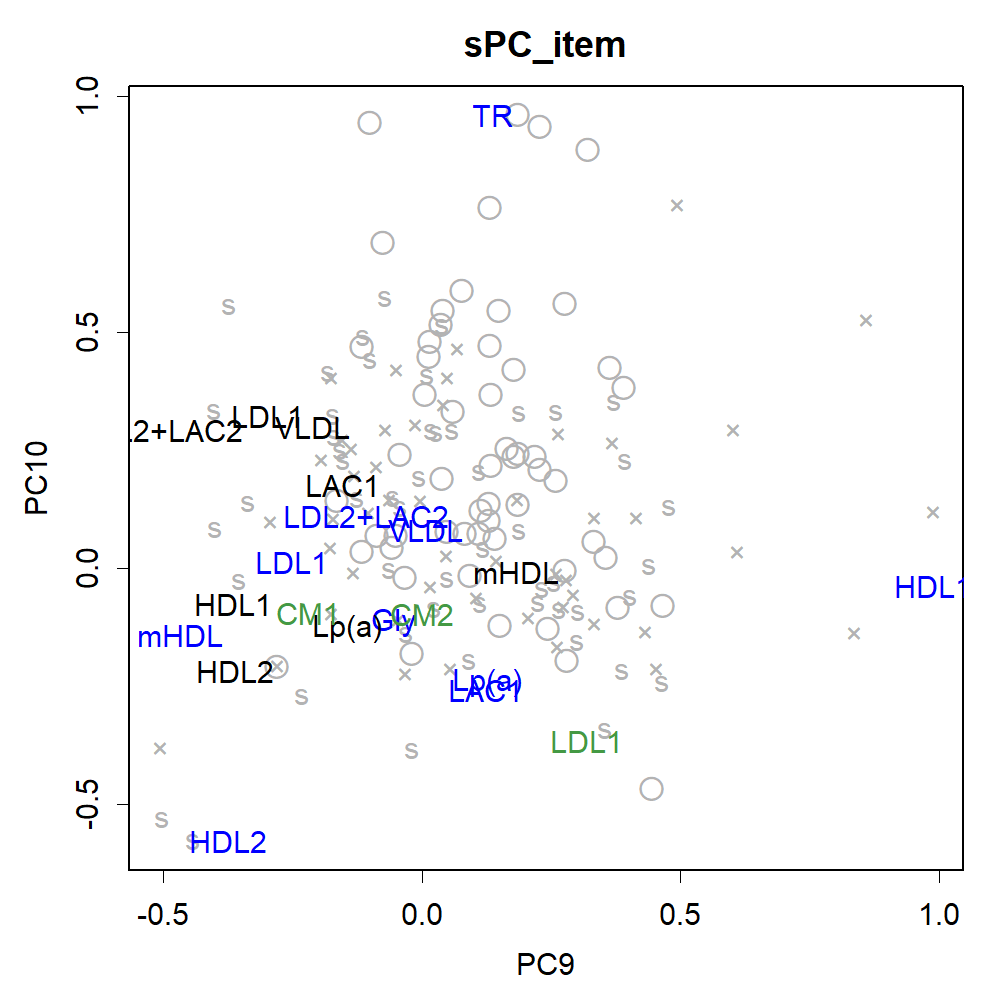

Supplement: S1 File — (ZIP) [file pone.0283855.s001.zip › supplement/PC/PC910item.png]

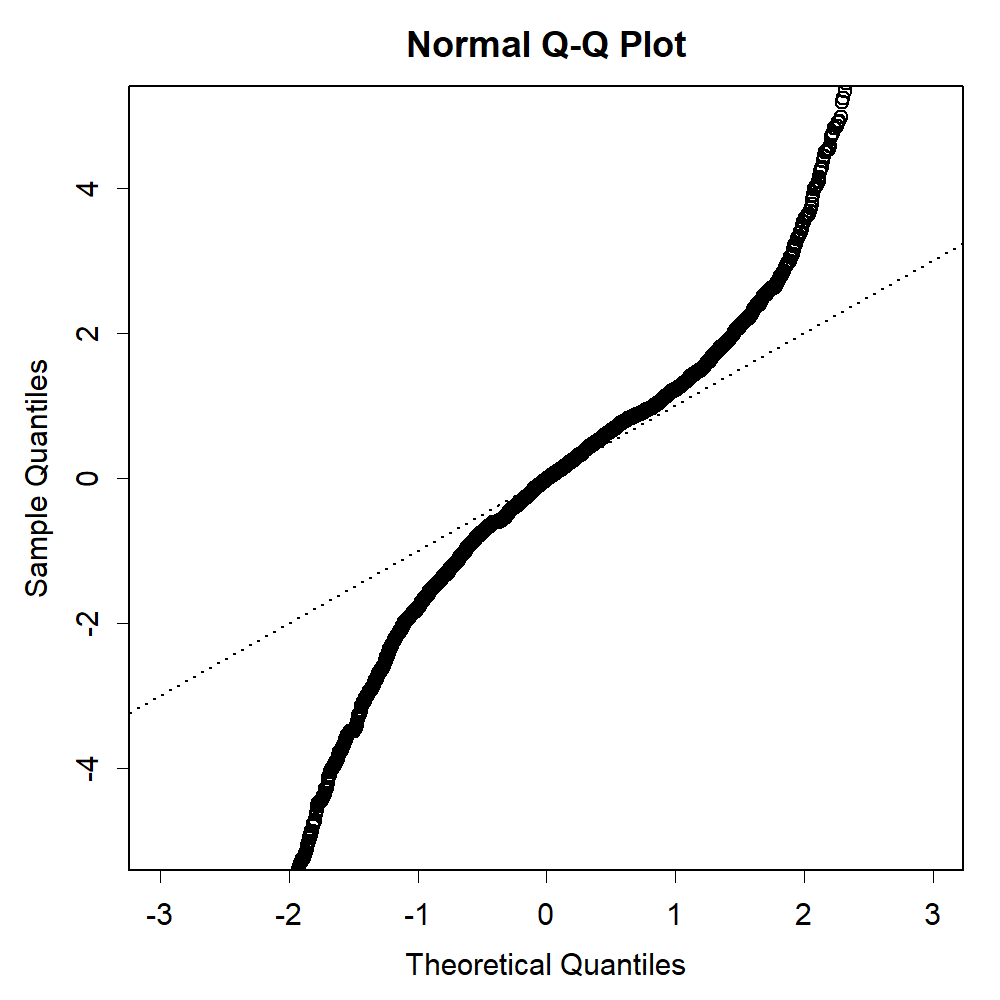

Supplement: S1 File — (ZIP) [file pone.0283855.s001.zip › supplement/PC/QQ.png]

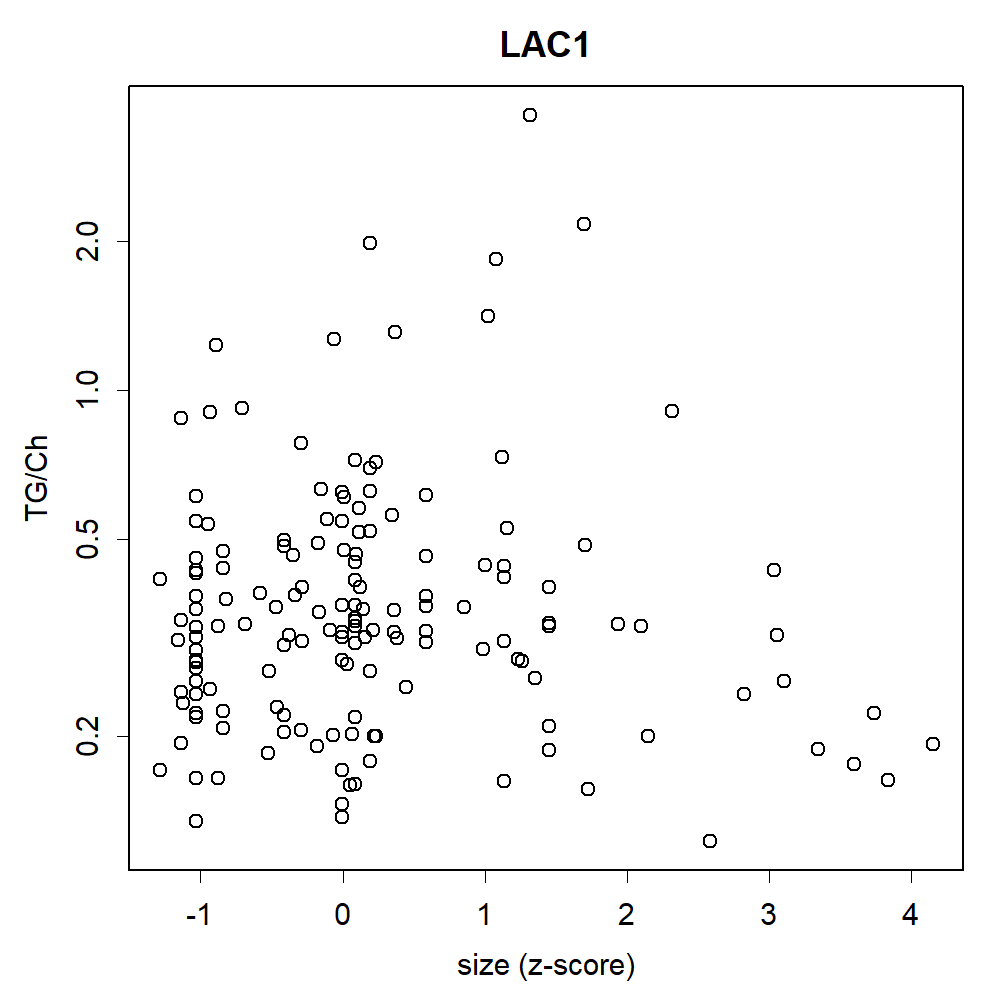

Supplement: S1 File — (ZIP) [file pone.0283855.s001.zip › supplement/PC/sizetocontentLAC1.png]

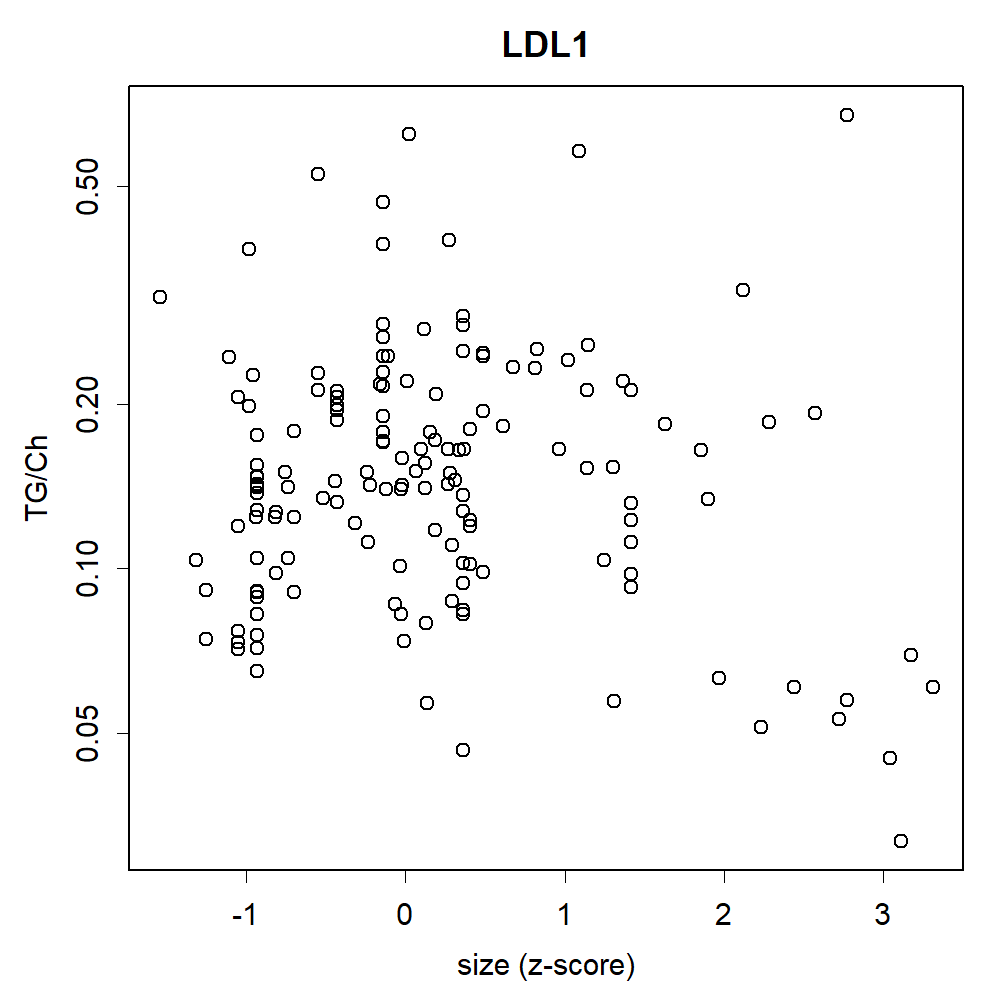

Supplement: S1 File — (ZIP) [file pone.0283855.s001.zip › supplement/PC/sizetocontentLDL1.png]

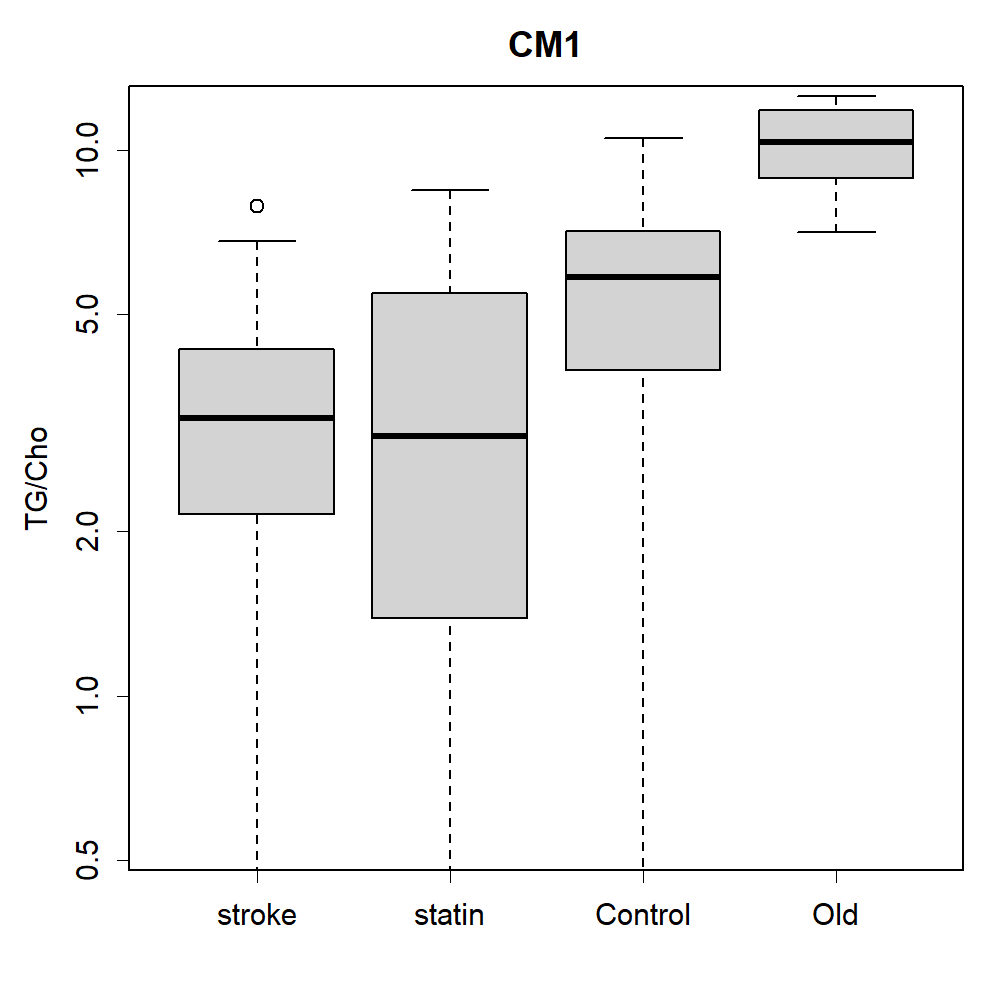

Supplement: S1 File — (ZIP) [file pone.0283855.s001.zip › supplement/TC/CM1.png]

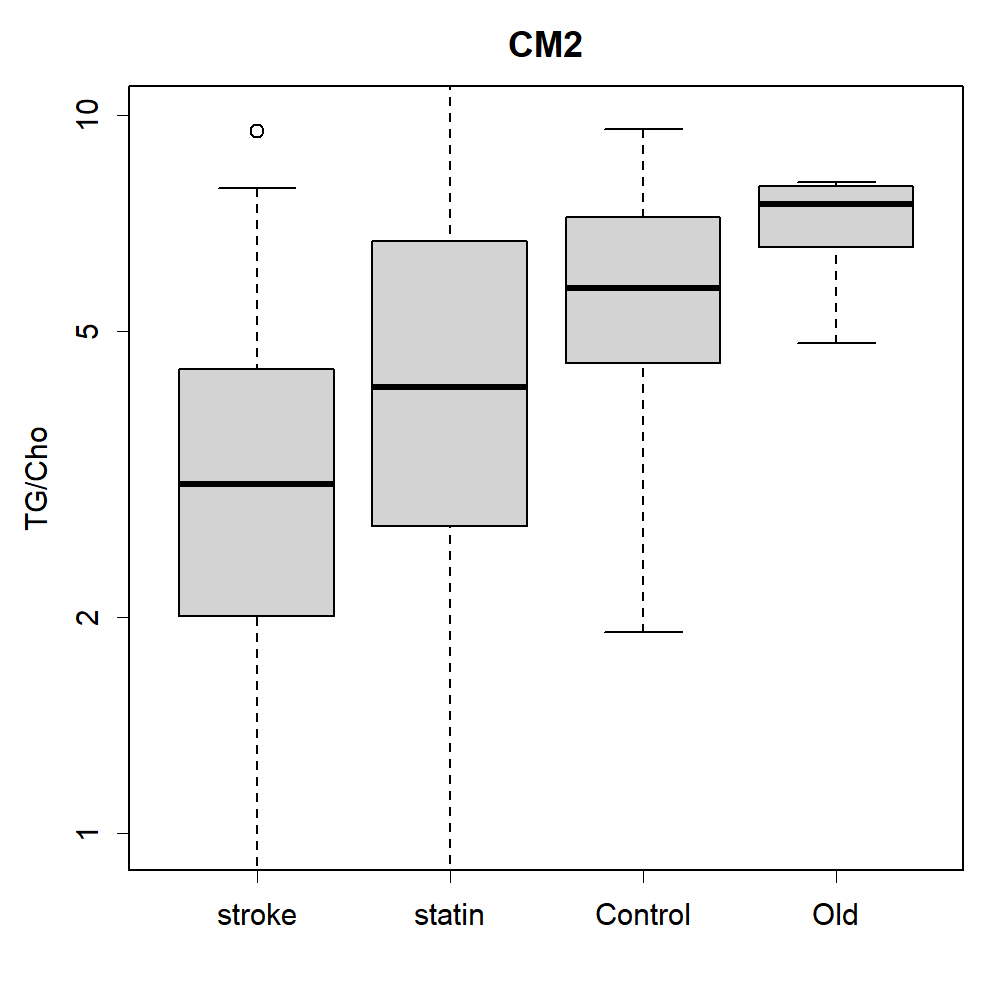

Supplement: S1 File — (ZIP) [file pone.0283855.s001.zip › supplement/TC/CM2.png]

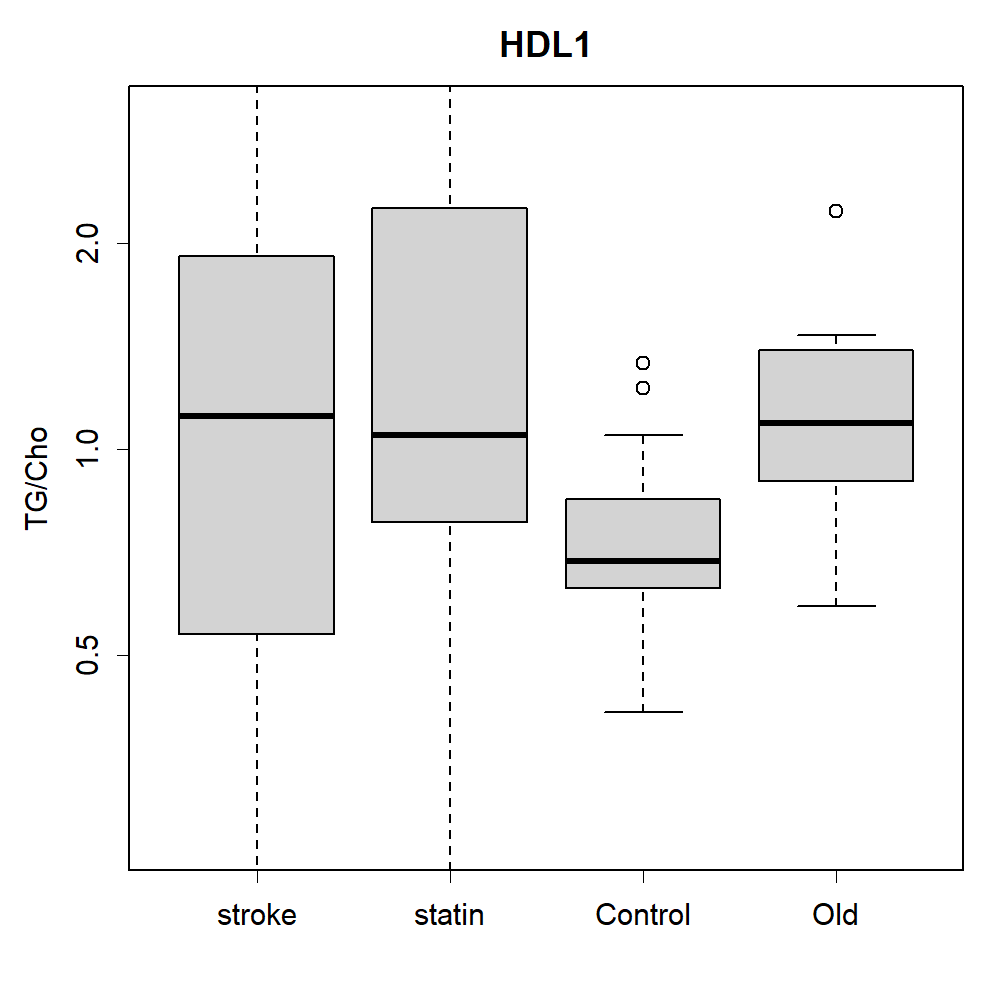

Supplement: S1 File — (ZIP) [file pone.0283855.s001.zip › supplement/TC/HDL1.png]

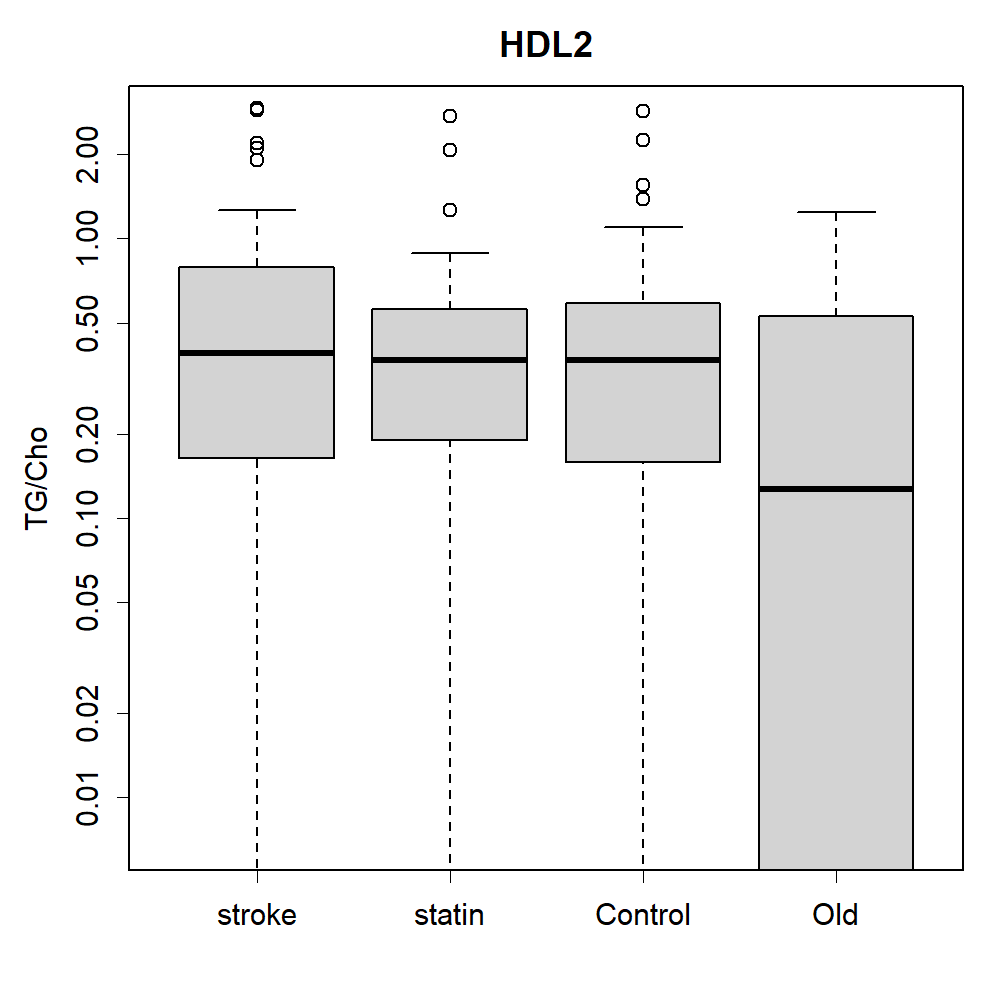

Supplement: S1 File — (ZIP) [file pone.0283855.s001.zip › supplement/TC/HDL2.png]

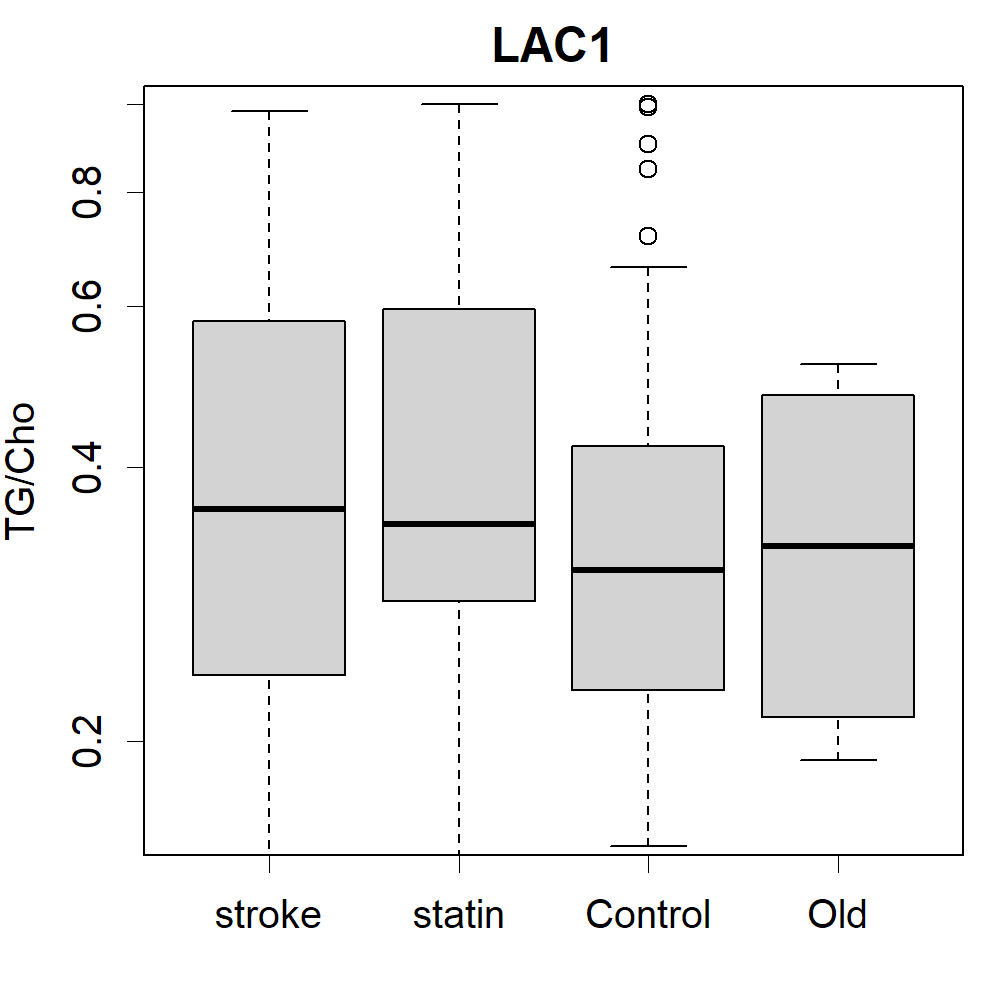

Supplement: S1 File — (ZIP) [file pone.0283855.s001.zip › supplement/TC/LAC1.png]

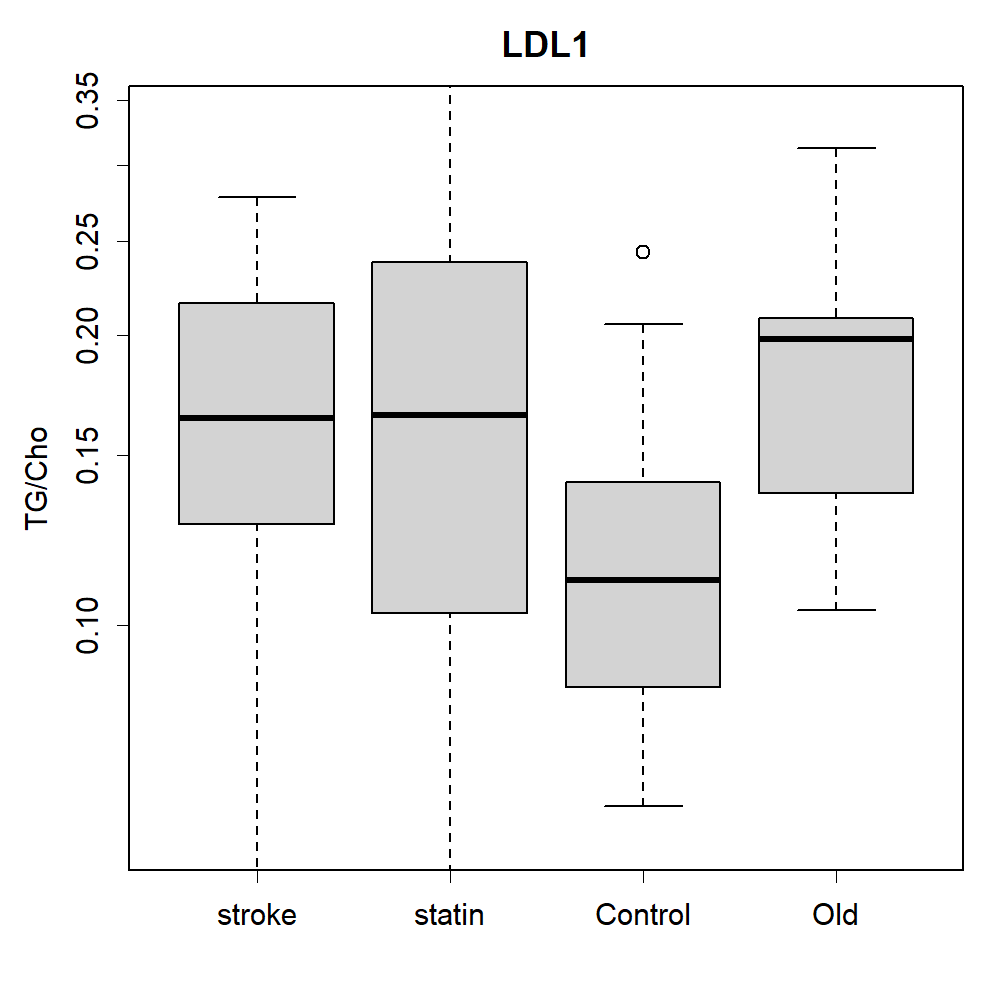

Supplement: S1 File — (ZIP) [file pone.0283855.s001.zip › supplement/TC/LDL1.png]

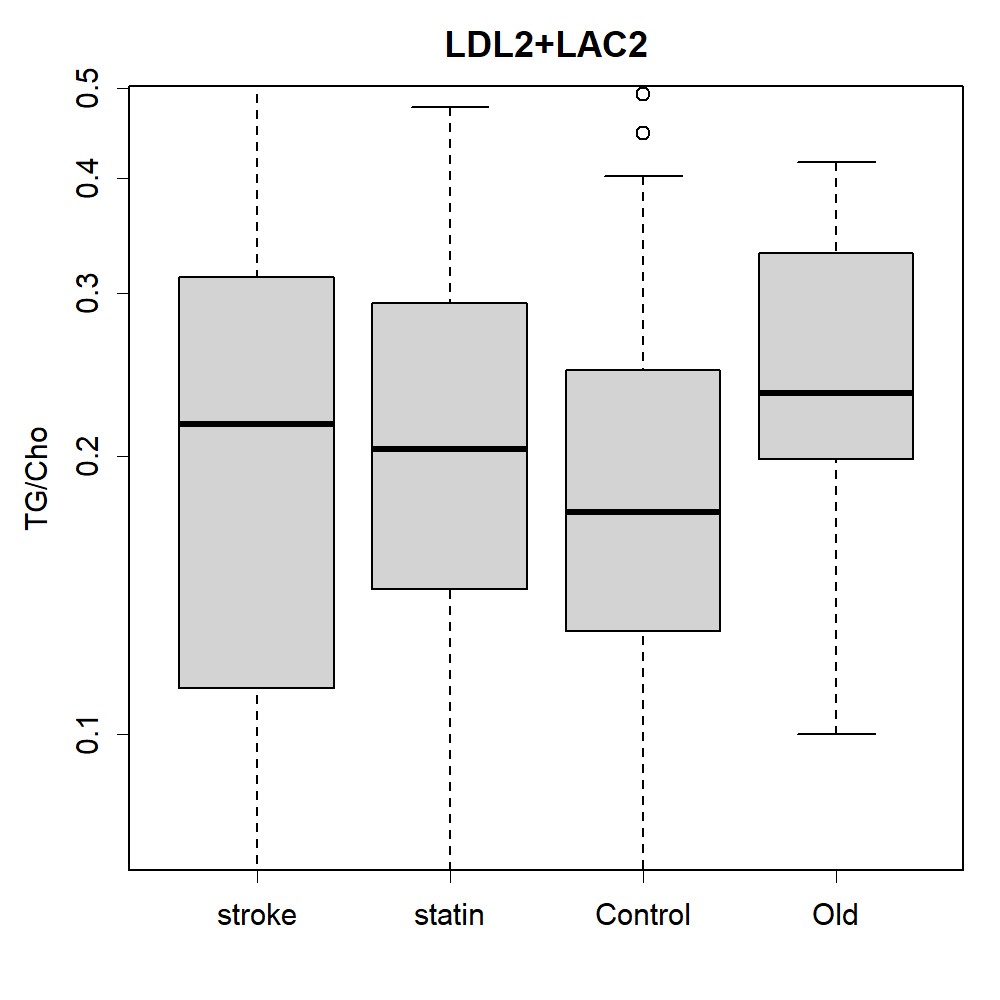

Supplement: S1 File — (ZIP) [file pone.0283855.s001.zip › supplement/TC/LDL2+LAC2.png]

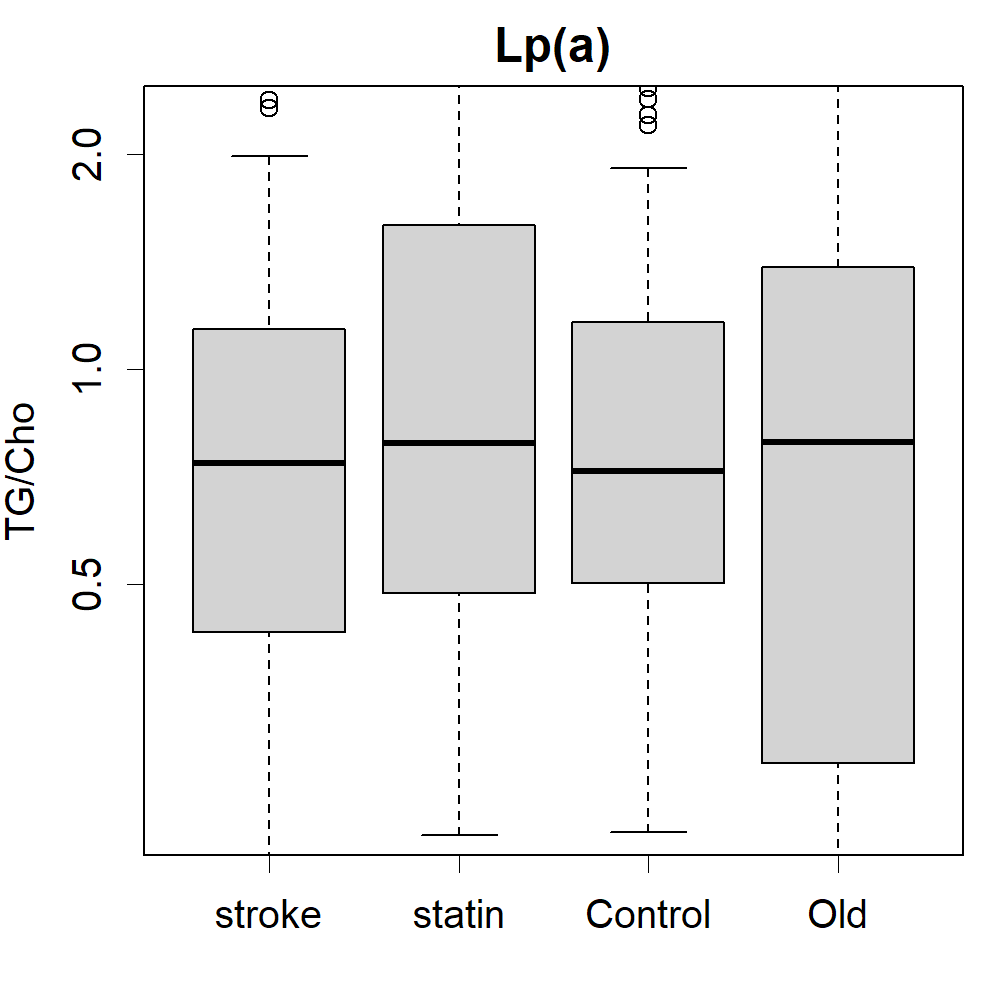

Supplement: S1 File — (ZIP) [file pone.0283855.s001.zip › supplement/TC/Lp(a).png]

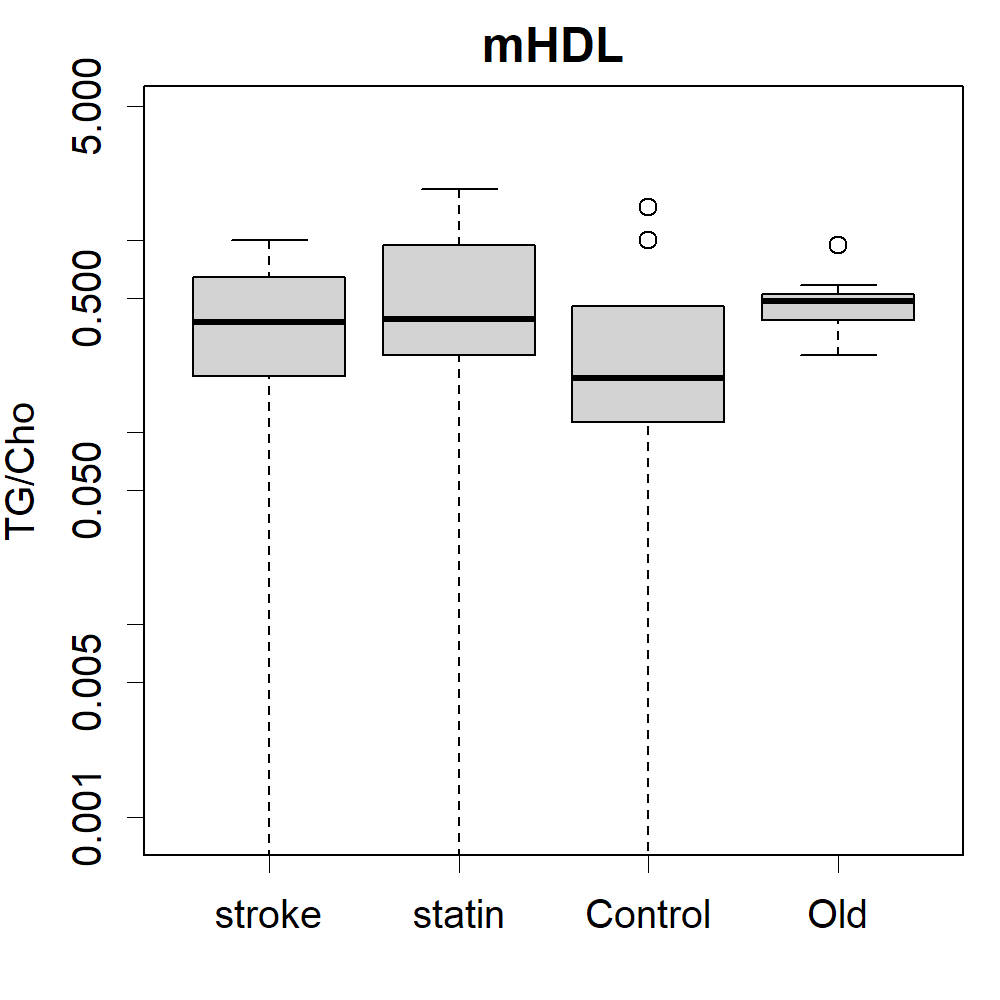

Supplement: S1 File — (ZIP) [file pone.0283855.s001.zip › supplement/TC/mHDL.png]

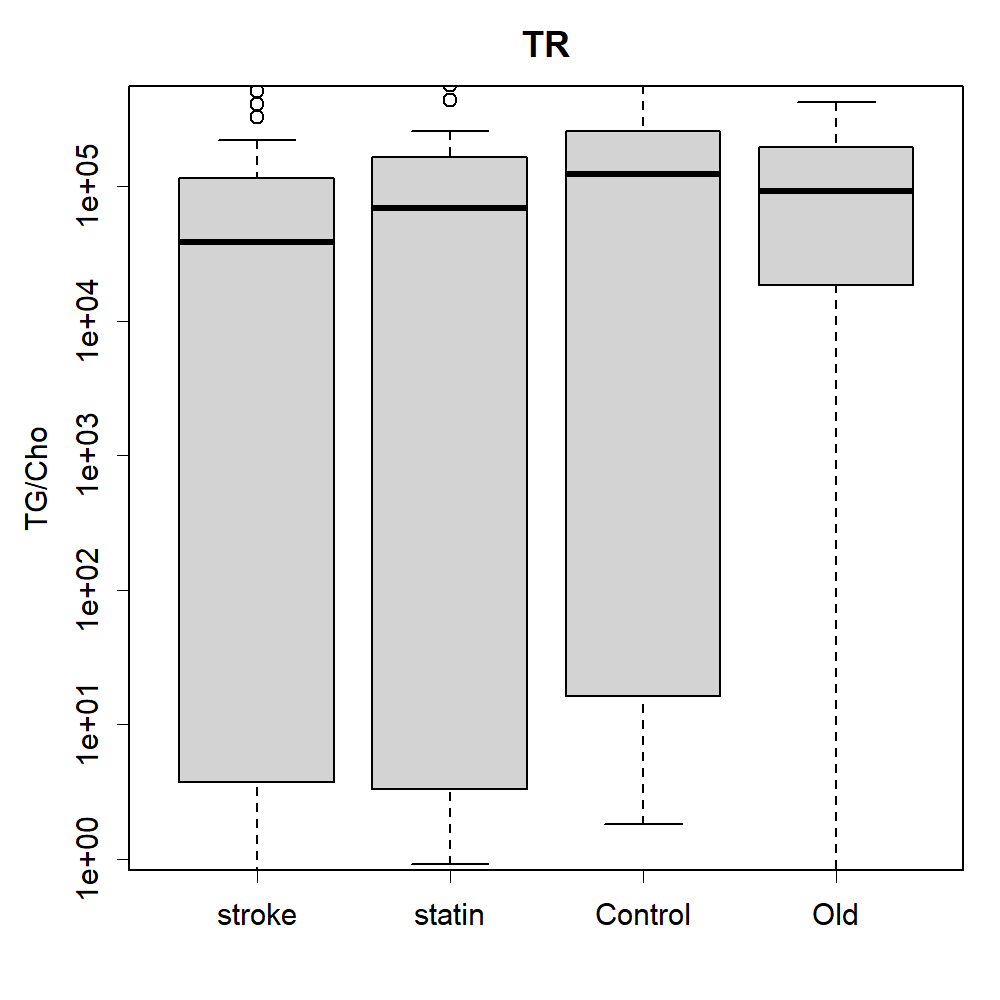

Supplement: S1 File — (ZIP) [file pone.0283855.s001.zip › supplement/TC/TR.png]

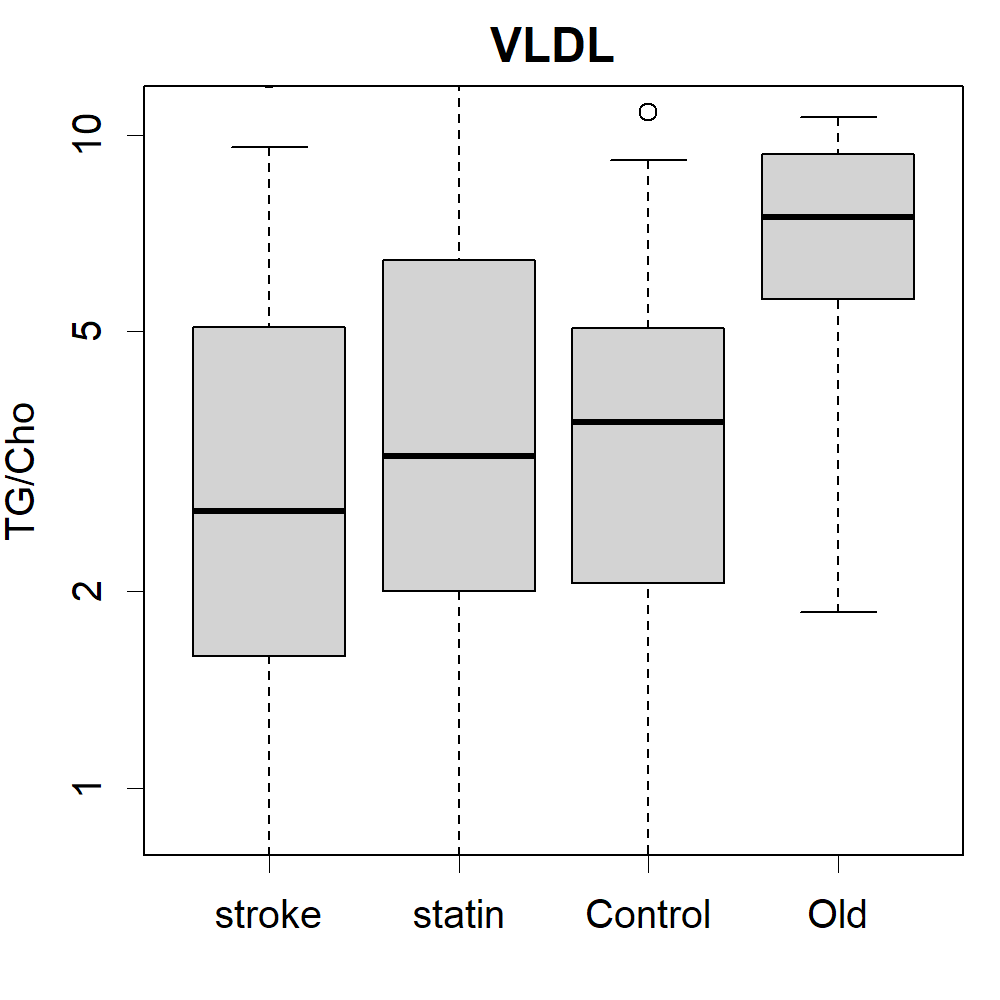

Supplement: S1 File — (ZIP) [file pone.0283855.s001.zip › supplement/TC/VLDL.png]

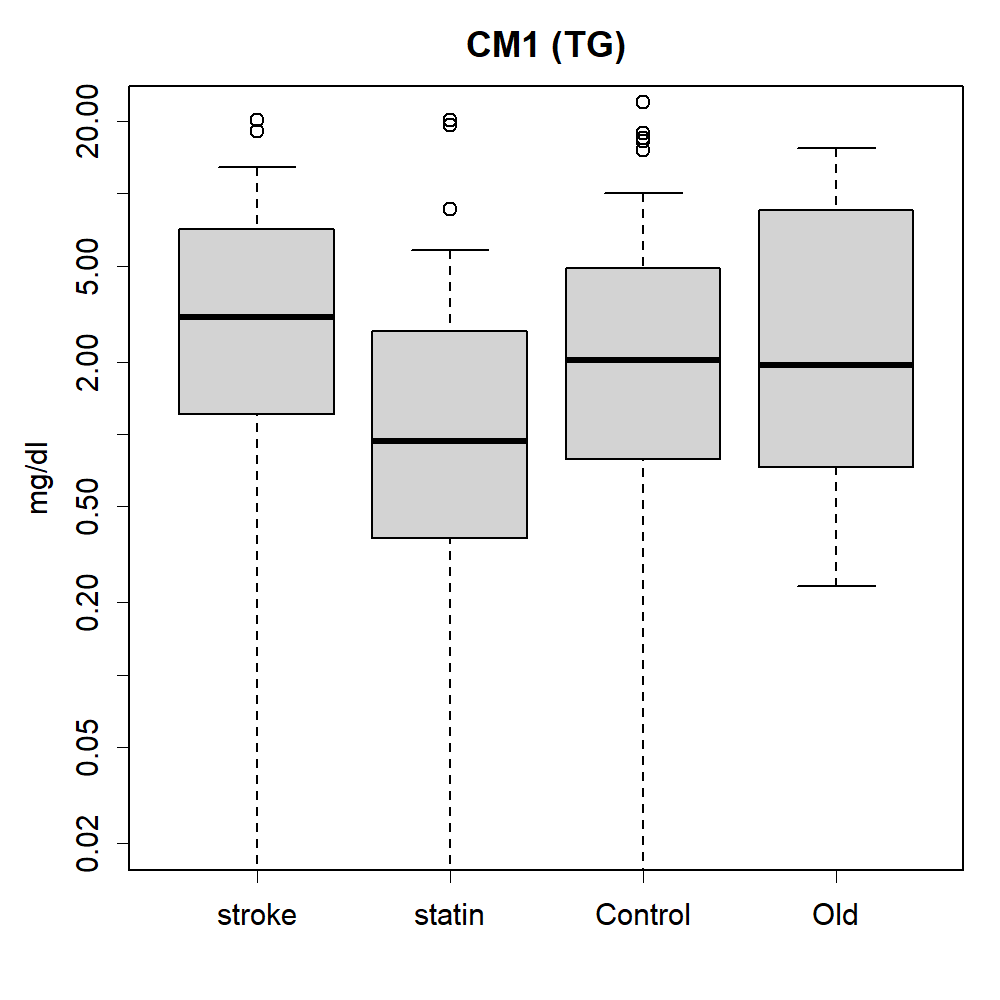

Supplement: S1 File — (ZIP) [file pone.0283855.s001.zip › supplement/TG/CM1.png]

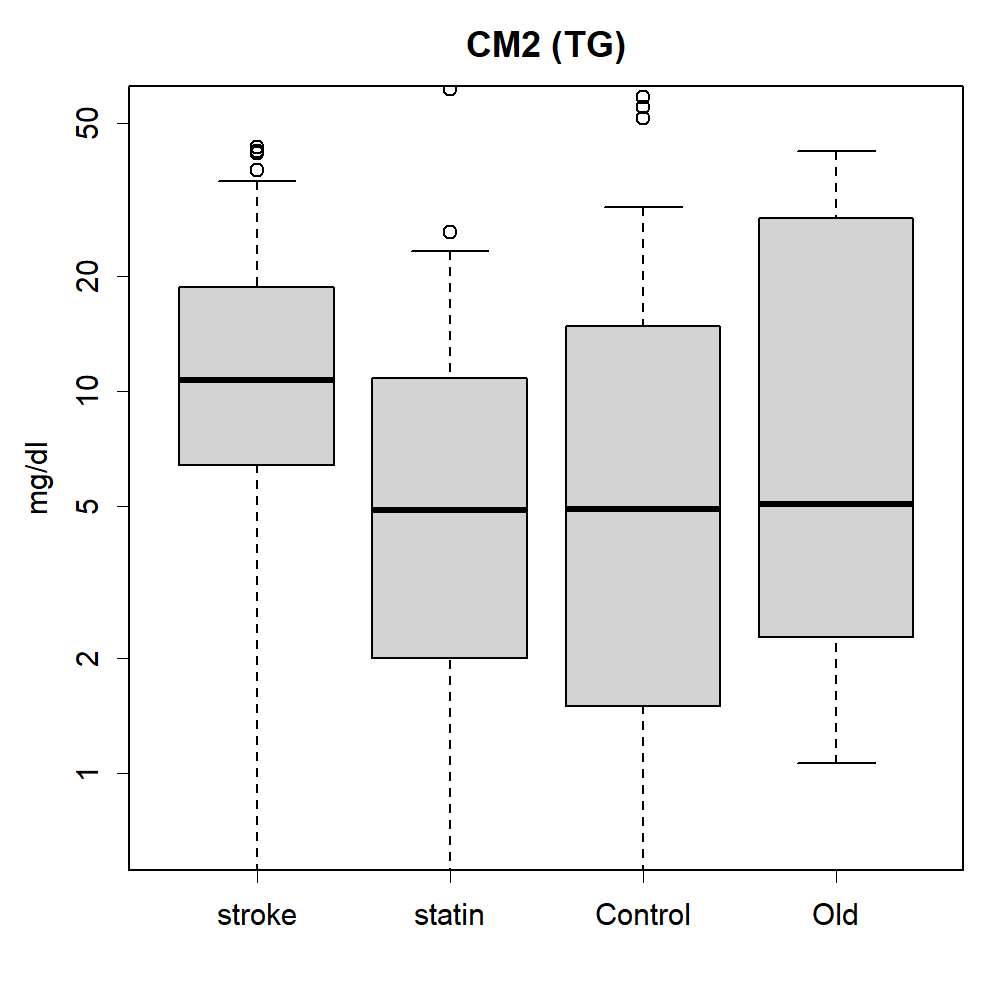

Supplement: S1 File — (ZIP) [file pone.0283855.s001.zip › supplement/TG/CM2.png]

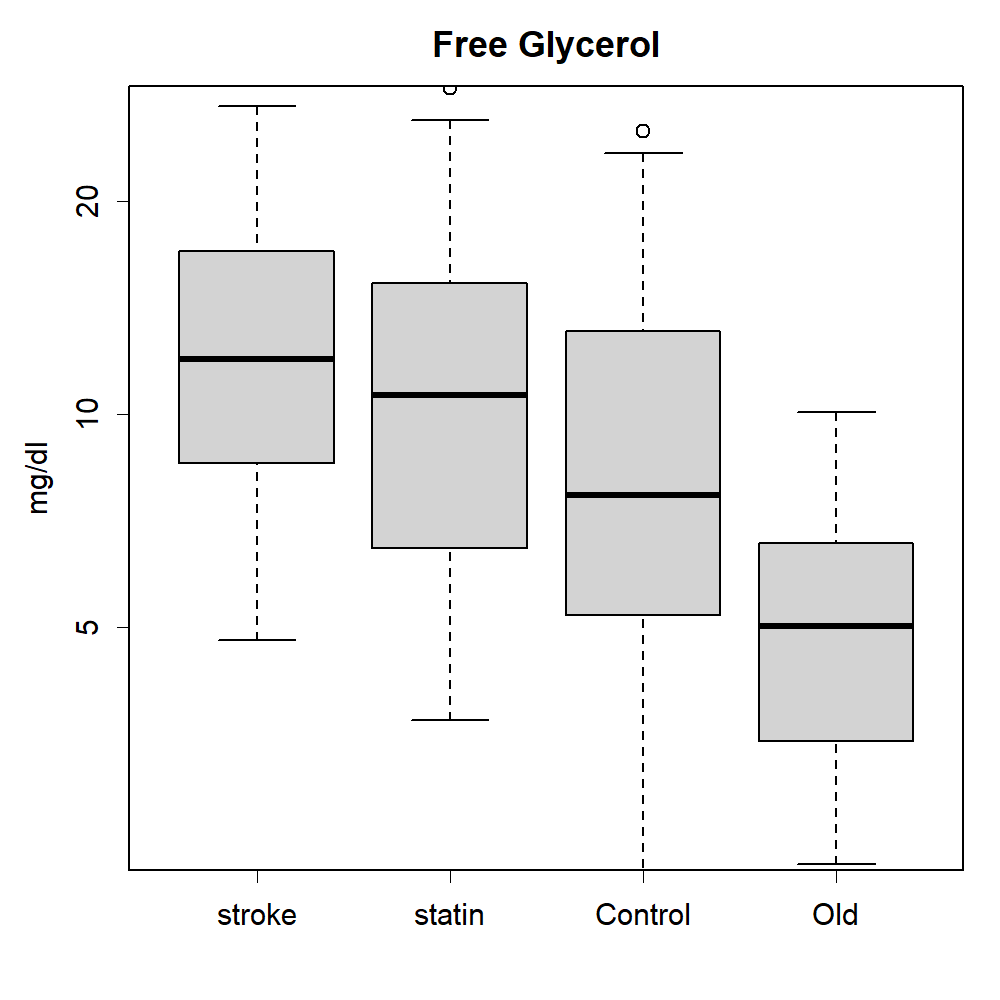

Supplement: S1 File — (ZIP) [file pone.0283855.s001.zip › supplement/TG/gly.png]

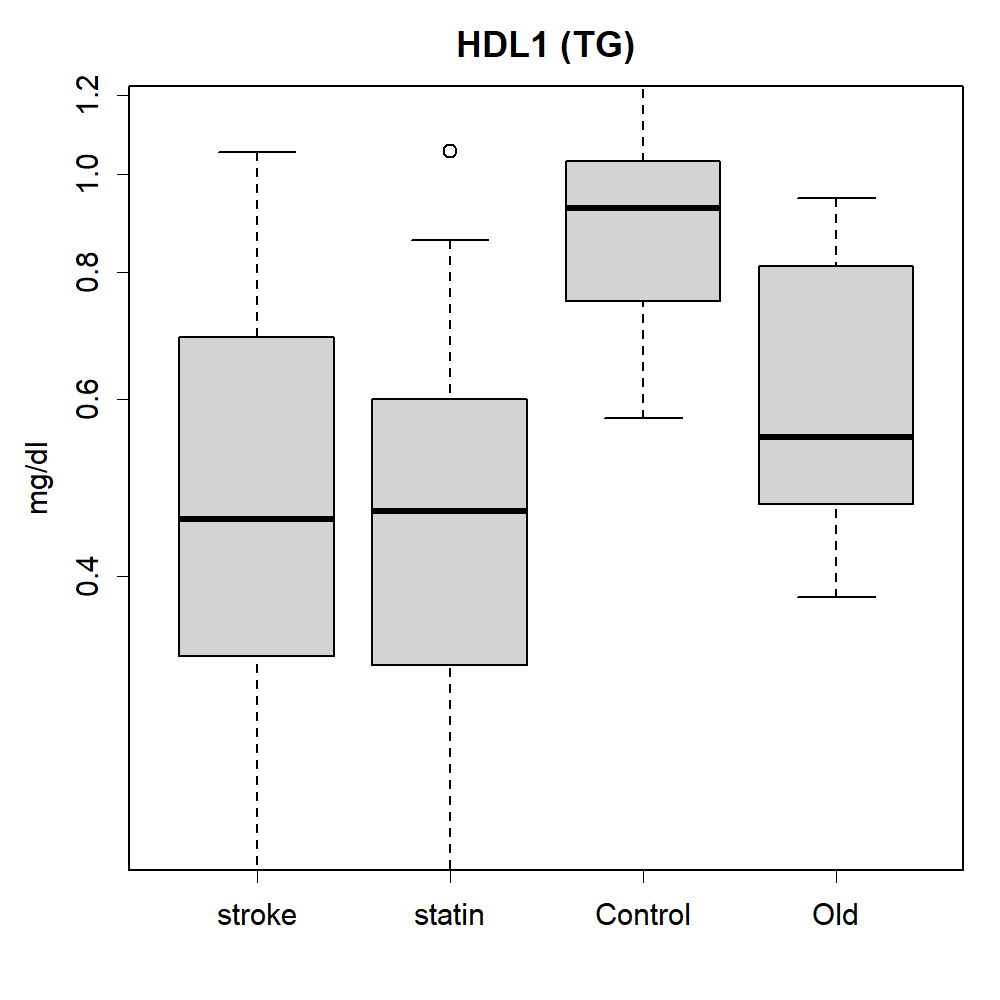

Supplement: S1 File — (ZIP) [file pone.0283855.s001.zip › supplement/TG/HDL1.png]

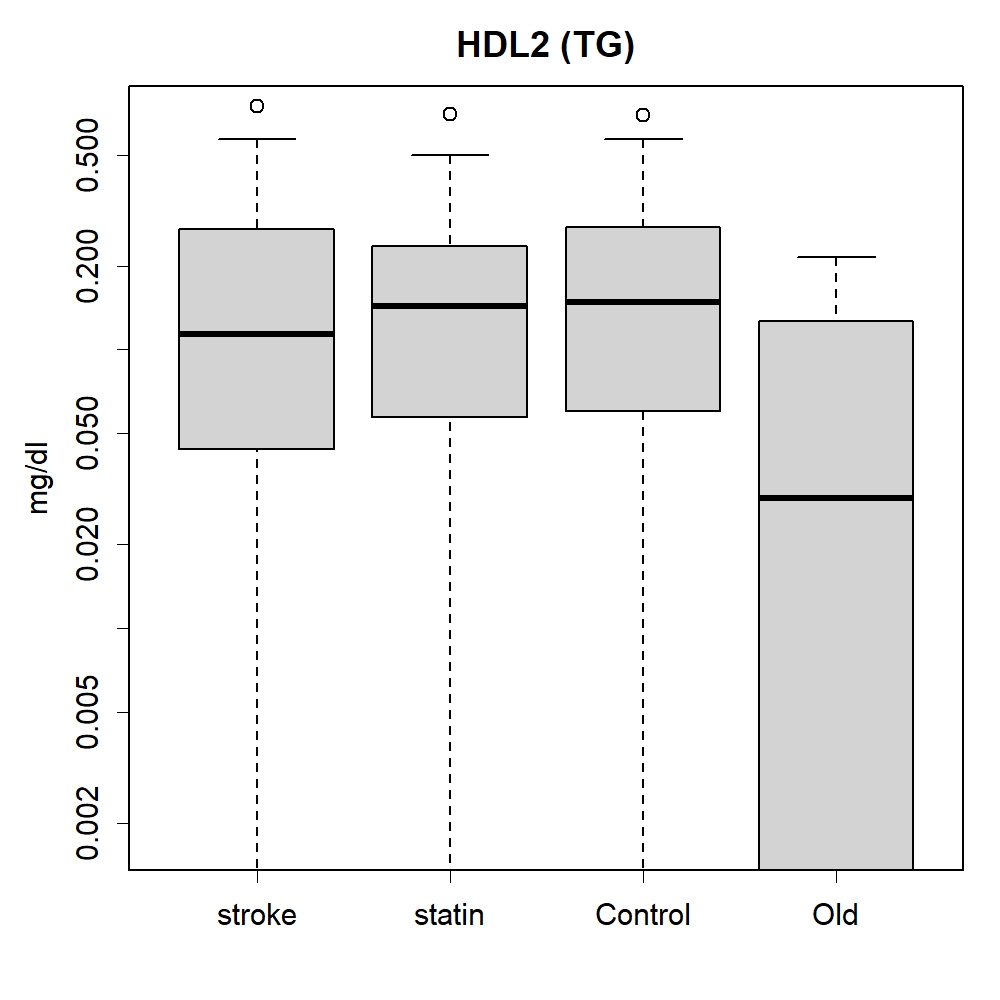

Supplement: S1 File — (ZIP) [file pone.0283855.s001.zip › supplement/TG/HDL2.png]

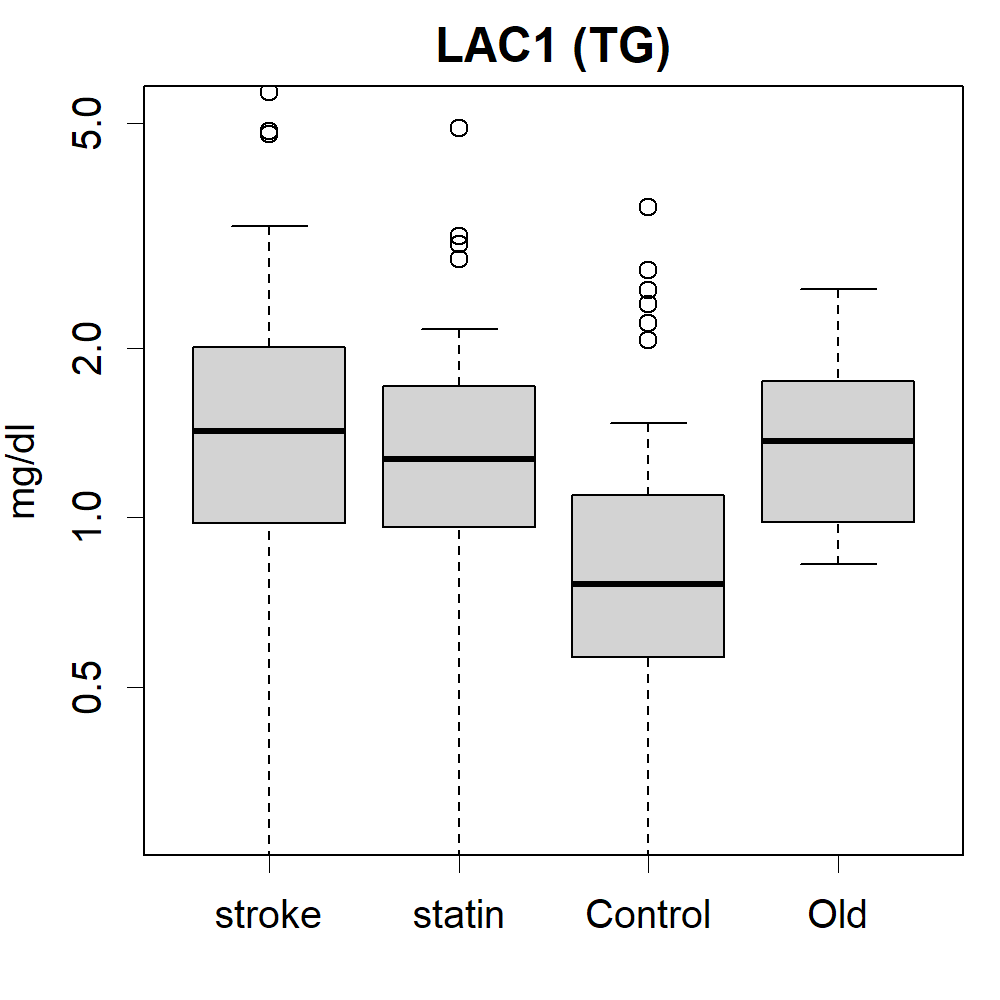

Supplement: S1 File — (ZIP) [file pone.0283855.s001.zip › supplement/TG/LAC1.png]

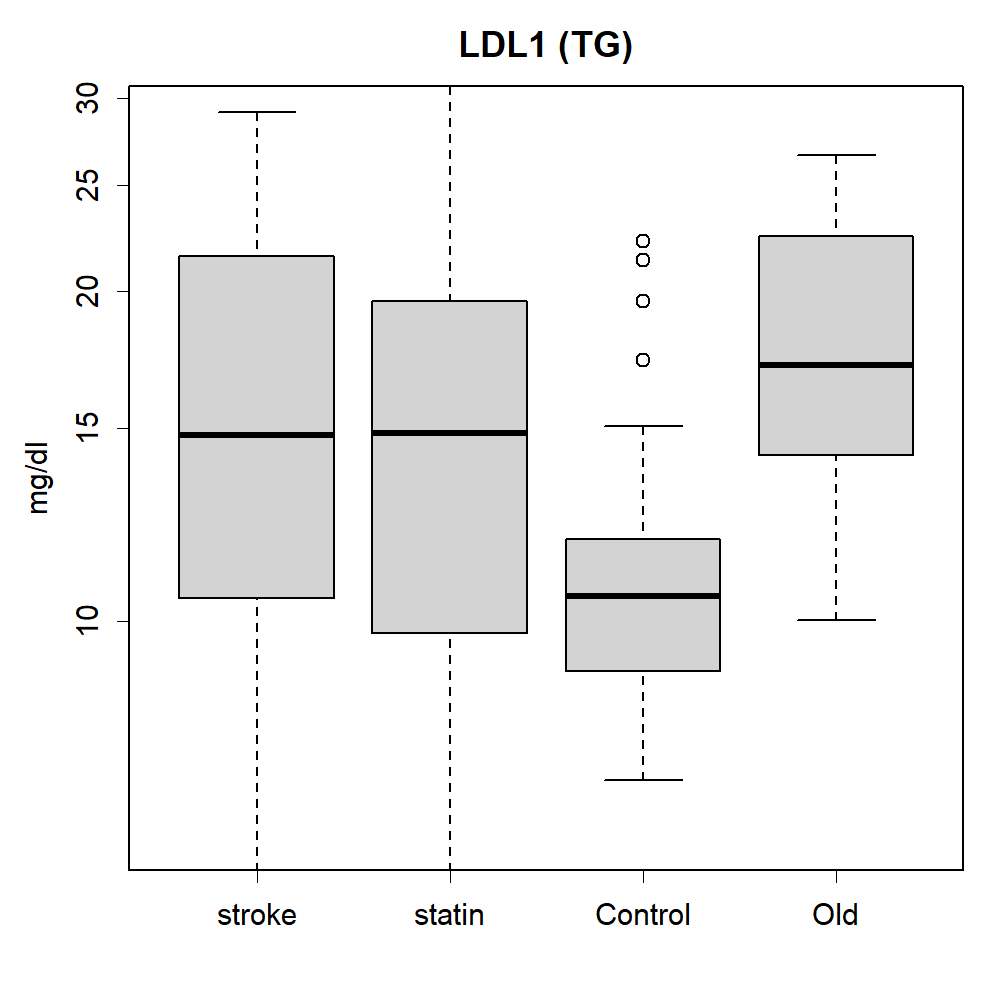

Supplement: S1 File — (ZIP) [file pone.0283855.s001.zip › supplement/TG/LDL1.png]

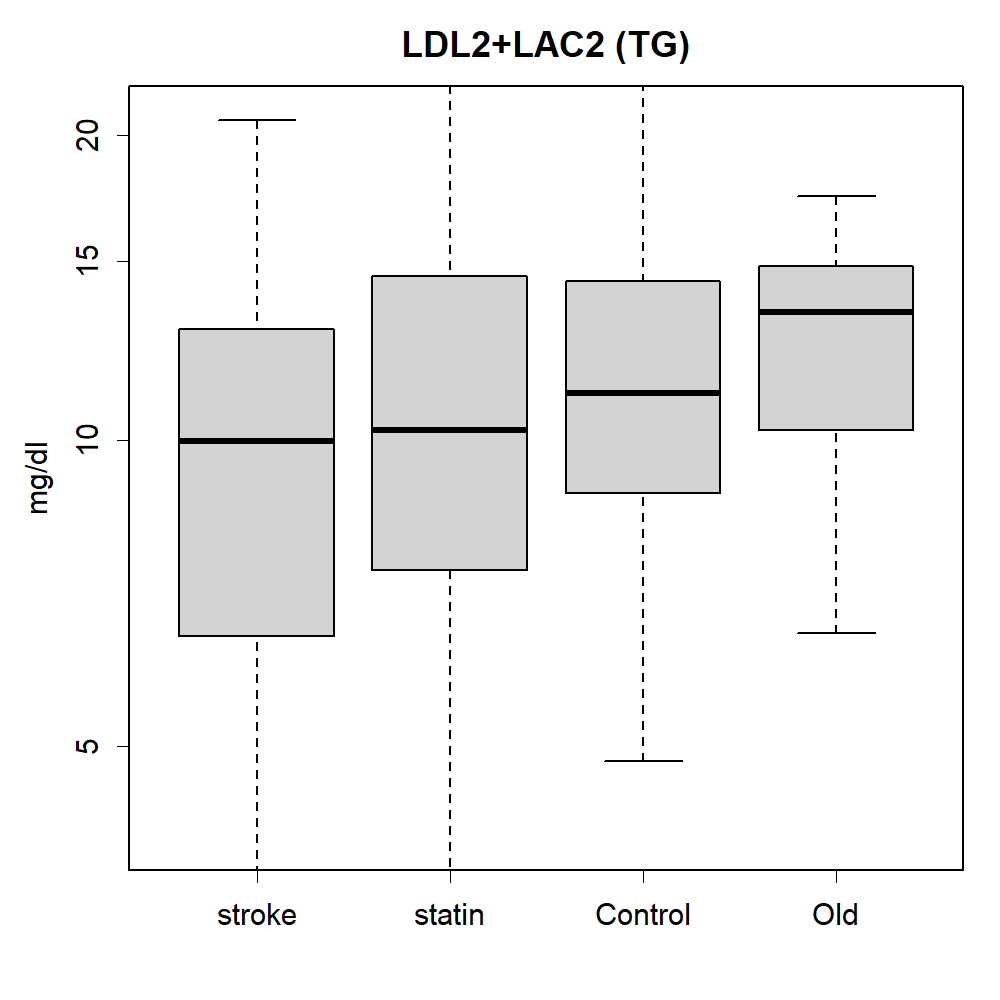

Supplement: S1 File — (ZIP) [file pone.0283855.s001.zip › supplement/TG/LDL2+LAC2.png]

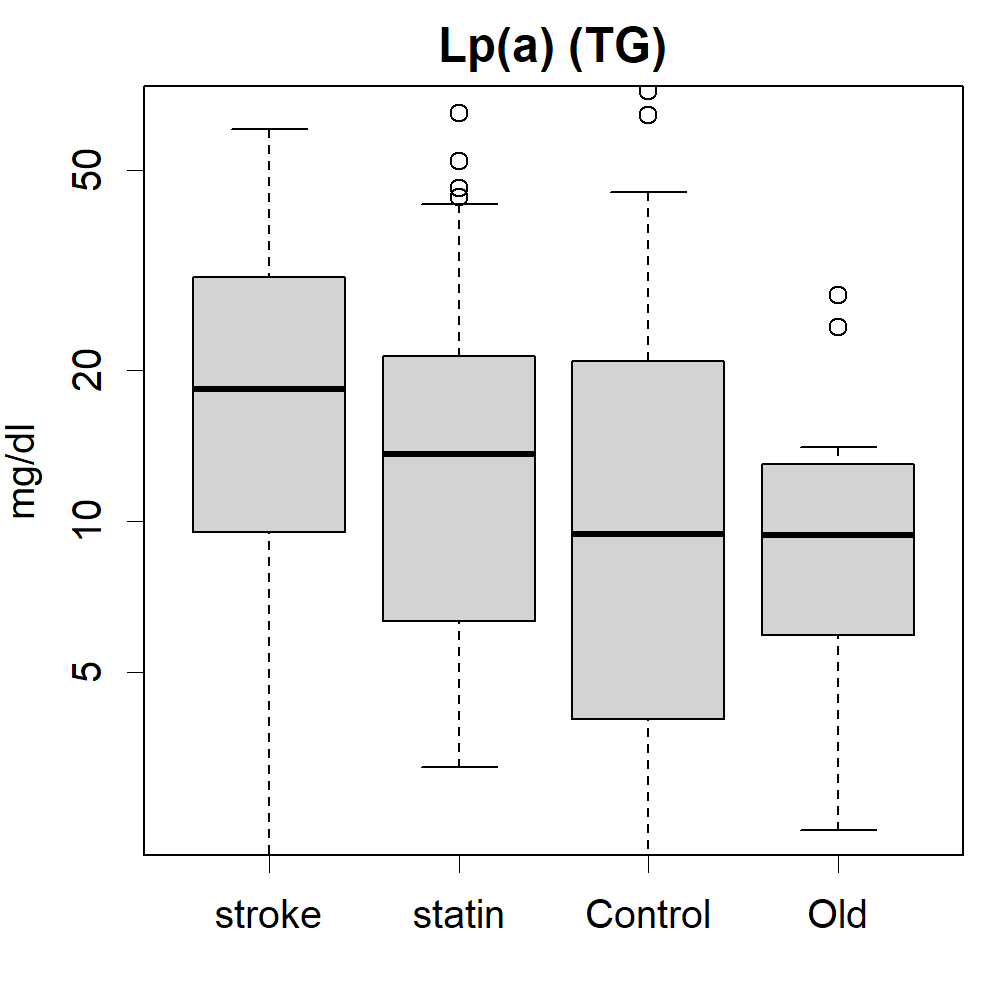

Supplement: S1 File — (ZIP) [file pone.0283855.s001.zip › supplement/TG/Lp(a).png]

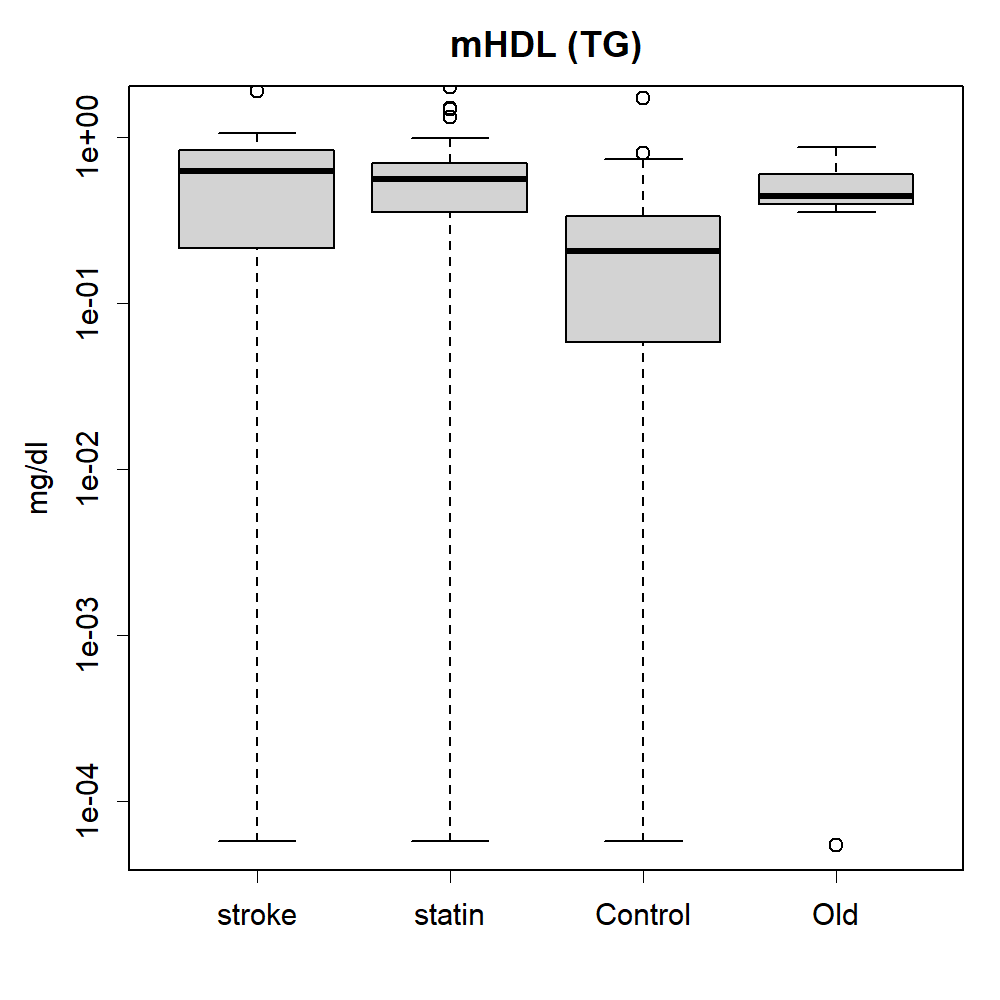

Supplement: S1 File — (ZIP) [file pone.0283855.s001.zip › supplement/TG/mHDL.png]

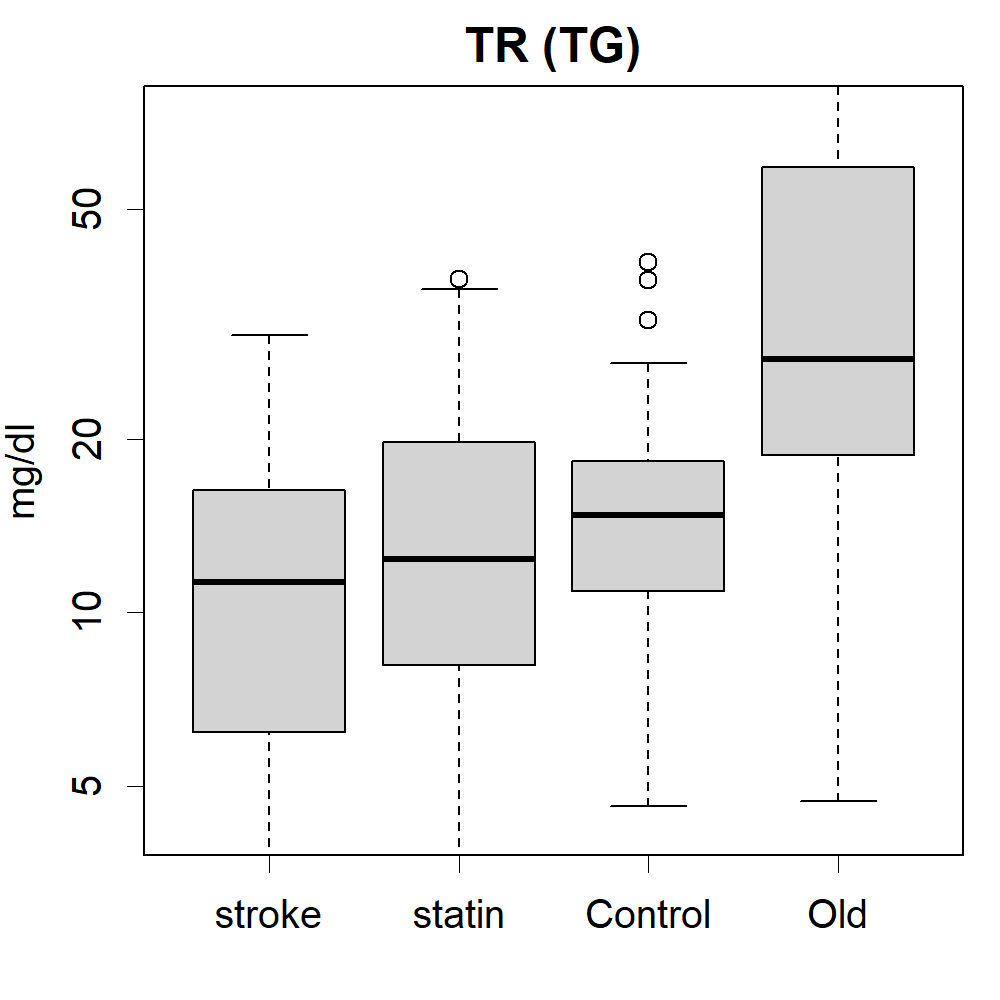

Supplement: S1 File — (ZIP) [file pone.0283855.s001.zip › supplement/TG/TR.png]

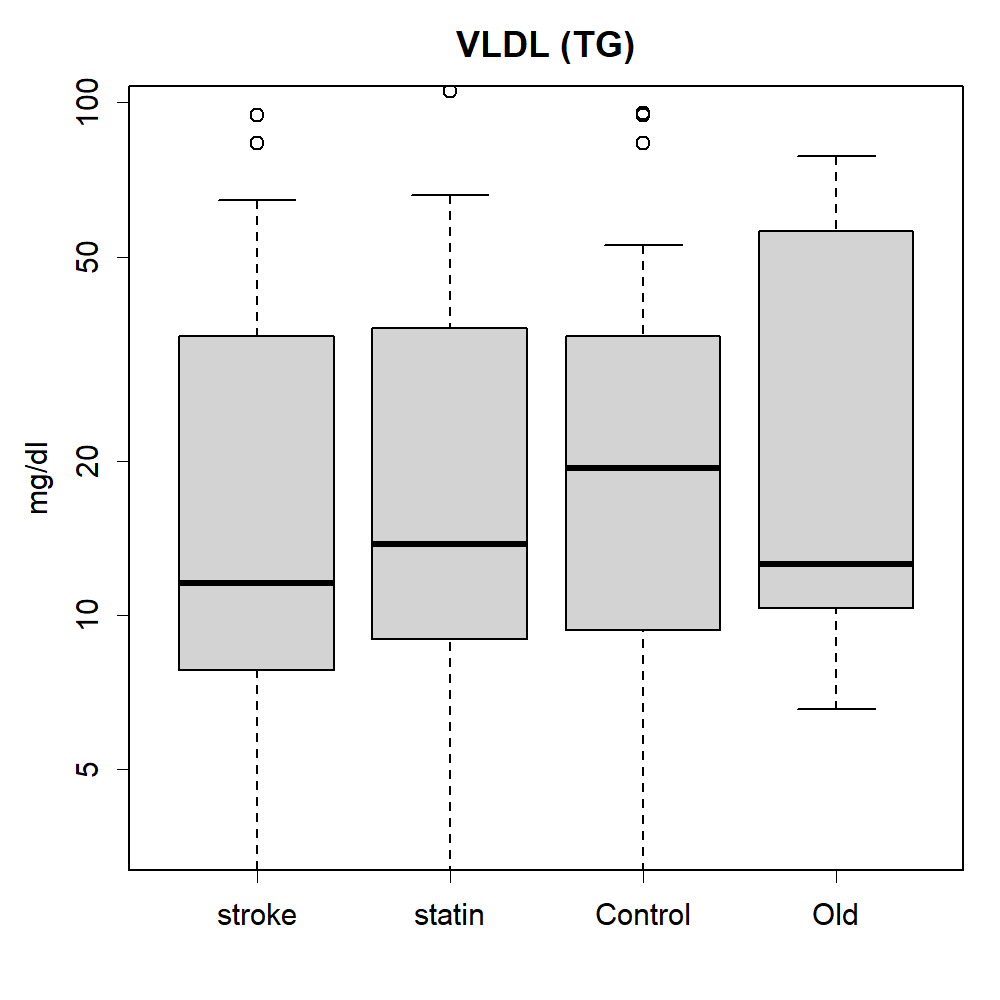

Supplement: S1 File — (ZIP) [file pone.0283855.s001.zip › supplement/TG/VLDL.png]
