# Supplementary material for: Distinctive features of lipoprotein profiles in stroke patients
Source: PLoS One. 2023 Apr 5;18(4):e0283855. doi: 10.1371/journal.pone.0283855 (PMC10075468; doi:10.1371/journal.pone.0283855)
Supplement: Attachment — Submitted filename: Response to Reviewers.docx [file pone.0283855.s002.docx]

Dear Dr Aktas,	10 Feb 2023

Thank you for reviewing our article. Below is the response.

1. Please ensure that your manuscript meets PLOS ONE's style requirements, including those for file naming. The PLOS ONE style templates can be found at

https://journals.plos.org/plosone/s/file?id=wjVg/PLOSOne_formatting_sample_main_body.pdf and

<https://journals.plos.org/plosone/s/file?id=ba62/PLOSOne_formatting_sample_title_authors_affiliations.pdf>"

Some of the "Figs. " were changed to "Figs “.

2. Please provide additional details regarding participant consent. In the ethics statement in the Methods and online submission information, please ensure that you have specified (1) whether consent was informed and (2) what type you obtained (for instance, written or verbal, and if verbal, how it was documented and witnessed). If your study included minors, state whether you obtained consent from parents or guardians. If the need for consent was waived by the ethics committee, please include this information.

Consent is explained verbally and written consent is received in writing. This statement was added in the Materials and Methods section. Minors were not included.

If you are reporting a retrospective study of medical records or archived samples, please ensure that you have discussed whether all data were fully anonymized before you accessed them and/or whether the IRB or ethics committee waived the requirement for informed consent. If patients provided informed written consent to have data from their medical records used in research, please include this information.

All data was anonymized prior to analysis. This was also added.

Additional Editor Comments (if provided):

Reviewers suggest publication but also invite authors to revise the paper. Especially, methodological issues must be addressed.

Information was added in the Materials and Methods section.

Reviewer #2: This is an interesting paper in which authors are using an alternative method for analyzing lipoprotein levels in subjects compared to the conventional ones.

I have the following comments:

-It is paramount to add in the methods section and also in the table the number and the

clinical carachteristics of the subjects enrolled in this study (especially for patients). This could also allow to perform correlations with clinical data (even of not pertinent for this study, it will necessarily be for future Studies).

The controls were healthy people, but the patients came from various backgrounds, as they were emergency medical evacuees. For this, the S1 table contains all the patient's symptom records. The numbers were added to the Materials and Methods section.

-Overall I would slightly undertune the weight of the sentences used in the conclusion, because these data, yet quite solid, require further consolidation in major studies

As instructed, the expression was weakened from assertion to suggestion.

-please check for all the acronyms (e.g. PCA it is nto spelle dthe first time that is used)

Apologies, that was my mistake. The first one that appeared was L87; I moved the one that was in L111. Similarly, I corrected CM on L89 and TG on L85.

-Page 4 line 84 please correct "hearts"with "cardiac symptoms"

Thank you for pointing this out. I have corrected it.

Reviewer #3: This is a retrospective study comparing stroke patients with controls. In addition, this study leads to prospective studies. The authors found that the most common risk factor in this study was the decrease in HDL level and then the increase in LDL, LAC1 and TG levels. At the same time, the authors found that statins were ineffective and argued that guidelines for medical treatment should be reviewed. Again, the authors suggest in this article that Glycerol can be used to identify people at potential risk because it is easily measurable. This article is an article that will contribute to science and can be accepted.

Thank you very much, it is an honour.

Best Regards,

Tomokazu Konishi
